# Synthesis of Secondary Amines via Self-Limiting Alkylation

**DOI:** 10.1021/acs.orglett.4c01430

**Published:** 2024-06-04

**Authors:** Pritam Roychowdhury, Saim Waheed, Uddalak Sengupta, Roberto G. Herrera, David C. Powers

**Affiliations:** Department of Chemistry, Texas A&M University, College Station, Texas 77843, United States

## Abstract

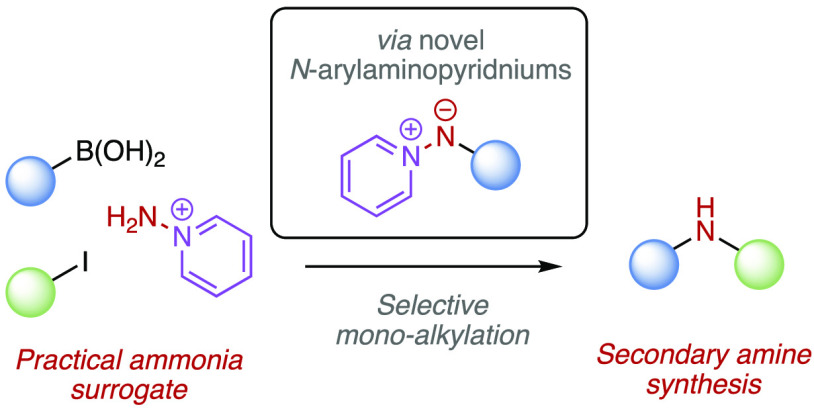

*N*-centered nucleophilicity increases upon alkylation, and thus selective partial alkylation of ammonia and primary amines can be challenging: Poor selectivity and overalkylation are often observed. Here we introduce *N*-aminopyridinium salts as ammonia surrogates for the synthesis of secondary amines via self-limiting alkylation chemistry. Readily available *N*-aryl-*N*-aminopyridinium salts engage in *N*-alkylation and *in situ* depyridylation to afford secondary aryl-alkyl amines without any overalkylation products. The method overcomes classical challenges in selective amine alkylation by accomplishing alkylation via transient, highly nucleophilic pyridinium ylide intermediates and can be applied in the context of complex molecular scaffolds. These findings establish *N*-aminopyridinium salts as ammonia synthons in synthetic chemistry and a strategy to control the extent of amine alkylation.

*N*-alkylation chemistry,^1−3^ which leverages the intrinsic *N*-centered nucleophilicity of trivalent nitrogen to forge new C–N bonds, is among the first reactions taught in introductory chemistry courses and comprises ∼10% of all transformations carried out in pharmaceutical research and development.^4^ Despite the ubiquity of *N*-alkylation chemistry, significant challenges remain: *N-*centered nucleophilicity increases upon alkylation, which renders partial alkylation, for example, to selectively access secondary amines, difficult (Figure 1a).^5^ In addition, substitution chemistry is often accompanied by competing elimination processes. Overalkylation and competitive elimination are particularly pronounced for the application of ammonia alkylation in selective synthesis. To overcome some of these challenges, ammonia surrogates—phthalimides,^6^ sulfonamides,^7^ dioxazolones,^8^ benzotriazoles,^9^ and hydroxylamine derivatives^10^—have been developed. While new amination reactions have been enabled by these reagents, the downstream chemistry is typically limited to deprotection to afford primary amines.

**Figure 1:**
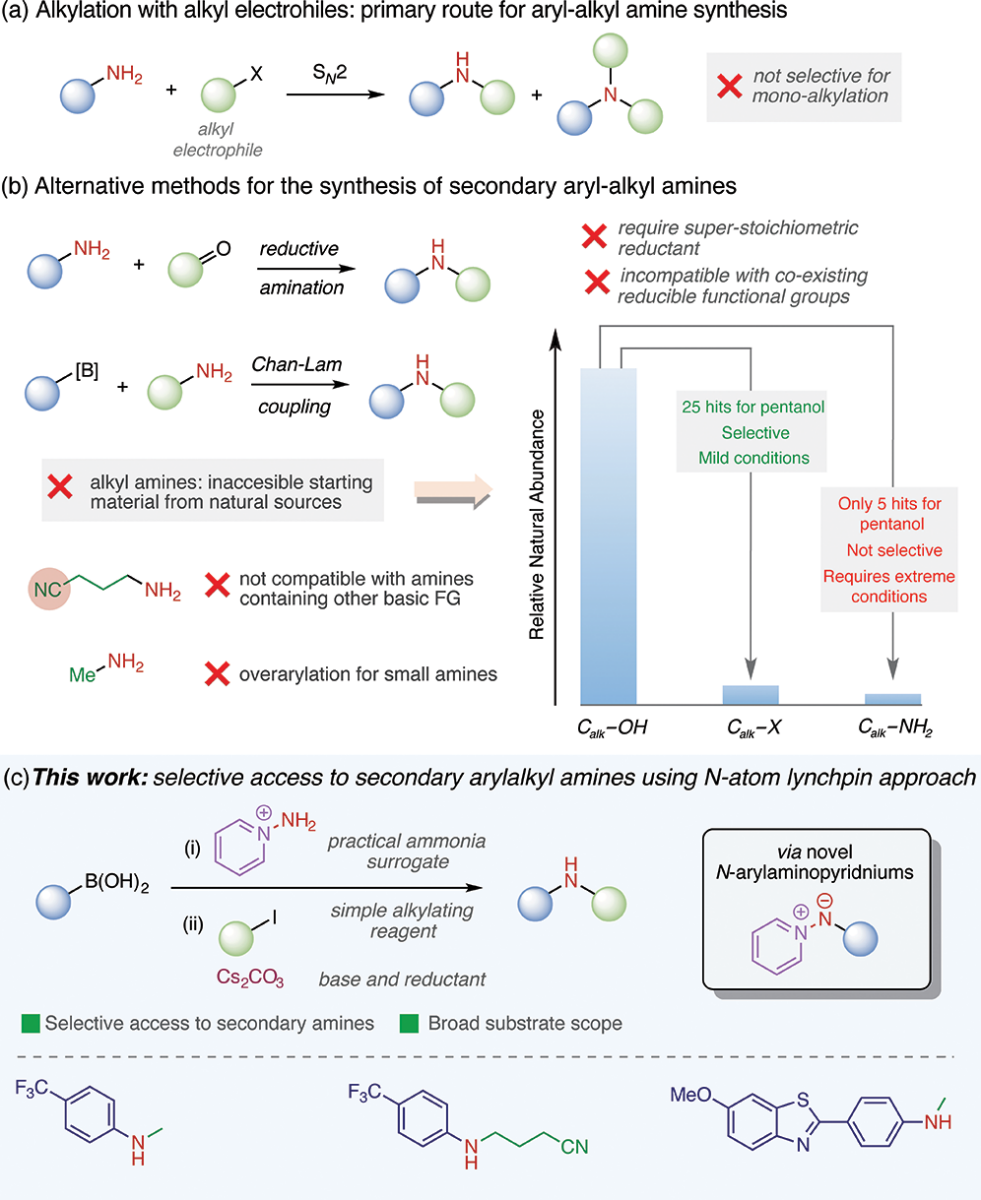
(a) Amine alkylation with alkyl electrophiles is the most common route to secondary aryl-alkyl amines. (b) Reductive amination and C–N cross coupling are popular approaches for secondary aryl-alkyl amine synthesis. Alkyl electrophiles are readily available from natural sources. (C) Here, we demonstrate self-limiting alkylation of *N*-aminopyridinium salts enables selective synthesis of secondary amines.

In response to the intrinsic challenges of partial *N-*alkylation, an arsenal of methods, including reductive amination and metal-catalyzed C–N coupling, have been developed.^11−14^ While these methods provide access to large families of amine products, *N-*alkylation, reductive amination, and C–N cross-coupling all require the use of amine-containing starting materials. These amine-containing starting materials are synthesized through several steps from naturally occurring feedstocks (e.g., alcohols and olefins), which can render them inconvenient starting materials. Thus, complementary methods, for example, that enable selective *N*-functionalization of amine surrogates with alkyl electrophiles, are of interest (Figure 1b).^15−20^

Previously, we demonstrated the synthesis of secondary aryl-alkyl amines via C–H *N-*aminopyridylation followed by Ni-catalyzed cross-coupling with aryl boronic acids.^23^ Although the scope of the C–N cross-coupling chemistry was broad, the initial C–H aminopyridylation exhibited significant limitations, notably working only with activated C–H bonds.

Motivated by the fundamental chemical challenges associated with selective partial ammonia alkylation and the ongoing need for new synthetic methods to access secondary amines, here we demonstrate that *N*-aminopyridinium reagents are useful ammonia synthons in the selective construction of secondary aryl-alkyl amines. We describe the monoalkylation of *N*-aryl-*N*-aminopyridinium derivatives with readily available alkyl halides. We term this process as *self-limiting alkylation* because in contrast to classical *N*-alkylation chemistry, in which *N*-alkylation results in a *more reactive nucleophile*, our method achieves monoalkylation by accomplishing alkylation via a highly nucleophilic pyridinium ylide. Following alkylation, the obtained *N*-alkyl-*N*-pyridinium amine is a *less reactive nucleophile.* These results provide new modular disconnections to rapidly assemble secondary amines, extend the burgeoning chemistry of *N*-aminopyridinium salts as bifunctional amine synthons,^21−27^ and introduce self-limiting alkylation as a conceptual approach for selective synthesis of secondary amines (Figure 1c).

We initially envisioned a two-step protocol for secondary amine synthesis based on sequential *N*-arylation and *N*-alkylation of *N*-aminopyridinium salts and set out to identify the conditions for each of these transformations. Previous reports of *N*-arylation of *N*-aminopyridinium via either S_N_Ar- or Pd-catalyzed coupling were limited to electron-deficient, heteroaryl halide coupling partners.^21,28^ Work from our laboratory in C–H amination^23^ and olefin aziridination^22^ efforts suggested that *N*-aminopyridinium salts can serve as plug-in replacements for sulfonamide reagents in many amination protocols. To access a family of *N*-aryl-*N*-aminopyridinium derivatives **3** needed to explore self-limiting alkylation chemistry, we extended the sulfonamide-to-aminopyridinium analogy to achieve highly efficient CuF_2_-catalyzed Chan–Lam cross-coupling^29,30^ of *N*-aminopyridinium salts and with aryl boronic acids (**2**) to afford a family of *N*-aryl-*N*-aminopyridinium salts (**3**) (Figure 2; see the Supporting Information for optimization details). In no case were products of double arylation observed, and the procedure could be readily translated to gram-scale synthesis: Compound **3a** was prepared in 71% yield on an 8 mmol scale (2.2 g product). With access to a family of *N-*aryl-*N*-pyridinium amines, including those derived from electron-deficient, -neutral, and -rich substrates as well as pharmaceutically derived precursors, we turned our attention to developing alkylation chemistry that would provide access to secondary aryl-alkyl amines.

**Figure 2:**
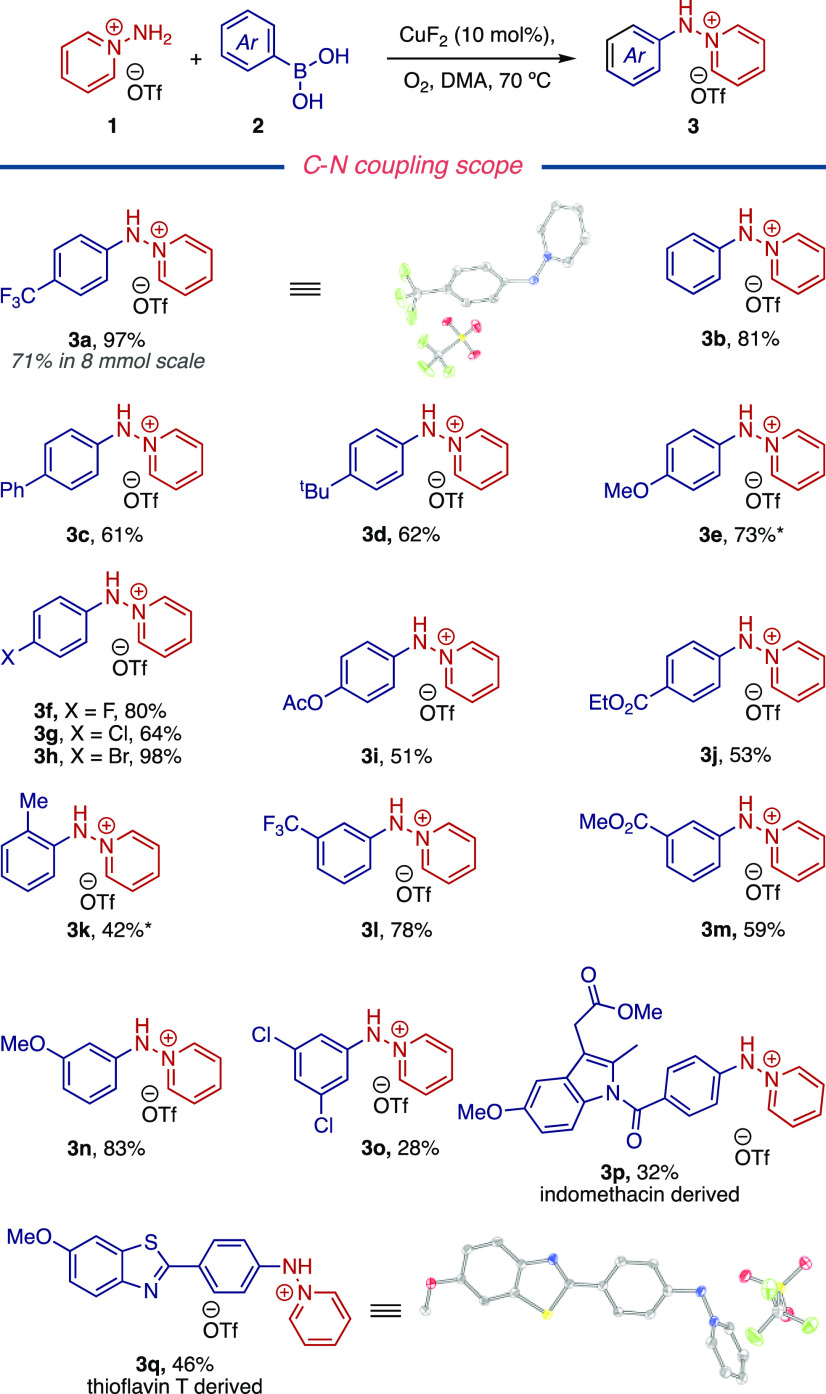
Cu-catalyzed coupling of **1** with aryl boronic acids (**2**) to afford *N*-arylaminopyridinium salts (**3**). Conditions: **1** (1.0 equiv), **2** (2.0 equiv), CuF_2_ (10 mol %), DMA (2 M), O_2_, 70 °C; * Conditions: **1** (1.0 equiv), **2** (3.0 equiv), CuF_2_ (10 mol %), *N*^1^,*N*^2^-di([1,1′-biphenyl]-2-yl)benzene-1,2-diamine (20 mol %), DMA (2 M), O_2_, 70 °C.

Using salt **3a**, we rapidly identified conditions for a one-step *N*-alkyation, depyridylation cascade that delivered secondary amines selectively. Treatment of a MeCN solution of **3a** with hexyl iodide **4a** and CsOAc at 70 °C resulted in *N*-alkylation to pyridinium amine **5a′** in 98% yield (Table 1). During optimization studies, we observed that, while carboxylate and bicarbonate bases afforded alkylated product **5a′** (Table 1, entries 1 and 2), *tert*-butoxide or carbonate bases afforded secondary amine **5a**, the product of *in situ* depyridylation of **5a′**, directly (entries 3–5). Ultimately, Cs_2_CO_3_ was identified as the optimized base and promoted a one-pot alkylation/depyridylation sequence to furnish secondary amine **5a** in 79% yield (see below for a discussion of the depyridylation mechanism).

**Table 1:**
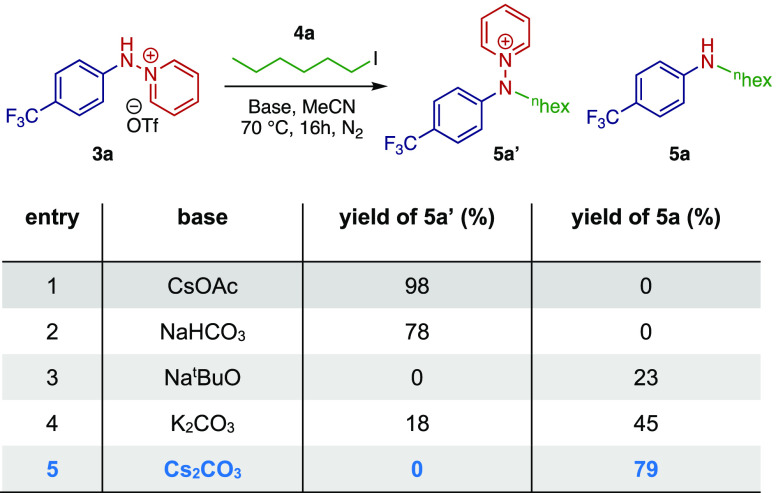
Optimization of the Alkylation–Depyridylation Protocol^a^

a Conditions: **3a** (1.0 equiv), 1-iodohexane (**4a**, 2.0 equiv), base (3.0 equiv), CH_3_CN, 70 °C, 16 h; yields are measured using 1,3,5-trimethoxybenzene as the internal standard.

The developed alkylation–depyridylation protocol provided access to a broad array of secondary aryl-alkyl amines (Figure 3). Diverse functional groups, including long alkyl chains (**5a** and **5b**), cyanides (**5c**), amides (**5e**), and protected alcohols (**5f**), are well-tolerated. The reaction could be accomplished from the corresponding alkyl bromide or triflate, as demonstrated for the synthesis of **5a**, in 63% and 49% yield, respectively. While secondary allylic and benzylic iodides engage in efficient alkylation, as demonstrated by the examples **5g** (81%) and **5h** (62%), unactivated secondary alkyl iodides do not engage in alkylation (i.e., **5i**). For highly electrophilic starting materials, e.g., **4j**–**4m**, a lower reaction temperature was used to prevent overalkylation: methyl iodide engaged in selective monoalkylation to afford **5j** in 96% yield; primary benzyl iodides **4k** and **4l** were converted to secondary amines **5k** and **5l** in 98% and 60% yield, respectively; and primary allyl iodide **4m** afforded secondary amine **5m** in 43% yield.

**Figure 3:**
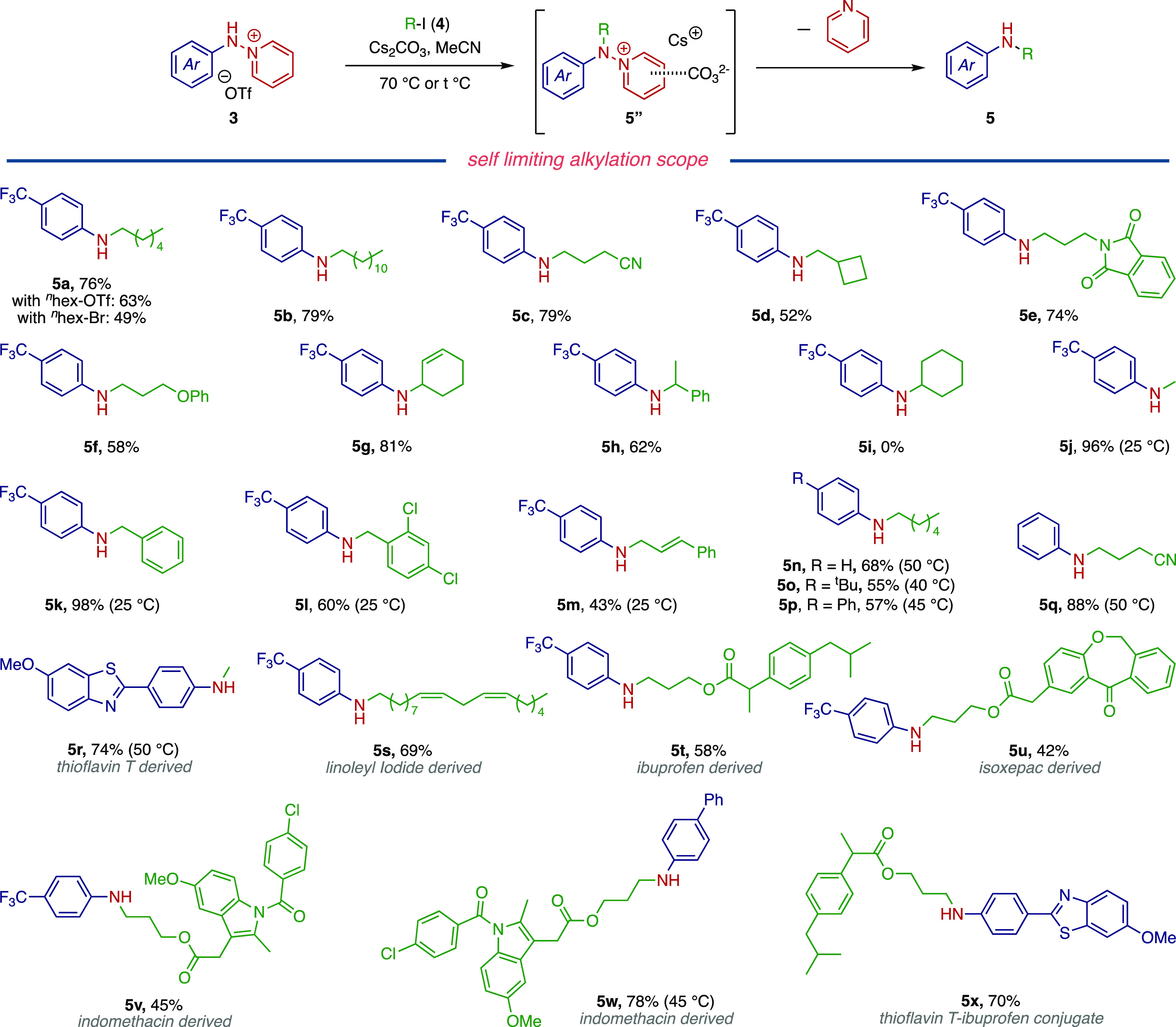
Monoalkylation of **3**. Conditions: **3** (1.0 equiv), **4** (2.0 equiv), Cs_2_CO_3_ (3.0 equiv), CH_3_CN, *t* °C, 16 h; yields are isolated.

With respect to the *N*-arylaminopyridinium reaction partner (**3**), both electron-neutral and electron-donating (**5n**–**5q**) aminopyridinium derivatives were converted to their respective secondary aryl-alkyl amines efficiently. Moreover, thioflavin T-derived *N*-aminopyridnium derivative **3p** was converted to methylated amine **5r**, which is used in investigational studies of Alzheimer’s disease, in 74% yield.

The developed alkylation/depyridylation chemistry can be implemented in the context of natural products and drug molecules. Linoleyl iodide was transformed to **5s** in 69% yield. Ibuprofen-derived alkyl iodide was converted to amine **5t** in a 58% yield. Isoxepac-derived and indomethacin-derived alkyl iodides were successfully coupled to give the corresponding products **5u** and **5v** in 42% and 45% yields, respectively. Biphenyl-derived aminopyridinium **3c** can also be coupled with indomethacin-derived alkyl iodide **4v** to give the corresponding product **5w** in 78% yield. The thioflavin T-derived *N*-aminopyridinum salt **3p** can be coupled with ibuprofen-derived alkyl iodide **4t** to yield drug conjugate **5x** in 70% yield. These examples highlight the compatibility of C–N bond construction with pharmaceutically relevant basic heterocycles, amides, carbamates, and basic amines.

With robust conditions for self-limiting alkylation in hand, we turned our attention to understanding (1) the origins of the observed partial alkylation and (2) the mechanistic basis for *in situ* depyridylation. The following mechanistic studies were carried out with *N*-pyridinium salt **3a**, but importantly, electron-withdrawing groups are not needed for efficient depyridylation (i.e., **5n**, **5o**, **5p**, and **5x**).

To investigate the origin of the observed partial alkylation selectivity, we treated an independently prepared sample of ylide **3a′** with alkyl iodide **4a** and observed the rapid formation of alkylation pyridinium salt **5a′** (Figure 4a, see Section C.1 of the Supporting Information). In contrast, neither *p*-trifluoromethyl aniline **6** nor amine **5a** undergo deprotonation in the presence of Cs_2_CO_3_ nor undergo alkylation to an appreciable extent upon subsequent exposure to **4a** (Figure 4b, see Section C.3 of the Supporting Information). These data indicate that the presence of an electron-withdrawing pyridinium substituent in **3a**, which lowers the N–H p*K*_a_ and enables access to ylide **3a′**, is critical to efficient alkylation: Ylide **3a′** is more nucleophilic than amine **6** and thus undergoes alkylation under conditions that **6** is unreactive. In contrast to typical amine alkylation reactivity trends, *in situ* depyridylation renders the products of alkylation (i.e., **5a**) *less nucleophilic* than the starting material (i.e., ylide **3a′**).

**Figure 4:**
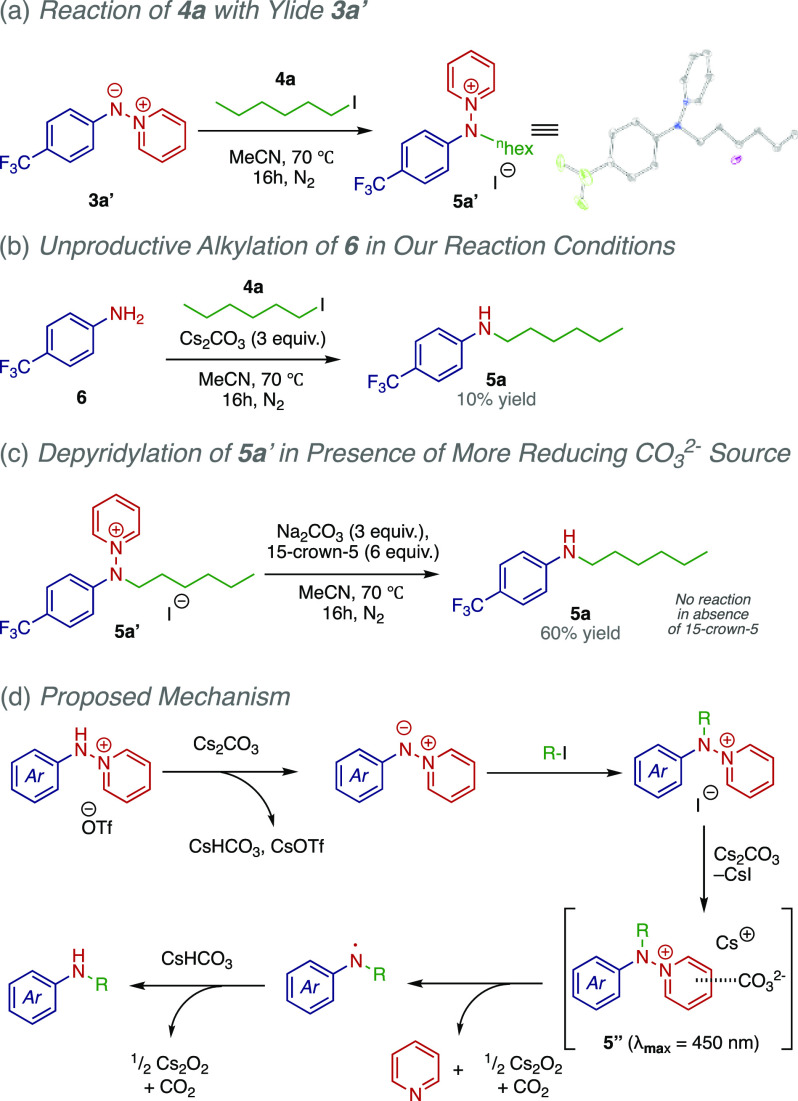
(a) Reaction of ylide **3a′** with **4a** yields intermediate **5a′**. (b) Aniline **6** does not undergo alkylation. (c) Depyridylation of **5a′** proceeds with Na_2_CO_3_ and 15-crown-5. (d) Proposed mechanism for the alkylation/depyridylation sequence.

To investigate the mechanism of the unanticipated *in situ* depyridylation, we treated an independently synthesized sample of compound **5a′** with each of the reagents present during the alkylation reaction (i.e., **3a**, **3a′**, **4a**, and Cs_2_CO_3_; see Section C.4 of the Supporting Information). Of these reactions, treatment of **5a′** with excess Cs_2_CO_3_ uniquely resulted in depyridylation to afford **5a** in 79% yield along with pyridine (26%) and pyridine-derived products (see Section C.5 of the Supporting Information). In addition, while the one-pot alkylation/depyridylation protocol described above utilizes 3 equiv of Cs_2_CO_3_, alkylation of **3a** in the presence of 1 equiv of Cs_2_CO_3_ resulted in alkylated pyridinium amine **5a′** (78%), not secondary amine **5a** (see Section C.6 of the Supporting Information). Based on these observations, we hypothesize that carbonate serves two roles: as a base to generate nucleophilic pyridinium ylides and as a reductant to promote *in situ* depyridylation.^31^

Consistent with the carbonate-as-reductant hypothesis, while Na_2_CO_3_ does not promote efficient depyridylation of **5a′**, addition of 15-cr-5 to sequester Na^+^ and generate a more reducing carbonate source, promotes efficient depyridylation (60% yield of **5a**, Figure 4c). Analysis of the reaction headspace revealed the formation of CO_2_ during successful deprotection reactions, whereas unproductive conditions (i.e., Na_2_CO_3_ without 15-cr-5) did not evolve CO_2_ (see Section C.7 of the Supporting Information). Finally, exposure of pyridinium salt **5a′** to Cs_2_CO_3_ results in the formation of a low-energy absorbance centered at 450 nm (see Section C.7 of the Supporting Information). This observation is consistent with the formation of a carbonate-to-pyridinium electron donor–acceptor (EDA) complex (**5″**).^32−35^ Attempts to trap the putative radical intermediate with common EPR spin traps, such as *N-tert*-butyl-α-phenylnitrone (PBN), were unsuccessful, which is consistent with the expected short lifetime of this reactive intermediate. Together, these data indicate that Cs_2_CO_3_ promotes depyridylation of alkylated pyridinium amine **5a′** via thermally promoted electron transfer within a discrete EDA complex.

In conclusion, we introduce self-limiting alkylation chemistry as a platform for partial amine alkylation. We describe a one-pot synthesis of secondary amines via the self-limiting alkylation of *N-*aryl*-N-*aminopyridinium salts. Chan–Lam coupling of aryl boronic acids with *N*-aminopyridinium triflate provides access to a diverse set of *N*-aryl-*N-*pyridinium amines. Deprotonation of these salts affords highly nucleophilic pyridinium ylides that engage in facile substitution chemistry with alkyl halides. The resulting pyridinium salts are much less nucleophilic than the ylide precursor, which enforces selective monoalkylation. *In situ* reductive cleavage of the N–N bond (i.e., removal of the pyridinium moiety) affords secondary amines. Mechanistic experiments suggest that this unique depyridylation is triggered by the transfer of electrons from Cs_2_CO_3_. Notably, Cs_2_CO_3_ serves a dual function, acting as both a base and a reductant in this transformation. The *N*-arylation, *N*-alkylation sequence can be applied in the context of complex, pharmaceutically relevant molecules. Unlike amination methods that necessitate amine-based starting materials, the presented method harnesses readily available alkyl electrophiles in partial alkylation chemistry. The resulting method represents a new approach to secondary amines and validates *N-*aminopyridinium compounds as ammonia surrogates in synthetic chemistry.^36^

## Data Availability

The data underlying this study are available in the published article and its Supporting Information.

## Supporting Information Available

Experimental methods, optimization data, spectral data, experimental spectra, and diffraction data (PDF)
