# Supplementary material for: Synthesis of Secondary Amines via Self-Limiting Alkylation
Source: Org Lett. 2024 Jun 4;26(23):4926–31. doi: 10.1021/acs.orglett.4c01430 (PMC11187628; doi:10.1021/acs.orglett.4c01430)
Supplement: Supplementary file 1 — ol4c01430_si_001.pdf [file ol4c01430_si_001.pdf]

Supporting Information

Synthesis of Secondary Amines via Self-Limiting Alkylation

Pritam Roychowdhury, Saim Waheed, Uddalak Sengupta, Roberto G. Herrera, and David C. Powers*

Department of Chemistry, Texas A&M University, College Station, Texas 77843, USA

Email: powers@chem.tamu.edu

Table of Contents

A. General Considerations S3
A.1 Materials S3
A.2 Characterization Details S3
A.3 X-Ray Diffraction Details S4

B. Synthesis and Characterization S5
B.1. Chan-Lam Coupling of Boronic Acids and *N*-Aminopyridinium Triflate S5
B.1.1 Gram-Scale Synthesis of **3a** S11
B.2 Alkylation of *N*-Aminopyridinium Derivatives S12
B.3 One-Pot Reaction Protocol for the Synthesis of **5b** S19

C. Mechanistic Studies S20
C.1 Synthesis of Ylide **3a'** S20
C.2 Reaction of 1-Iodohexane with Ylide **3a'** S20
C.3 Unproductive Alkylation of **6** or **5a** S20
C.4 Alkylation of **3a** with 1-Iodohexane using CsOAc S21
C.5 Removal of Pyridine from **5a'** using Excess Cs_2CO_3 S21
C.6 Alkylation of **3a** with 1-Iodohexane using 1 Equivalent of Cs_2CO_3 S22
C.7 Depyridylation of **5a'** in Presence of Reducing CO_3^{2-} Source S22

D. X-ray Diffraction Data S25

E. Additional Data S31
E.1 Optimization Studies for the C(sp2) *N*-Aminopyridylation S31
E.2 Summary of Unproductive Substrates S33

F. NMR Spectra for New Compounds S34

G. References S93

A. General Considerations

A.1 Materials All chemicals and solvents were obtained as ACS reagent grade and used as received. Copper(II) fluoride was acquired from Strem Chemicals. (4-Fluorophenyl)boronic acid, (4-chlorophenyl)boronic acid, (3,5-dichlorophenyl)boronic acid, *o*-tolylboronic acid and (4-(ethoxycarbonyl)phenyl)boronic acid were purchased from Matrix Scientific. Phenylboronic acid, (4-(*tert*-butyl)phenyl)boronic acid, (3-(methoxycarbonyl)phenyl)boronic acid, (4-acetoxyphenyl)boronic acid, methyl iodide, and 1-iodohexane were purchased from Tokyo Chemical Industry (TCI). *N,N*-Dimethylacetamide (DMA), *p*-biphenylboronic acid, sodium bicarbonate, and potassium carbonate were acquired from Oakwood. 4-Bromophenylboronic acid, 3-methoxyphenylboronic acid, (4-methoxyphenyl)boronic acid, (3-(trifluoromethyl)phenyl)boronic acid, hexanes, ethyl acetate, dichloromethane, dimethyl sulfoxide (DMSO), *N*,*N*-dimethylformamide (DMF), sodium *tert*-butoxide, cesium acetate, cesium carbonate, copper(I) trifluoromethanesulfonate toluene complex, copper(II) bromide, copper(II) acetylacetonate, 4-pyridinylboronic acid, 2-thienylboronic acid, and iodocyclopentane were obtained from Sigma Aldrich. (4-(Trifluoromethyl)phenyl)boronic acid, 1-iodododecane, iodocyclohexane, cyclopropylboronic acid, and (1-(*tert*-butoxycarbonyl)-6-methoxy-1H-indol-2-yl)boronic acid were obtained from Ambeed, Inc. Anhydrous magnesium sulfate and anhydrous potassium carbonate were obtained from VWR. Acetonitrile and methanol were obtained from Fischer Scientific. Dry dichloromethane and acetonitrile (purchased from Fisher scientific, HPLC grade) was obtained from a drying column and stored over activated 4 Å molecular sieves.[1] NMR solvents were purchased from Cambridge Isotope Laboratories and were used as received. All reactions were carried out under ambient atmosphere unless otherwise noted.

N-Aminopyridinium triflate,[2] N^1,N^2-di([1,1'-biphenyl]-2-yl)benzene-1,2-diamine,[3] (4-(5-methoxy-3-(2-methoxy-2-oxoethyl)-2-methyl-1H-indole-1-carbonyl)phenyl)boronic acid (**2p**),[4] (4-(6-methoxybenzo[d]thiazol-2-yl)phenyl)boronic acid (**2q**),[5] 4-iodobutanenitrile (**4c**),[6] (iodomethyl)cyclobutane (**4d**),[7] 2-(3-iodopropyl)isoindoline-1,3-dione (**4e**),[7] (3-iodopropoxy)benzene (**4f**),[6] 3-iodocyclohex-1-ene (**4g**),[8] (1-iodoethyl)benzene (**4h**),[8] (iodomethyl)benzene (**4k**),[9] 2,4-dichloro-1-(iodomethyl)benzene (**4l**),[9] (*E*)-(3-iodoprop-1-en-1-yl)benzene (**4m**),[8] (6*Z*,9*Z*)-18-iodooctadeca-6,9-diene (**4s**),[7] 3-iodopropyl 2-(4-isobutylphenyl)propanoate (**4t**),[10] 3-iodopropyl 2-(11-oxo-6,11-dihydrodibenzo[b,e]oxepin-2-yl)acetate (**4u**)[10] and 3-iodopropyl 2-(1-(4-chlorobenzoyl)-5-methoxy-2-methyl-1H-indol-3-yl)acetate (**4v**)[10] were prepared according to literature procedures.

A.2 Characterization Details ^{1}H and ^{13}C NMR spectral acquisitions were recorded on an Inova 500 FT NMR (Varian), a VNMRS 500 FT NMR (Varian), or an Acsend™ 400 NMR (Bruker) and were referenced against residual proteo solvent signals: $CDCl_3$ (7.26 ppm, ^{1}H; 77.16 ppm, ^{13}C) and acetonitrile-d_3 (1.94 ppm, ^{1}H).[8] ^{1}H NMR data are reported as follows: chemical shift (δ, ppm), (multiplicity: s (singlet), d (doublet), t (triplet), m (multiplet), br (broad), integration). ^{13}C NMR data are reported as follows: chemical shift (δ, ppm). The ^{13}C signal corresponding to the OTf counter anion for the salts **3** is observed as a quartet with low intensity. The peak appears at 120.2 ppm (q, $^1J_{C\text{–}F}$ = 318 Hz).

Mass spectrometry data were recorded on either Orbitrap Fusion™ Tribrid™ Mass Spectrometer or Q Exactive™ Focus Hybrid Quadrupole-Orbitrap™ Mass Spectrometer from ThermoFisher Scientific. An Agilent Trace 1300 GC with attached thermal conductivity detector and a custom-made 120 cm stainless steel column packed with Carbosieve-II was used for analysis of head space gases. The column was kept at 200 °C and Ar was used as carrier gas. The detector was set to a temperature of 250 °C. Headspace gas (~300 μL) was transferred to the GC with a 0.50 mLValco Precision Sampling Syringe (SeriesA-2) equipped with a Valco Precision sampling needle with a 5-point side port.

A.3 X-Ray Diffraction Details Experimental details of crystallization are included in the synthetic procedures for the relevant compounds. A Bruker APEX 2 Duo X-ray (three-circle) diffractometer was used for crystal screening, unit cell determination, and data collection for the X-ray crystal structures of **3a**, **3q** and **5a'**. Crystal suitable for X-ray diffraction were mounted on a MiTeGen dual-thickness micro-mount and placed under a cold N_2 stream (Oxford). The X-ray radiation employed was generated from a Mo sealed X-ray tube ($K\alpha$ = 0.70173 Å with a potential of 40 kV and a current of 40 mA). Bruker AXS APEX II software was used for data collection and reduction. Absorption corrections were applied using the program SADABS. A solution was obtained using XT/XS in APEX2 and refined in Olex2.[11-13] Hydrogen atoms were placed in idealized positions and were set riding on the respective parent atoms. All non- hydrogen atoms were refined with anisotropic thermal parameters. The structure was refined (weighted least squares refinement on F2) to convergence.[13]

B. Synthesis and Characterization

B.1 Chan-Lam Coupling of Boronic Acids and *N*-Aminopyridinium Triflate

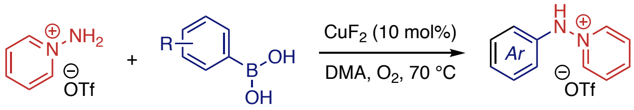

General Procedure A: A 4-mL scintillation vial was charged with *N*-aminopyridinium triflate (**1**, 48.8 mg, 0.200 mmol, 1.00 equiv), arylboronic acid **2** (0.200 mmol, 1.00 equiv), copper(II) fluoride (1.0 mg, 0.010 mmol, 0.050 equiv), *N,N*-dimethylacetamide (DMA, 0.1 mL). A magnetic stir bar was added, and the vial was fitted with a rubber septum. Oxygen was bubbled through the reaction mixture for 1 h at 23 °C. Under an O_2 atmosphere, the mixture was heated at 70 °C inside a reaction block for 19 h. The reaction mixture was cooled to 23 °C and O_2 was again bubbled through the reaction mixture for 1 h at 23 °C. Arylboronic acid **2** (0.200 mmol, 1.00 equiv) and copper(II) fluoride (1.00 mg, 0.010 mmol, 0.050 equiv) were added. Under an O_2 atmosphere, the mixture was heated at 70 °C inside a reaction block for an additional 19 h. The reaction mixture was cooled to 23 °C and DMA was removed under reduced pressure. The residue was purified by SiO_2 gel chromatography (eluent 100:0 CH_2Cl_2:MeOH to 95:5 CH_2Cl_2:MeOH) to afford the title compound. The obtained residue can be used for the next step without further purification or can be crystallized using a solvent mixture of MeCN/Et_2O. Characterization data for the resulting *N*-aryl-*N*-pyridinium amines (**3**) are collected below.

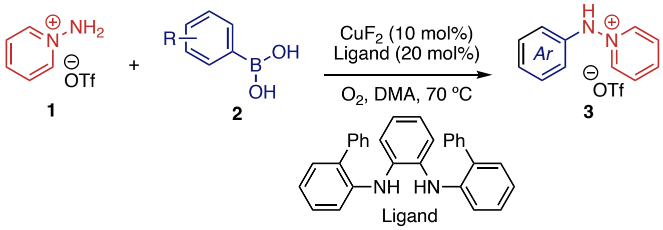

General Procedure B: A 4-mL scintillation vial was charged with *N*-aminopyridinium triflate **1** (48.8 mg, 0.200 mmol, 1.00 equiv), arylboronic acid **2** (0.300 mmol, 1.50 equiv), *N,N*-dimethylacetamide (DMA, 0.1 mL). A magnetic stir bar was added, and the vial was fitted with a rubber septum. Oxygen was bubbled through the reaction mixture for 1 h at 23 °C. In a N_2-filled dry box, copper(II) fluoride (1.0 mg, 0.010 mmol, 0.050 equiv) and N^1,N^2-di([1,1'-biphenyl]-2-yl)benzene-1,2-diamine (4.1 mg, 0.020 mmol, 0.10 equiv) were mixed together in DMA (20.0 µL) and stirred under N_2 for 30 min to give a purple solution. After this time the catalyst solution was removed from the dry box and added to the reaction mixture. Under an O_2 atmosphere, the mixture was heated at 70 °C inside a reaction block for 19 h. The reaction mixture was cooled to 23 °C and O_2 was again bubbled through the reaction mixture for 1 h at 23 °C. Arylboronic acid **2** (0.300 mmol, 1.50 equiv) and another equivalent of catalyst solution (prepared as before) were added. Under an O_2 atmosphere, the mixture was heated at 70 °C inside a reaction block for an additional 19 h. The reaction mixture was cooled to 23 °C and DMA was removed under reduced pressure. The residue was purified by SiO_2 gel chromatography (eluent 100:0 CH_2Cl_2:MeOH to 95:5 CH_2Cl_2:MeOH) to afford the title compound. The obtained residue can be used for the next step without further purification or can be crystallized using a solvent mixture of MeCN/Et_2O. Characterization data for the resulting *N*-aryl-*N*-pyridinium amines (**3**) are collected below.

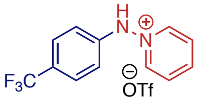

3a

1-((4-(trifluoromethyl)phenyl)amino)pyridin-1-ium triflate (***3a***). Prepared from (4-(trifluoromethyl)phenyl)boronic acid (**2a**) following General Procedure A and obtained as a pale-yellow solid (75.0 mg, 97% yield). ^{1}H NMR (δ, 23 °C, 400 MHz, CD_3CN): 9.69 (bs, 1H), 8.87 (dd, J = 6.3, 0.6 Hz, 2H), 8.69 (t, J = 7.9 Hz, 1H), 8.20 (t, J = 7.3 Hz, 2H), 7.67 (d, J = 9.0 Hz, 2H), 6.89 (d, J = 8.5 Hz, 2H). ^{13}C NMR (δ, 23 °C, 100 MHz, CD_3CN): 148.7, 148.6, 148.0, 131.0, 120.2 (q, $^{1}J_{C–F}$ = 318 Hz), 128.1, 128.0, 116.1. ^{19}F NMR (δ, 23 °C, 376 MHz, CD_3CN): –62.6, –79.4. HRMS (ESI) m/z: [M]$^+$ Calculated for $C_{12}H_{10}F_3N_2^+$ 239.0791, Found 239.0786.

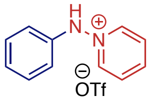

3b

1-(phenylamino)pyridin-1-ium triflate (***3b***). Prepared from phenylboronic acid (**2b**) following General Procedure A and obtained as a pale-yellow oil (51.8 mg, 81% yield). ^{1}H NMR (δ, 23 °C, 400 MHz, $CDCl_3$): 10.09 (bs, 1H), 8.87 (dd, J = 6.9, 1.3 Hz, 2H), 8.51 (t, J = 7.8 Hz, 1H), 8.10–8.06 (m, 2H), 7.34 (t, J = 8.0 Hz, 2H), 7.17 (t, J = 7.5 Hz, 1H), 6.89 (dd, J = 8.6, 1.1 Hz, 2H). ^{13}C NMR (δ, 23 °C, 100 MHz, $CDCl_3$): 145.6, 145.5, 143.6, 130.3, 129.2, 125.9, 120.2 (q, $^{1}J_{C–F}$ = 318 Hz), 118.1. ^{19}F NMR (δ, 23 °C, 376 MHz, $CDCl_3$): –78.4. HRMS (ESI) m/z: [M]$^+$ Calculated for $C_{11}H_{11}N_2^+$ 171.0917, Found 171.0915.

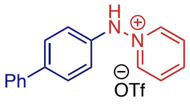

3c

1-([1,1'-biphenyl]-4-ylamino)pyridin-1-ium triflate (***3c***). Prepared from [1,1'-biphenyl]-4-ylboronic acid (**2c**) following General Procedure A and obtained as a white solid (48.3 mg, 61% yield). ^{1}H NMR (δ, 23 °C, 400 MHz, CD_3CN): 9.39 (bs, 1H), 8.89 (d, J = 5.4 Hz, 2H), 8.64 (t, J = 7.9 Hz, 1H), 8.16 (dd, J = 7.8, 6.9 Hz, 2H), 7.67–7.60 (m, 4H), 7.45 (t, J = 7.5 Hz, 2H), 7.37 (dt, J = 7.4, 1.6 Hz, 1H), 6.93 (d, J = 8.7 Hz, 2H). ^{13}C NMR (δ, 23 °C, 100 MHz, CD_3CN): 144.8, 140.7, 138.5, 130.7, 130.1, 129.4, 128.6, 127.8, 120.5 (q, $^{1}J_{C–F}$ = 318 Hz), 118.1. ^{19}F NMR (δ, 23 °C, 376 MHz, CD_3CN): –79.3. HRMS (ESI) m/z: [M]$^+$ Calculated for $C_{17}H_{15}N_2^+$ 247.1230, Found 247.1226.

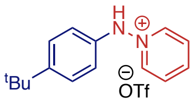

3d

1-((4-(tert-butyl)phenyl)amino)pyridin-1-ium triflate (***3d***). Prepared from (4-(*tert*-butyl)phenyl)boronic acid (**2d**) following General Procedure A and obtained as a pale-yellow solid (46.6 mg, 62% yield). ^{1}H NMR (δ, 23 °C, 400 MHz, CD_3CN): 9.18 (bs, 1H), 8.82 (d, J = 5.7 Hz, 2H), 8.59 (t, J = 7.8 Hz, 1H), 8.12 (t, J = 7.4 Hz, 2H), 7.42 (d, J = 8.8 Hz, 2H), 6.80 (d, J = 8.8 Hz, 2H), 1.28 (s, 9H). ^{13}C NMR (δ, 23 °C, 100 MHz, CD_3CN): 149.4, 147.5, 146.8, 142.8, 130.6,

127.9, 120.2 (q, $^{1}J_{C\text{–}F}$ = 318 Hz), 117.9, 35.2, 31.6. ^{19}F NMR (δ, 23 °C, 376 MHz, CD_3CN): –79.3. HRMS (ESI) m/z: $[M]^+$ Calculated for $C_{15}H_{19}N_2^+$ 227.1543, Found 227.1538.

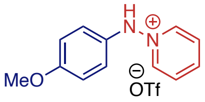

3e

*1-((4-methoxyphenyl)amino)pyridin-1-ium triflate (**3e**).* Prepared from (4-methoxyphenyl)boronic acid (**2e**) following General Procedure B and obtained as a brown oil (51.3 mg, 73% yield). ^{1}H NMR (δ, 23 °C, 400 MHz, CD_3CN): 8.89 (d, J = 6.9 Hz, 2H), 8.64 (t, J = 7.9 Hz, 1H), 8.16 (t, J = 7.3 Hz, 2H), 7.64 (d, J = 9.2 Hz, 2H), 7.21 (d, J = 9.2 Hz, 2H), 3.91 (s, 3H). ^{13}C NMR (δ, 23 °C, 100 MHz, CD_3CN): 162.9, 147.2, 145.6, 129.3, 126.8, 120.2 (q, $^{1}J_{C\text{–}F}$ = 318 Hz), 116.4, 56.7. ^{19}F NMR (δ, 23 °C, 376 MHz, CD_3CN): –79.3. HRMS (APCI) m/z: $[M]^+$ Calculated for $C_{12}H_{13}N_2O^+$ 201.1022, Found 201.1018.

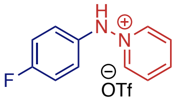

3f

*1-((4-fluorophenyl)amino)pyridin-1-ium triflate (**3f**).* Prepared from (4-fluorophenyl)boronic acid (**2f**) following General Procedure A and obtained as a white solid (54.1 mg, 80% yield). ^{1}H NMR (δ, 23 °C, 400 MHz, CD_3CN): 9.34 (bs, 1H), 8.84 (d, J = 5.4 Hz, 2H), 8.59 (t, J = 7.8 Hz, 1H), 8.12 (t, J = 7.3 Hz, 2H), 7.15 (t, J = 8.8 Hz, 2H), 6.97 (t, J = 4.5 Hz, 2H). ^{13}C NMR (δ, 23 °C, 100 MHz, CD_3CN): 159.9 (d, $^{1}J_{C\text{–}F}$ = 241 Hz), 147.4, 146.5, 141.2, 130.6, 120.2 (q, $^{1}J_{C\text{–}F}$ = 318 Hz), 121.03 (d, $^{3}J_{C\text{–}F}$ = 9 Hz), 117.5 (d, $^{2}J_{C\text{–}F}$ = 23 Hz). ^{19}F NMR (δ, 23 °C, 376 MHz, CD_3CN): –79.3, –119.1. HRMS (ESI) m/z: $[M]^+$ Calculated for $C_{11}H_{10}FN_2^+$ 189.0823, Found 189.0819.

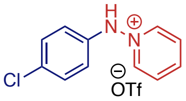

3g

*1-((4-chlorophenyl)amino)pyridin-1-ium triflate (**3g**).* Prepared from (4-chlorophenyl)boronic acid (**2g**) following General Procedure A and obtained as a white solid (45.3 mg, 64% yield). ^{1}H NMR (δ, 23 °C, 400 MHz, CD_3CN): 9.24 (bs, 1H), 8.83 (d, J = 6.7 Hz, 2H), 8.63 (t, J = 7.9 Hz, 1H), 8.14 (t, J = 7.4 Hz, 2H), 7.38 (d, J = 9.0 Hz, 2H), 6.84 (d, J = 8.9 Hz, 2H). ^{13}C NMR (δ, 23 °C, 100 MHz, CD_3CN): 147.7, 147.0, 143.9, 130.5, 130.5, 130.3, 120.2 (q, $^{1}J_{C\text{–}F}$ = 318 Hz), 119.0. ^{19}F NMR (δ, 23 °C, 376 MHz, CD_3CN): –79.4. HRMS (ESI) m/z: $[M]^+$ Calculated for $C_{11}H_{10}ClN_2^+$ 207.0527, Found 207.0525.

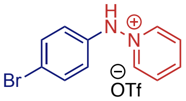

3h

*1-((4-bromophenyl)amino)pyridin-1-ium triflate (**3h**).* Prepared from (4-bromophenyl)boronic acid (**2h**) following General Procedure A and obtained as a pale-brown solid (78.2 mg, 98% yield). ^{1}H NMR (δ, 23 °C, 400 MHz, CD_3CN): 9.49 (bs, 1H), 8.85 (d, J = 6.0 Hz, 2H), 8.64 (t, J = 7.8 Hz, 1H), 8.15 (t, J = 7.2 Hz, 2H), 7.51 (d, J = 8.8 Hz, 2H), 6.78 (d, J = 8.8 Hz, 2H). ^{13}C NMR (δ, 23 °C, 100 MHz, CD_3CN): 147.9, 147.2, 144.7, 133.7, 130.7, 120.2 (q, $^{1}J_{C\text{–}F}$ = 318 Hz), 119.3,

117.8. ^{19}F NMR (δ, 23 °C, 376 MHz, CD_3CN): –79.3. HRMS (ESI) m/z: $[M]^+$ Calculated for $C_{11}H_{10}BrN_2^+$ 249.0022, Found 249.0018.

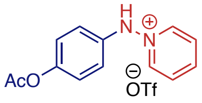

3i

*1-((4-acetoxyphenyl)amino)pyridin-1-ium triflate (**3i**).* Prepared from (4-acetoxyphenyl)boronic acid (**2i**) following General Procedure A and obtained as a yellow oil (38.5 mg, 51% yield). ^{1}H NMR (δ, 23 °C, 400 MHz, CD_3CN): 9.17 (bs, 1H), 8.84 (d, J = 5.6 Hz, 2H), 8.61 (t, J = 7.9 Hz, 1H), 8.14 (t, J = 7.4 Hz, 2H), 7.11 (d, J = 9.1 Hz, 2H), 6.89 (d, J = 9.1 Hz, 2H), 2.23 (s, 3H). ^{13}C NMR (δ, 23 °C, 100 MHz, CD_3CN): 170.8, 149.1, 147.9, 147.1, 142.8, 130.8, 124.5, 120.2 (q, $^1J_{C\text{–}F}$ = 318 Hz), 119.2, 21.4. ^{19}F NMR (δ, 23 °C, 376 MHz, CD_3CN): –79.3.

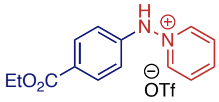

3j

*1-((4-(ethoxycarbonyl)phenyl)amino)pyridin-1-ium triflate (**3j**).* Prepared from (4-(ethoxycarbonyl)phenyl)boronic acid (**2j**) following General Procedure B and obtained as a white solid (53.2 mg, 68% yield). ^{1}H NMR (δ, 23 °C, 400 MHz, CD_3CN): 9.77 (bs, 1H), 8.87 (d, J = 6.6 Hz, 2H), 8.69 (t, J = 7.9 Hz, 1H), 8.20 (t, J = 7.4 Hz, 2H), 7.97 (d, J = 8.8 Hz, 2H), 6.81 (d, J = 8.9 Hz, 2H), 4.30 (q, J = 7.1 Hz, 2H), 1.33 (t, J = 7.1 Hz, 3H). ^{13}C NMR (δ, 23 °C, 100 MHz, CD_3CN): 166.5, 149.4, 148.5, 148.0, 132.2, 131.0, 127.1, 120.2 (q, $^1J_{C\text{–}F}$ = 318 Hz), 115.6, 61.9, 14.6. ^{19}F NMR (δ, 23 °C, 376 MHz, CD_3CN): –79.3. HRMS (ESI) m/z: $[M]^+$ Calculated for $C_{14}H_{15}N_2O_2^+$ 243.1128, Found 243.1124.

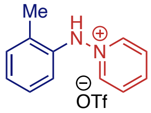

3k

*1-(o-tolylamino)pyridin-1-ium trifluoromethanesulfonate (**3k**).* Prepared from *o*-tolylboronic acid (**2k**) following General Procedure B and obtained as a yellow oil (28.0 mg, 42% yield). ^{1}H NMR (δ, 23 °C, 400 MHz, CD_3CN): 8.96 (bs, 1H), 8.78–8.76 (m, 2H), 8.58 (dd, J = 8.4, 7.2 Hz, 1H), 8.12 (dd, J = 7.8, 7.0 Hz, 2H), 7.34 (dt, J = 6.3, 1.0 Hz, 1H), 7.22–7.18 (m, 2H), 6.56–6.54 (m, 1H), 2.28 (s, 3H). ^{13}C NMR (δ, 23 °C, 100 MHz, CD_3CN): 146.5, 145.4, 142.1, 132.4, 130.2, 128.3, 126.6, 120.2 (q, $^1J_{C\text{–}F}$ = 318 Hz), 17.2. ^{19}F NMR (δ, 23 °C, 376 MHz, CD_3CN): –79.3. HRMS (ESI) m/z: $[M]^+$ Calculated for $C_{12}H_{13}N_2^+$ 185.1073, Found 185.1071.

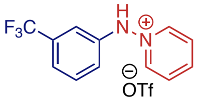

3l

*1-((3-(trifluoromethyl)phenyl)amino)pyridin-1-ium triflate (**3l**).* Prepared from (3-(trifluoromethyl)phenyl)boronic acid (**2l**) following General Procedure A and obtained as a white solid (60.5 mg, 78% yield). ^{1}H NMR (δ, 23 °C, 400 MHz, CD_3CN): 9.55 (bs, 1H), 8.86 (d, J = 5.1 Hz, 2H), 8.66 (t, J = 7.9 Hz, 1H), 8.17 (t, J = 7.4 Hz, 2H), 7.55 (t, J = 8.0 Hz, 1H), 7.46 (dt, J = 7.8, 0.7 Hz, 1H), 7.14 (s, 1H), 7.04 (d, J = 8.1 Hz, 1H). ^{13}C NMR (δ, 23 °C, 100 MHz, CD_3CN):

148.3, 147.5, 145.9, 131.9, 130.8, 123.5, 121.9, 120.5 (q, $^1J_{C\text{–}F}$ = 318 Hz), 113.7. ^{19}F NMR (*δ*, 23 °C, 376 MHz, CD_3CN): –78.3, –63.4. HRMS (ESI) m/z: $[M]^+$ Calculated for $C_{12}H_{10}F_3N_2^+$ 239.0791, Found 239.0786.

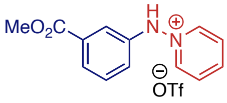

3m

1-((3-(methoxycarbonyl)phenyl)amino)pyridin-1-ium triflate ***(3m)***. Prepared from (3-(methoxycarbonyl)phenyl)boronic acid (**2m**) following General Procedure A and obtained as a white solid (44.5 mg, 59% yield). ^{1}H NMR (*δ*, 23 °C, 400 MHz, CD_3CN): 9.43 (bs, 1H), 8.87 (d, *J* = 6.5 Hz, 2H), 8.66 (t, *J* = 8.2 Hz, 1H), 8.17 (t, *J* = 7.1 Hz, 2H), 7.79 (d, *J* = 7.8 Hz, 1H), 7.50 (t, *J* = 7.9 Hz, 1H), 7.44 (s, 1H), 7.06 (d, *J* = 10.6 Hz, 1H), 3.86 (s, 3H). ^{13}C NMR (*δ*, 23 °C, 100 MHz, CD_3CN): 166.9, 148.1, 147.4, 145.5, 133.0, 131.3, 130.7, 126.4, 121.7, 120.2 (q, $^1J_{C\text{–}F}$ = 318 Hz), 117.8, 53.0. ^{19}F NMR (*δ*, 23 °C, 376 MHz, CD_3CN): –79.4. HRMS (ESI) m/z: $[M]^+$ Calculated for $C_{13}H_{13}N_2O_2^+$ 229.0972, Found 229.0968.

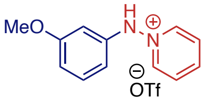

3n

1-((3-methoxyphenyl)amino)pyridin-1-ium triflate ***(3n)***. Prepared from (3-methoxyphenyl)boronic acid (**2n**) following General Procedure A and obtained as a white solid (58.1 mg, 83% yield). ^{1}H NMR (*δ*, 23 °C, 400 MHz, CD_3CN): 9.22 (bs, 1H), 8.84 (d, *J* = 7.0 Hz, 2H), 8.62 (t, *J* = 7.9 Hz, 1H), 8.14 (dd, *J* = 7.8, 7.0 Hz, 2H), 7.29 (t, *J* = 8.2 Hz, 1H), 6.75 (ddd, *J* = 8.4, 2.4, 0.8 Hz, 1H), 6.42–6.37 (m, 2H), 3.76 (s, 3H). ^{13}C NMR (*δ*, 23 °C, 100 MHz, CD_3CN): 162.0, 147.7, 147.2, 146.6, 131.9, 130.5, 120.2 (q, $^1J_{C\text{–}F}$ = 318 Hz), 111.1, 109.5, 103.7, 56.2. ^{19}F NMR (*δ*, 23 °C, 376 MHz, CD_3CN): –79.3. HRMS (ESI) m/z: $[M]^+$ Calculated for $C_{12}H_{13}N_2O^+$ 201.1022, Found 201.1019.

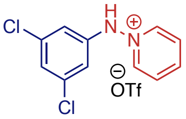

3o

1-((3,5-dichlorophenyl)amino)pyridin-1-ium triflate ***(3o)***. Prepared from (3,5-dichlorophenyl)boronic acid (**2o**) following General Procedure B and obtained as a white solid (21.7 mg, 28% yield). ^{1}H NMR (*δ*, 23 °C, 400 MHz, CD_3CN): 9.55 (bs, 1H), 8.84 (d, *J* = 6.4 Hz, 2H), 8.67 (t, *J* = 7.9 Hz, 1H), 8.18 (t, *J* = 7.1 Hz, 2H), 7.23 (s, 1H), 6.80 (s, 2H). ^{13}C NMR (*δ*, 23 °C, 100 MHz, CD_3CN): 148.6, 147.8, 147.4, 136.9, 131.0, 124.9, 120.2 (q, $^1J_{C\text{–}F}$ = 318 Hz), 115.3. ^{19}F NMR (*δ*, 23 °C, 376 MHz, CD_3CN): –79.3. HRMS (ESI) m/z: $[M]^+$ Calculated for $C_{11}H_9Cl_2N_2^+$ 239.0137, Found 239.0135.

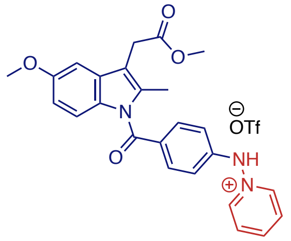

3p

*1-((4-(5-methoxy-3-(2-methoxy-2-oxoethyl)-2-methyl-1H-indole-1-carbonyl)phenyl)amino)-pyridin-1-ium triflate (**3p**)*. Prepared from (4-(5-methoxy-3-(2-methoxy-2-oxoethyl)-2-methyl-1H-indole-1-carbonyl)phenyl)boronic acid (**2p**) following General Procedure A and obtained as a yellow solid (37.1 mg, 32% yield). ^{1}H NMR (δ, 23 °C, 400 MHz, CD_3CN): 8.89 (d, J = 5.9 Hz, 2H), 8.67 (t, J = 7.8 Hz, 1H), 8.19 (t, J = 7.1 Hz, 2H), 7.65 (d, J = 8.6 Hz, 2H), 6.97 (d, J = 8.8 Hz, 2H), 6.83 (d, J = 8.6 Hz, 2H), 6.68 (dd, J = 9.0, 2.4 Hz, 1H), 3.79 (s, 3H), 3.71 (s, 2H), 3.65 (s, 3H), 2.27 (s, 3H). ^{13}C NMR (δ, 23 °C, 100 MHz, CD_3CN): 172.3, 169.3, 156.9, 149.7, 148.3, 147.9, 136.9, 132.8, 131.8, 131.7, 131.5, 130.9, 120.2 (q, $^1J_{C\text{–}F}$ = 318 Hz), 115.8, 115.5, 113.4, 112.3, 102.2, 56.2, 52.5, 30.2, 13.5. ^{19}F NMR (δ, 23 °C, 376 MHz, CD_3CN): –79.3. HRMS (APCI) m/z: $[M]^+$ Calculated for $C_{25}H_{24}N_3O_4^+$ 430.1761, Found 430.1751.

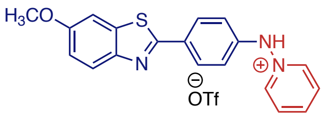

3q

*1-((4-(6-methoxybenzo[d]thiazol-2-yl)phenyl)amino)pyridin-1-ium triflate (**3q**)*. Prepared from (4-(6-methoxybenzo[d]thiazol-2-yl)phenyl)boronic acid (**2q**) following General Procedure A and obtained as a pale-brown solid (44.5 mg, 46% yield). ^{1}H NMR (δ, 23 °C, 400 MHz, CD_3CN): 9.56 (bs, 1H), 8.90 (d, J = 5.6 Hz, 2H), 8.68 (t, J = 7.9 Hz, 1H), 8.19 (t, J = 7.3 Hz, 2H), 8.02 (d, J = 8.9 Hz, 2H), 7.86 (d, J = 9.0 Hz, 1H), 7.52 (d, J = 2.5 Hz, 1H), 7.11 (dd, J = 9.0, 2.6 Hz, 1H), 6.90 (d, J = 8.8 Hz, 2H), 3.86 (s, 3H). ^{13}C NMR (δ, 23 °C, 100 MHz, CD_3CN): 165.1, 159.0, 149.5, 148.3, 147.7, 147.4, 137.4, 130.8, 130.7, 129.5, 124.4, 120.2 (q, $^1J_{C\text{–}F}$ = 318 Hz), 117.0, 116.9, 105.5, 56.6. ^{19}F NMR (δ, 23 °C, 376 MHz, CD_3CN): –79.3. HRMS (ESI) m/z: $[M]^+$ Calculated for $C_{19}H_{16}N_3OS^+$ 334.1009, Found 334.1007.

B.1.1 Gram-Scale Synthesis of 3a

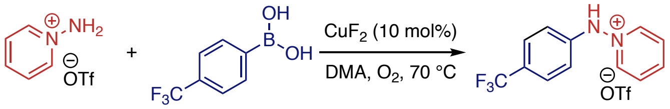

A 25-mL round bottom flask was charged with *N*-aminopyridinium triflate **1** (1.95 g, 8.00 mmol, 1.00 equiv), (4-(trifluoromethyl)phenyl)boronic acid (**2a**, 1.52 g, 8.00 mmol, 1.00 equiv), copper(II) fluoride (40.6 mg, 0.400 mmol, 0.050 equiv), and *N,N*-dimethylacetamide (DMA, 10.0 mL). A magnetic stir bar was added, and the flask was fitted with a rubber septum. Oxygen was bubbled through the reaction mixture for 1 h at 23 °C. Under an O_2 atmosphere, the mixture was heated at 70 °C in an oil bath for 19 h. After this time, the reaction mixture was cooled to 23 °C and O_2 was again bubbled through the reaction mixture for 1 h at 23 °C. (4-(Trifluoromethyl)phenyl)boronic acid (**2a**, 1.52 g, 8.00 mmol, 1.00 equiv), copper(II) fluoride (40.6 mg, 0.400 mmol, 0.050 equiv) and DMA (10.0 mL) were added. Under an O_2 atmosphere, the mixture was heated at 70 °C in an oil bath for an additional 19 h. The reaction mixture was cooled to 23 °C and DMA was removed under reduced pressure. The residue was purified by SiO_2 gel chromatography (eluent 100:0 CH_2Cl_2:MeOH to 95:5 CH_2Cl_2:MeOH) to afford the title compound. The obtained residue was further crystallized using a solvent mixture of MeCN/Et_2O to yield **3a** as a pale-yellow solid (2.20 g, 71% yield).

B.2 Alkylation of the Synthesized *N*-Aminopyridinium Derivatives

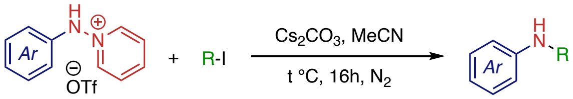

A 20-mL scintillation vial was charged with *N*-aminopyridinium derivative (**3**, 0.10 mmol, 1.0 equiv), cesium carbonate (98 mg, 0.30 mmol, 3.0 equiv), and a magnetic stir bar in an N_2-filled dry box. The appropriate alkyl iodide (**4**, 0.20 mmol, 2.0 equiv) and acetonitrile (1.5 mL) was added to the reaction vessel. The reaction vial was sealed under N_2 atmosphere and heated at the specified temperatures inside a reaction block for 16 h. After cooling to 23 °C, the reaction mixture was concentrated under reduced pressure. The residue was purified by SiO_2 gel chromatography (eluent 100:0 hexanes:EtOAc to 95:5 hexanes:EtOAc) to afford the title compound **5**.

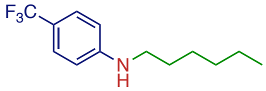

5a

N-*hexyl-4-(trifluoromethyl)aniline* (**5a**). Prepared from 1-((4-(trifluoromethyl)phenyl)amino)pyridin-1-ium triflate (**3a**) and 1-iodohexane at 70 °C and obtained as a colorless oil (18.6 mg, 76% yield). ^{1}H NMR (δ, 23 °C, 400 MHz, $CDCl_3$): 7.39 (d, J = 8.5 Hz, 2H), 6.58 (d, J = 8.5 Hz, 2H), 3.94 (bs, 1H), 3.13 (q, J = 6.3 Hz, 2H), 1.62 (dt, J = 14.8, 7.3 Hz, 2H), 1.42–1.30 (m, 6H), 0.90 (t, J = 6.9 Hz, 3H). The obtained spectral data are in good agreement with those reported in literature.[14] With hexyl trifluoromethanesulfonate and 1-bromohexane as the alkylating agents, **5a** was obtained in 63% and 49% yield, respectively.

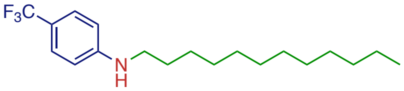

5b

N-*dodecyl-4-(trifluoromethyl)aniline* (**5b**). Prepared from 1-((4-(trifluoromethyl)phenyl)amino)-pyridin-1-ium triflate (**3a**) and 1-iodododecane at 70 °C and obtained as a colorless oil (26.0 mg, 79% yield). ^{1}H NMR (δ, 23 °C, 400 MHz, $CDCl_3$): 7.39 (d, J = 8.6 Hz, 2H), 6.58 (d, J = 8.6 Hz, 2H), 3.94 (bs, 1H), 3.13 (t, J = 7.0 Hz, 2H), 1.62 (dt, J = 14.7, 7.3 Hz, 2H), 1.27 (bs, 15H), 0.90–0.83 (m, 6H). ^{13}C NMR (δ, 23 °C, 100 MHz, $CDCl_3$): 151.0, 126.7, 123.8 (q, $^1J_{C\text{–}F}$ = 269 Hz), 118.4 (q, $^2J_{C\text{–}F}$ = 32 Hz), 111.8, 43.7, 32.1, 29.9, 29.8, 29.8, 29.7, 29.5, 29.5, 29.5, 27.2, 22.8, 14.3. ^{19}F NMR (δ, 23 °C, 376 MHz, $CDCl_3$): –60.9. HRMS (APCI) m/z: [M+H]+ Calculated for $C_{19}H_{31}F_3N$ 330.2403, Found 330.2395.

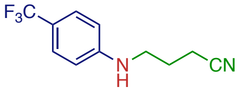

5c

4-((4-(trifluoromethyl)phenyl)amino)butanenitrile (**5c**). Prepared from 1-((4-(trifluoromethyl)-phenyl)amino)pyridin-1-ium triflate (**3a**) and 4-iodobutanenitrile (**4c**) at 70 °C and obtained as a pale-yellow oil (18.0 mg, 79% yield). ^{1}H NMR (δ, 23 °C, 400 MHz, $CDCl_3$): 7.43 (d, J = 6.9 Hz, 2H), 6.65 (d, J = 8.9 Hz, 2H), 4.31 (bs, 1H), 3.37 (t, J = 6.7 Hz, 2H), 2.48 (t, J = 7.0 Hz, 2H), 1.99

(t, J = 6.8 Hz, 2H). The obtained spectral data are in good agreement with those reported in literature.[15]

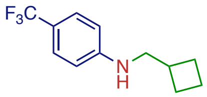

5d

N-*(cyclobutylmethyl)-4-(trifluoromethyl)aniline* ***(5d)***. Prepared from 1-((4-(trifluoromethyl)-phenyl)amino)pyridin-1-ium triflate (**3a**) and (iodomethyl)cyclobutane (**4d**) at 70 °C and obtained as a pale-yellow oil (11.9 mg, 52% yield). ^{1}H NMR (δ, 23 °C, 400 MHz, $CDCl_3$): 7.39 (d, J = 8.4 Hz, 2H), 6.62 (d, J = 8.5 Hz, 2H), 3.16 (d, J = 7.3 Hz, 2H), 2.60 (dt, J = 15.2, 7.6 Hz, 1H), 2.15–2.09 (m, 2H), 2.00–1.87 (m, 2H), 1.79–1.70 (m, 2H). ^{13}C NMR (δ, 23 °C, 100 MHz, $CDCl_3$): 150.7, 126.7, 123.8 (q, $^1J_{C\text{–}F}$ = 269 Hz), 118.4 (q, $^2J_{C\text{–}F}$ = 32 Hz), 112.2, 49.6, 34.8, 26.2, 18.6. ^{19}F NMR (δ, 23 °C, 376 MHz, $CDCl_3$): –61.0. HRMS (APCI) m/z: [M+H]$^+$ Calculated for $C_{12}H_{15}F_3N$ 230.1151, Found 230.1146.

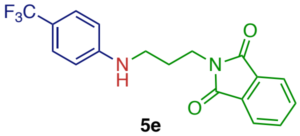

5e

2-(3-((4-(trifluoromethyl)phenyl)amino)propyl)isoindoline-1,3-dione ***(5e)***. Prepared from 1-((4-(trifluoromethyl)phenyl)amino)pyridin-1-ium triflate (**3a**) and 2-(3-iodopropyl)isoindoline-1,3-dione (**4e**) at 70 °C and obtained as a white solid (25.7 mg, 74% yield). ^{1}H NMR (δ, 23 °C, 400 MHz, $CDCl_3$): 7.85 (dd, J = 5.4, 3.1 Hz, 2H), 7.73 (dd, J = 5.4, 3.1 Hz, 2H), 7.38 (d, J = 8.5 Hz, 2H), 6.62 (d, J = 8.5 Hz, 2H), 4.56 (bs, 1H), 3.81 (t, J = 6.5 Hz, 2H), 3.22 (t, J = 6.4 Hz, 2H), 1.98 (quintet, J = 6.5 Hz, 2H). ^{13}C NMR (δ, 23 °C, 100 MHz, $CDCl_3$): 168.8, 150.3, 134.3, 132.1, 126.8, 126.8, 126.8, 126.7, 123.8, 123.5 (q, $^1J_{C\text{–}F}$ = 269 Hz), 123.5, 118.7 (q, $^2J_{C\text{–}F}$ = 32 Hz), 112.1, 40.3, 35.3, 27.7. ^{19}F NMR (δ, 23 °C, 376 MHz, $CDCl_3$): –61.0. HRMS (APCI) m/z: [M+H]$^+$ Calculated for $C_{18}H_{16}F_3N_2O_2$ 349.1158, Found 349.1150.

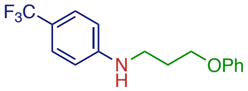

5f

N-*(3-phenoxypropyl)-4-(trifluoromethyl)aniline* ***(5f)***. Prepared from 1-((4-(trifluoromethyl)-phenyl)amino)pyridin-1-ium triflate (**3a**) and (3-iodopropoxy)benzene (**4f**) at 70 °C and obtained as a pale-yellow solid (17.1 mg, 58% yield). ^{1}H NMR (δ, 23 °C, 400 MHz, $CDCl_3$): 7.40 (d, J = 8.4 Hz, 2H), 7.30 (dd, J = 8.7, 7.4 Hz, 2H), 6.97 (t, J = 7.4 Hz, 1H), 6.91 (d, J = 7.7 Hz, 2H), 6.65 (d, J = 8.5 Hz, 2H), 4.67 (bs, 1H), 4.10 (t, J = 5.7 Hz, 2H), 3.41 (t, J = 6.7 Hz, 2H), 2.12 (quintet, J = 6.0 Hz, 2H). ^{13}C NMR (δ, 23 °C, 100 MHz, $CDCl_3$): 158.8, 150.5, 129.7, 126.8, 123.7 (q, $^1J_{C\text{–}F}$ = 269 Hz), 121.2, 118.7 (q, $^2J_{C\text{–}F}$ = 32 Hz), 114.6, 112.2, 65.9, 41.4, 28.8. ^{19}F NMR (δ, 23 °C, 376 MHz, $CDCl_3$): –61.0. HRMS (APCI) m/z: [M+H]$^+$ Calculated for $C_{16}H_{17}F_3NO$ 296.1257, Found 296.1249.

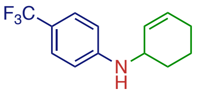

5g

N-*(cyclohex-2-en-1-yl)-4-(trifluoromethyl)aniline (**5g**)*. Prepared from 1-((4-(trifluoromethyl)-phenyl)amino)pyridin-1-ium triflate (**3a**) and 3-iodocyclohex-1-ene (**4g**) at 70 °C and obtained as a colorless oil (19.5 mg, 81% yield). ^{1}H NMR (δ, 23 °C, 400 MHz, $CDCl_3$): 7.39 (d, J = 8.4 Hz, 2H), 6.61 (d, J = 8.4 Hz, 2H), 5.91–5.87 (m, 1H), 5.73–5.70 (m, 1H), 4.02 (bs, 2H), 2.05 (m, 2H), 1.92–1.63 (m, 4H). The obtained spectral data are in good agreement with those reported in literature.[16]

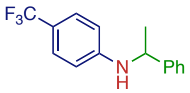

5h

N-*(1-phenylethyl)-4-(trifluoromethyl)aniline (**5h**)*. Prepared from 1-((4-(trifluoromethyl)phenyl)-amino)pyridin-1-ium triflate (**3a**) and (1-iodoethyl)benzene (**4h**) at 70 °C and obtained as a pale-yellow oil (16.4 mg, 62% yield). ^{1}H NMR (δ, 23 °C, 400 MHz, $CDCl_3$): 7.33 (m, 6H), 7.27–7.23 (m, 1H), 6.52 (d, J = 8.4 Hz, 2H), 4.52 (q, J = 6.7 Hz, 1H), 4.38 (bs, 1H), 1.55 (d, J = 6.6 Hz, 3H). The obtained spectral data are in good agreement with those reported in literature.[17]

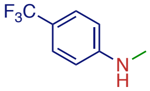

5j

N-*methyl-4-(trifluoromethyl)aniline (**5j**)*. Prepared from 1-((4-(trifluoromethyl)phenyl)amino)-pyridin-1-ium triflate (**3a**) and iodomethane at 25 °C and obtained as a pale-yellow oil (16.8 mg, 96% yield). ^{1}H NMR (δ, 23 °C, 400 MHz, $CDCl_3$): 7.44 (d, J = 8.2 Hz, 2H), 6.70 (d, J = 8.2 Hz, 2H), 2.89 (s, 3H). The obtained spectral data are in good agreement with those reported in literature.[18]

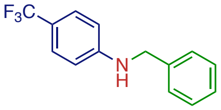

5k

N-*benzyl-4-(trifluoromethyl)aniline (**5k**)*. Prepared from 1-((4-(trifluoromethyl)phenyl)amino)-pyridin-1-ium triflate (**3a**) and (iodomethyl)benzene (**4k**) at 25 °C and obtained as a pale-yellow oil (24.6 mg, 98% yield). ^{1}H NMR (δ, 23 °C, 400 MHz, $CDCl_3$): 7.40 (d, J = 8.4 Hz, 2H), 7.37–7.33 (m, 4H), 7.33–7.28 (m, 1H), 6.64 (d, J = 8.4 Hz, 2H), 4.37 (s, 2H). The obtained spectral data are in good agreement with those reported in literature.[19]

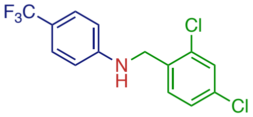

5l

N-*(2,4-dichlorobenzyl)-4-(trifluoromethyl)aniline (**5l**)*. Prepared from 1-((4-(trifluoromethyl)-phenyl)amino)pyridin-1-ium triflate (**3a**) and 2,4-dichloro-1-(iodomethyl)benzene (**4l**) at 25 °C

and obtained as a pale-yellow oil (19.1 mg, 60% yield). ^{1}H NMR (δ, 23 °C, 400 MHz, $CDCl_3$): 7.42–7.39 (m, 3H), 7.30 (d, J = 8.3 Hz, 1H), 7.20 (dd, J = 8.3, 2.1 Hz, 1H), 6.61 (d, J = 8.5 Hz, 2H), 4.44 (s, 2H). ^{13}C NMR (δ, 23 °C, 100 MHz, $CDCl_3$): 134.0, 129.7, 129.7, 127.5, 126.9, 126.9, 126.8, 123.6 (q, $^{1}J_{C–F}$ = 269 Hz), 112.4, 45.3. ^{19}F NMR (δ, 23 °C, 376 MHz, $CDCl_3$): –61.2. HRMS (APCI) m/z: [M+H]$^+$ Calculated for $C_{14}H_{11}Cl_2F_3N$ 320.0215, Found 320.0210.

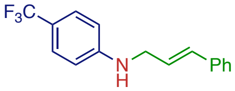

5m

N-*cinnamyl-4-(trifluoromethyl)aniline* ***(5m)***. Prepared from 1-((4-(trifluoromethyl)phenyl)-amino)pyridin-1-ium triflate (**3a**) and (*E*)-(3-iodoprop-1-en-1-yl)benzene (**4m**) at 25 °C and obtained as a white solid (11.9 mg, 43% yield). ^{1}H NMR (δ, 23 °C, 400 MHz, $CDCl_3$): 7.42 (d, J = 8.4 Hz, 2H), 7.38–7.29 (m, 4H), 7.24–7.22 (m, 1H), 6.71 (d, J = 8.6 Hz, 2H), 6.62 (d, J = 15.9 Hz, 1H), 6.29 (dt, J = 15.9, 5.8 Hz, 1H), 3.98 (dd, J = 5.8, 1.6 Hz, 2H). The obtained spectral data are in good agreement with those reported in literature.[20]

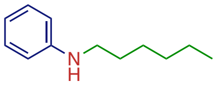

5n

N-*hexylaniline* ***(5n)***. Prepared from 1-(phenylamino)pyridin-1-ium triflate (**3b**) and 1-iodohexane at 50 °C and obtained as a pale-yellow oil (12.0 mg, 68% yield). ^{1}H NMR (δ, 23 °C, 400 MHz, $CDCl_3$): 7.19 (t, J = 8.0 Hz, 2H), 6.73 (t, J = 7.3 Hz, 1H), 6.68 (d, J = 7.6 Hz, 2H), 3.11 (t, J = 7.2 Hz, 2H), 1.63 (dt, J = 14.8, 7.3 Hz, 2H), 1.41–1.29 (m, 6H), 0.89 (t, J = 6.9 Hz, 3H). The obtained spectral data are in good agreement with those reported in literature.[21]

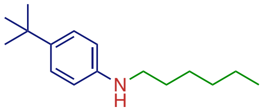

5o

4-(tert-butyl)-N-*hexylaniline* ***(5o)***. Prepared from 1-((4-(*tert*-butyl)phenyl)amino)pyridin-1-ium triflate (**3d**) and 1-iodohexane at 40 °C and obtained as a pale-yellow oil (12.8 mg, 55% yield). ^{1}H NMR (δ, 23 °C, 400 MHz, $CDCl_3$): 7.22 (d, J = 8.7 Hz, 2H), 6.63 (d, J = 8.7 Hz, 2H), 3.10 (t, J = 7.2 Hz, 2H), 1.61 (q, J = 7.4 Hz, 2H), 1.40–1.30 (m, 6H), 1.28 (s, 9H), 0.89 (t, J = 7.0 Hz, 3H). The obtained spectral data are in good agreement with those reported in literature.[22]

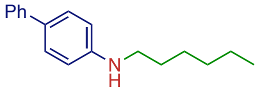

5p

N-*hexyl-[1,1'-biphenyl]-4-amine* ***(5p)***. Prepared from 1-([1,1'-biphenyl]-4-ylamino)pyridin-1-ium triflate (**3c**) and 1-iodohexane at 45 °C and obtained as a pale-yellow solid (14.4 mg, 57% yield). ^{1}H NMR (δ, 23 °C, 400 MHz, $CDCl_3$): 7.55–7.52 (m, 2H), 7.46–7.44 (m, 2H), 7.39 (t, J = 7.7 Hz, 2H), 7.25 (t, J = 7.4 Hz, 1H), 6.73 (d, J = 8.6 Hz, 2H), 3.16 (t, J = 7.2 Hz, 2H), 1.65 (q, J = 7.4 Hz, 2H), 1.43–1.31 (m, 6H), 0.92–0.89 (m, 3H). The obtained spectral data are in good agreement with those reported in literature.[23]

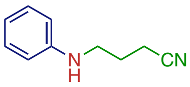

5q

*4-(phenylamino)butanenitrile (**5q**).* Prepared from 1-(phenylamino)pyridin-1-ium triflate (**3b**) and 4-iodobutanenitrile (**4c**) at 50 °C and obtained as a pale-yellow oil (14.1 mg, 88% yield). ^{1}H NMR (δ, 23 °C, 400 MHz, $CDCl_3$): 7.21 (t, J = 8.0 Hz, 2H), 6.77 (dd, J = 7.8, 6.9 Hz, 1H), 6.66 (d, J = 7.6 Hz, 2H), 3.33 (t, J = 6.7 Hz, 2H), 2.48 (t, J = 7.1 Hz, 2H), 1.99 (quintet, J = 6.9 Hz, 2H). The obtained spectral data are in good agreement with those reported in literature.[15]

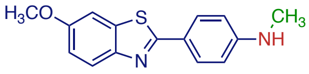

5r
thioflavin T derived

*4-(6-methoxybenzo[d]thiazol-2-yl)-N-methylaniline (**5r**).* Prepared from 1-((4-(6-methoxybenzo[d]thiazol-2-yl)phenyl)amino)pyridin-1-ium triflate (**3q**) and iodomethane at 50 °C and obtained as a yellow solid (20.0 mg, 74% yield). ^{1}H NMR (δ, 23 °C, 400 MHz, $CDCl_3$): 7.90 (d, J = 8.7 Hz, 3H), 7.31 (d, J = 2.5 Hz, 1H), 7.05 (dd, J = 8.9, 2.6 Hz, 1H), 6.65 (d, J = 8.8 Hz, 2H), 3.88 (s, 3H), 2.91 (s, 3H). The obtained spectral data are in good agreement with those reported in literature.[24]

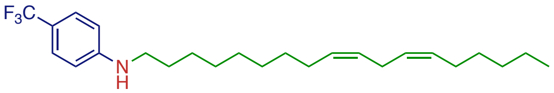

5s
linoleyl Iodide derived

N-*((9Z,12Z)-octadeca-9,12-dien-1-yl)-4-(trifluoromethyl)aniline (**5s**).* Prepared from 1-((4-(trifluoromethyl)phenyl)amino)pyridin-1-ium triflate (**3a**) and (6*Z*,9*Z*)-18-iodooctadeca-6,9-diene (**4s**) at 70 °C and obtained as a yellow oil (28.2 mg, 69% yield). ^{1}H NMR (δ, 23 °C, 400 MHz, $CDCl_3$): 7.39 (d, J = 8.4 Hz, 2H), 6.59 (d, J = 8.5 Hz, 2H), 5.40–5.30 (m, 5H), 3.13 (t, J = 7.1 Hz, 2H), 2.78 (t, J = 6.5 Hz, 2H), 2.05 (q, J = 7.0 Hz, 4H), 1.66–1.59 (m, 2H), 1.37–1.28 (m, 15H), 0.89 (t, J = 6.9 Hz, 4H). ^{13}C NMR (δ, 23 °C, 100 MHz, $CDCl_3$): 150.9, 130.4, 130.2, 128.2, 128.05, 126.7, 123.8 (q, $^1J_{C\text{–}F}$ = 269 Hz), 118.7 (q, $^2J_{C\text{–}F}$ = 32 Hz), 111.8, 43.7, 31.7, 29.8, 29.6, 29.5, 29.4, 29.4, 27.4, 27.2, 25.8, 22.7, 14.2. ^{19}F NMR (δ, 23 °C, 376 MHz, $CDCl_3$): –61.0. HRMS (APCI) m/z: $[M+H]^+$ Calculated for $C_{25}H_{39}F_3N$ 410.3029, Found 410.3020.

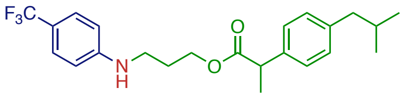

5t
ibuprofen derived

*3-((4-(trifluoromethyl)phenyl)amino)propyl 2-(4-isobutylphenyl)propanoate (**5t**).* Prepared from 1-((4-(trifluoromethyl)phenyl)amino)pyridin-1-ium triflate (**3a**) and 3-iodopropyl 2-(4-isobutylphenyl)propanoate (**4t**) at 70 °C and obtained as a colorless oil (23.6 mg, 58% yield). ^{1}H NMR (δ, 23 °C, 400 MHz, $CDCl_3$): 7.36 (d, J = 8.4 Hz, 2H), 7.21 (d, J = 7.9 Hz, 2H), 7.10 (d, J = 7.9 Hz, 2H), 6.48 (d, J = 8.4 Hz, 2H), 4.24–4.05 (m, 3H), 3.74–3.69 (m, 1H), 3.09 (t, J = 6.6 Hz, 2H), 2.44 (d, J = 7.2 Hz, 2H), 1.85 (td, J = 13.2, 6.5 Hz, 3H), 1.51 (d, J = 7.1 Hz, 3H), 0.88 (d, J

= 6.6 Hz, 6H). ^{13}C NMR (δ, 23 °C, 100 MHz, $CDCl_3$): 174.9, 150.5, 140.9, 137.8, 129.6, 127.2, 126.7, 126.7, 123.8 (q, $^1J_{C–F}$ = 269 Hz), 118.7 (q, $^2J_{C–F}$ = 32 Hz), 111.9, 62.3, 45.3, 45.1, 40.2, 30.3, 28.3, 22.5, 18.5. ^{19}F NMR (δ, 23 °C, 376 MHz, $CDCl_3$): –61.0. HRMS (APCI) m/z: $[M+H]^+$ Calculated for $C_{23}H_{29}F_3NO_2$ 408.2145, Found 408.2137.

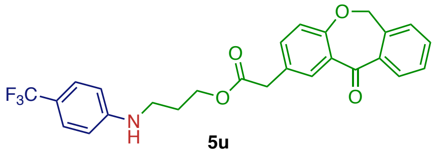

5u
isoxepac derived

*3-((4-(trifluoromethyl)phenyl)amino)propyl 2-(11-oxo-6,11-dihydrodibenzo[b,e]oxepin-2-yl) acetate (**5u**)*. Prepared from 1-((4-(trifluoromethyl)phenyl)amino)pyridin-1-ium triflate (**3a**) and 3-iodopropyl 2-(11-oxo-6,11-dihydrodibenzo[b,e]oxepin-2-yl)acetate (**4u**) at 70 °C and obtained as a white solid (19.7 mg, 42% yield). ^{1}H NMR (δ, 23 °C, 400 MHz, $CDCl_3$): 8.15–8.14 (m, 1H), 7.87 (dd, J = 7.7, 1.2 Hz, 1H), 7.56 (dd, J = 7.5, 1.4 Hz, 1H), 7.49–7.34 (m, 5H), 7.04 (d, J = 8.4 Hz, 1H), 6.55 (d, J = 8.5 Hz, 2H), 5.16 (s, 2H), 4.24 (t, J = 6.0 Hz, 2H), 3.67 (s, 2H), 3.21 (t, J = 6.8 Hz, 2H), 1.95 (quintet, J = 6.3 Hz, 2H). ^{13}C NMR (δ, 23 °C, 100 MHz, $CDCl_3$): 191.0, 171.5, 160.7, 150.4, 140.5, 136.4, 135.7, 133.0, 132.5, 129.6, 129.5, 128.0, 127.8, 126.8, 126.7, 125.3, 123.8 (q, $^1J_{C–F}$ = 269 Hz), 121.3, 118.7 (q, $^2J_{C–F}$ = 32 Hz), 112.0, 73.8, 62.7, 40.5, 40.4, 28.3. ^{19}F NMR (δ, 23 °C, 376 MHz, $CDCl_3$): –61.0. HRMS (APCI) m/z: $[M+H]^+$ Calculated for $C_{26}H_{23}F_3NO_4$ 470.1574, Found 470.1564.

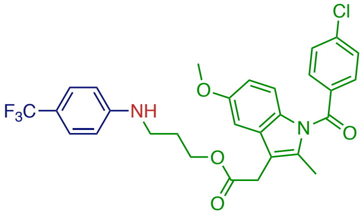

5v
indomethacin derived

*3-((4-(trifluoromethyl)phenyl)amino)propyl 2-(1-(4-chlorobenzoyl)-5-methoxy-2-methyl-1H-indol-3-yl)acetate (**5v**)*. Prepared from 1-((4-(trifluoromethyl)phenyl)amino)pyridin-1-ium triflate (**3a**) and 3-iodopropyl 2-(1-(4-chlorobenzoyl)-5-methoxy-2-methyl-1H-indol-3-yl)acetate (**4v**) at 70 °C and obtained as a pale-yellow solid (25.1 mg, 45% yield). ^{1}H NMR (δ, 23 °C, 400 MHz, $CDCl_3$): 7.63 (d, J = 8.4 Hz, 2H), 7.44 (d, J = 8.4 Hz, 2H), 7.34 (d, J = 8.5 Hz, 2H), 6.97 (d, J = 2.4 Hz, 1H), 6.84 (d, J = 9.0 Hz, 1H), 6.68 (dd, J = 9.0, 2.3 Hz, 1H), 6.46 (d, J = 8.5 Hz, 2H), 4.23 (t, J = 6.0 Hz, 2H), 3.81 (s, 3H), 3.69 (s, 2H), 3.13 (t, J = 6.7 Hz, 2H), 2.41 (s, 3H), 1.92 (t, J = 6.3 Hz, 2H). ^{13}C NMR (δ, 23 °C, 100 MHz, $CDCl_3$): 171.0, 168.4, 156.2, 150.4, 139.5, 136.2, 133.9, 131.3, 131.0, 130.7, 129.3, 126.7, 123.7 (q, $^1J_{C–F}$ = 269 Hz), 118.7 (q, $^2J_{C–F}$ = 32 Hz), 115.2, 112.5, 111.9, 111.6, 101.6, 62.7, 55.9, 40.2, 30.6, 28.4, 13.5. ^{19}F NMR (δ, 23 °C, 376 MHz, $CDCl_3$): –61.0. HRMS (APCI) m/z: $[M+H]^+$ Calculated for $C_{29}H_{27}ClF_3N_2O_4$ 559.1606, Found 559.1596.

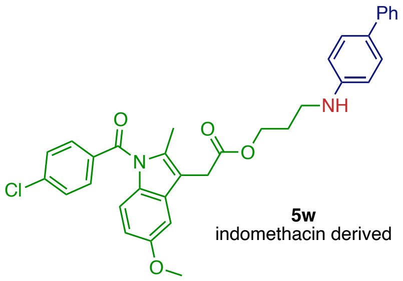

5w
indomethacin derived

*3-([1,1'-biphenyl]-4-ylamino)propyl 2-(1-(4-chlorobenzoyl)-5-methoxy-2-methyl-1H-indol-3-yl) acetate (**5w**).* Prepared from 1-([1,1'-biphenyl]-4-ylamino)pyridin-1-ium triflate (**3c**) and 3-iodopropyl 2-(1-(4-chlorobenzoyl)-5-methoxy-2-methyl-1H-indol-3-yl)acetate (**4v**) at 45 °C and obtained as a pale-yellow oil (44.1 mg, 78% yield). ^{1}H NMR (δ, 23 °C, 400 MHz, $CDCl_3$): 7.63 (d, J = 8.6 Hz, 2H), 7.52–7.49 (m, 2H), 7.40 (td, J = 8.4, 6.4 Hz, 6H), 7.28 (dt, J = 7.3, 1.5 Hz, 1H), 6.98 (d, J = 2.5 Hz, 1H), 6.85 (d, J = 9.0 Hz, 1H), 6.68 (td, J = 5.8, 2.9 Hz, 3H), 4.25 (t, J = 6.1 Hz, 2H), 3.82 (d, J = 2.2 Hz, 3H), 3.69 (s, 2H), 3.17 (t, J = 6.9 Hz, 2H), 2.41 (s, 3H), 1.99 (t, J = 6.4 Hz, 2H). ^{13}C NMR (δ, 23 °C, 100 MHz, $CDCl_3$): 171.0, 168.4, 156.2, 141.0, 139.4, 136.2, 133.9, 131.3, 131.0, 130.7, 129.3, 128.8, 128.2, 126.5, 115.2, 112.6, 111.7, 101.5, 62.8, 56.1, 55.9, 30.6, 28.2, 13.5. HRMS (APCI) m/z: $[M+H]^+$ Calculated for $C_{34}H_{32}ClN_2O_4$ 567.2045, Found 567.2035.

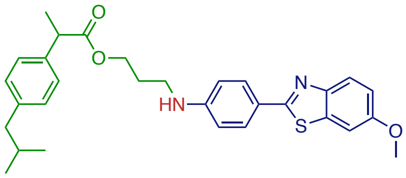

5x
thioflavin T- ibuprofen conjugate

*3-((4-(6-methoxybenzo[d]thiazol-2-yl)phenyl)amino)propyl 2-(4-isobutylphenyl)propanoate (**5x**).* Prepared from 1-((4-(6-methoxybenzo[d]thiazol-2-yl)phenyl)amino)pyridin-1-ium triflate (**3q)** and 3-iodopropyl 2-(4-isobutylphenyl)propanoate (**4t**) at 70 °C and obtained as a yellow solid (35.2 mg, 70% yield). ^{1}H NMR (δ, 23 °C, 400 MHz, $CDCl_3$): 7.85 (dd, J = 16.5, 8.8 Hz, 3H), 7.32 (d, J = 2.5 Hz, 1H), 7.22 (d, J = 8.1 Hz, 2H), 7.11 (d, J = 8.0 Hz, 2H), 7.04 (dd, J = 8.9, 2.6 Hz, 1H), 6.53 (d, J = 8.8 Hz, 2H), 4.27–4.14 (m, 2H), 3.87 (s, 3H), 3.72 (q, J = 7.1 Hz, 1H), 3.13 (t, J = 6.7 Hz, 2H), 2.45 (d, J = 7.2 Hz, 2H), 1.93–1.80 (m, 3H), 1.51 (d, J = 7.2 Hz, 3H), 0.88 (d, J = 6.6 Hz, 6H). ^{13}C NMR (δ, 23 °C, 100 MHz, $CDCl_3$): 174.9, 166.5, 157.3, 150.2, 140.9, 137.8, 135.8, 129.68, 129.57, 128.9, 127.41, 127.22, 122.9, 115.1, 112.5, 104.5, 62.4, 55.9, 45.34, 45.15, 40.3, 30.3, 28.4, 22.5, 18.5. HRMS (APCI) m/z: $[M+H]^+$ Calculated for $C_{30}H_{35}N_2O_3S$ 503.2363, Found 503.2355.

B.3 One-pot Reaction Protocol for the Synthesis of 5b

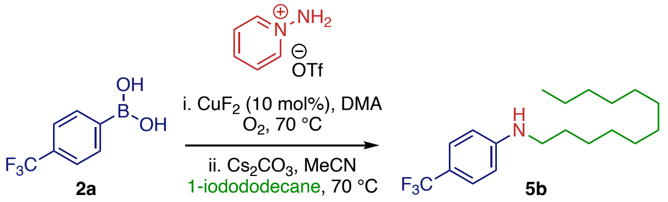

A 20-mL scintillation vial was charged with *N*-aminopyridinium triflate (244 mg, 1.00 mmol, 1.00 equiv), (4-(trifluoromethyl)phenyl)boronic acid **2a** (189.5 mg, 1.00 mmol, 1.00 equiv), copper(II) fluoride (5.00 mg, 0.050 mmol, 0.050 equiv), *N,N*-dimethylacetamide (DMA, 0.5 mL). A magnetic stir bar was added, and the vial was fitted with a rubber septum. Oxygen was bubbled through the reaction mixture for 1 h at 23 °C. Under an O_2 atmosphere, the mixture was heated at 70 °C inside a reaction block for 19 h. The reaction mixture was cooled to 23 °C and O_2 was again bubbled through the reaction mixture for 1 h at 23 °C. (4-(Trifluoromethyl)phenyl)boronic acid **2a** (189.5 mg, 1.00 mmol, 1.00 equiv) and copper(II) fluoride (5.00 mg, 0.050 mmol, 0.050 equiv) were added. Under an O_2 atmosphere, the mixture was heated at 70 °C inside a reaction block for an additional 19 h. After this time, the reaction mixture was cooled to 23 °C and diluted with acetonitrile (10.0 mL). Cesium carbonate (977 mg, 3.00 mmol, 3.00 equiv) and 1-iodododecane (592 mg, 2.00 mmol, 2.00 equiv) were added to the reaction mixture under air. The reaction vial was sealed and heated at 70 °C inside a reaction block for 16 h. After cooling to 23 °C, the reaction mixture was concentrated under reduced pressure. The residue was diluted with 40 mL of CH_2Cl_2:water (1:1) and the aqueous layer was extracted with CH_2Cl_2 (20 mL × 3). The combined organic layer was washed with brine, dried over Na_2SO_4 and purified by SiO_2 gel chromatography (eluent 100:0 hexanes:EtOAc to 95:5 hexanes:EtOAc) to afford the title compound **5b** as a pale-yellow oil (135 mg, 41% yield).

C. Mechanistic Studies

C.1 Synthesis of Ylide 3a’

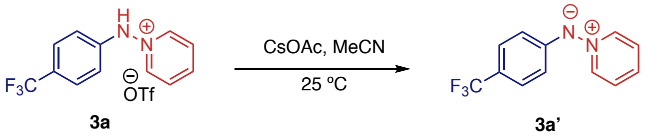

A 20-mL scintillation vial was charged with 1-((4-(trifluoromethyl)phenyl)amino)-pyridin-1-ium triflate (**3a**, 39 mg, 0.10 mmol, 1.0 equiv), cesium acetate (58 mg, 0.30 mmol, 3.0 equiv), and a magnetic stir bar in an N_2-filled dry box. Acetonitrile (1.0 mL) was then added to the reaction mixture. The reaction vial was sealed under N_2 atmosphere and stirred at 25 °C for 2 h. After this time, the reaction mixture was filtered and concentrated under reduced pressure to obtain 1-((4-(trifluoromethyl)phenyl)amino)-pyridin-1-ium ylide **3a’** (22 mg, 90% yield). ^{1}H NMR (δ, 23 °C, 400 MHz, CD_3CN): 8.54 (d, J = 5.9 Hz, 2H), 7.72 (t, J = 7.6 Hz, 1H), 7.60 (t, J = 7.0 Hz, 2H), 7.27 (d, J = 8.5 Hz, 2H), 6.60 (d, J = 8.5 Hz, 2H).

C.2 Reaction of 1-Iodohexane with Ylide 3a’

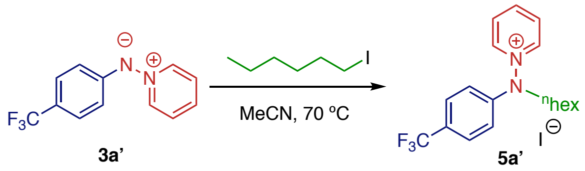

A 20-mL scintillation vial was charged with compound **3a’** (12 mg, 0.05 mmol, 1.0 equiv) and MeCN (1.0 mL). 1-Iodohexane (21 mg, 0.10 mmol, 2.0 equiv) was added to the reaction vial and the vial was sealed under N_2 atmosphere and stirred at 70 °C inside a reaction block for 16 h. After cooling to 23 °C, the reaction mixture was concentrated under reduced pressure. CD_3CN was added to the residue followed by 1,3,5-trimethoxybenzene as the internal standard to determine the yield of **5a’** (95%).

C.3 Unproductive Alkylation of 6 or 5a

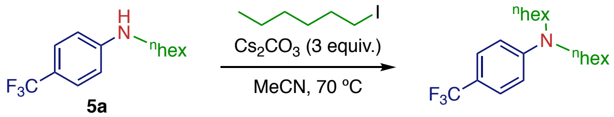

A 20-mL scintillation vial was charged with *N*-hexyl-4-(trifluoromethyl)aniline (**5a**, 24 mg, 0.10 mmol, 1.0 equiv), cesium carbonate (98 mg, 0.30 mmol, 3.0 equiv), and a magnetic stir bar in an N_2-filled dry box. 1-Iodohexane (42 mg, 0.20 mmol, 2.0 equiv) and acetonitrile (1.5 mL) was added to the reaction vessel. The reaction vial was sealed under N_2 atmosphere and heated at 70 °C inside a reaction block for 16 h. After this time, the reaction mixture was concentrated under reduced pressure. CD_3CN was added to the residue followed by 1,3,5-trimethoxybenzene as the internal standard. The NMR showed complete recovery of the starting materials, *i.e.* **5a** (100%).

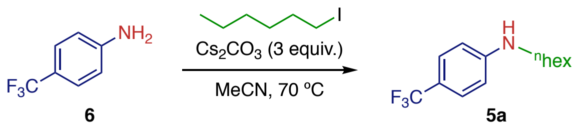

6 5a

A 20-mL scintillation vial was charged with 4-(trifluoromethyl)aniline (**6**, 16 mg, 0.10 mmol, 1.0 equiv), cesium carbonate (98 mg, 0.30 mmol, 3.0 equiv), and a magnetic stir bar in an N_2-filled dry box. 1-iodohexane (42 mg, 0.20 mmol, 2.0 equiv) and acetonitrile (1.5 mL) was added to the reaction vessel. The reaction vial was sealed under N_2 atmosphere and heated at 70 °C inside a reaction block for 16 h. After this time, the reaction mixture was concentrated under reduced pressure. CD_3CN was added to the residue followed by 1,3,5-trimethoxybenzene as the internal standard to determine the yield of *N*-hexyl-4-(trifluoromethyl)aniline **5a** (10%) and 80% 4-(trifluoromethyl)aniline was recovered.

Similar reactions of aniline (at 50 °C) and 4-*tert*-butylaniline (at 40 °C) were conducted with 1-iodohexane. We obtained the respective secondary amine products **5n** and **5o** in 18% and 25% yields, respectively.

C.4 Alkylation of 3a with 1-Iodohexane using CsOAc

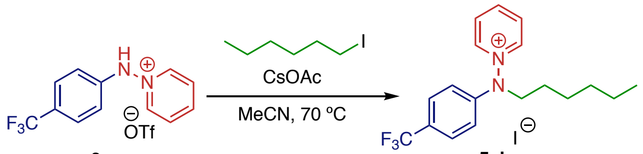

3a 5a'

A 20-mL scintillation vial was charged with 1-((4-(trifluoromethyl)phenyl)amino)-pyridin-1-ium triflate (**3a**, 117 mg, 0.300 mmol, 1.00 equiv), cesium acetate (174 mg, 0.900 mmol, 3.00 equiv), and a magnetic stir bar in an N_2-filled dry box. 1-Iodohexane (126 mg, 0.600 mmol, 2.00 equiv) and acetonitrile (5 mL) was then added to the reaction vessel. The reaction vial was sealed under N_2 atmosphere and heated at 70 °C inside a reaction block for 16 h. After cooling to 23 °C, the reaction mixture was concentrated under reduced pressure. The residue was purified by SiO_2 gel chromatography (eluent 100:0 CH_2Cl_2:MeOH to 98:2 CH_2Cl_2:MeOH) to afford **5a'** as a yellow solid (128 mg, 95% yield). ^1H NMR (δ, 23 °C, 400 MHz, CD_3CN): 8.98 (dd, J = 6.8, 1.3 Hz, 2H), 8.78 (t, J = 7.8 Hz, 1H), 8.28 (dd, J = 7.8, 6.9 Hz, 2H), 7.70 (dd, J = 9.1, 0.6 Hz, 2H), 6.96 (d, J = 8.5 Hz, 2H), 3.99 (t, J = 7.4 Hz, 2H), 1.56 (dt, J = 14.6, 7.5 Hz, 2H), 1.48-1.41 (m, 2H), 1.32 (dt, J = 7.0, 3.6 Hz, 4H), 0.91 (t, J = 7.0 Hz, 3H). ^{13}C NMR (δ, 23 °C, 100 MHz, CD_3CN): 150.2, 149.4, 148.4, 131.7, 128.0, 127.9, 117.4, 56.2, 32.0, 27.4, 26.7, 23.2, 14.2. HRMS (APCI) m/z: $[M]^+$ Calculated for $C_{18}H_{22}F_3N_2^+$ 323.1730, Found 323.1727.

C.5 Removal of Pyridine from 5a' using Excess Cs_2CO_3

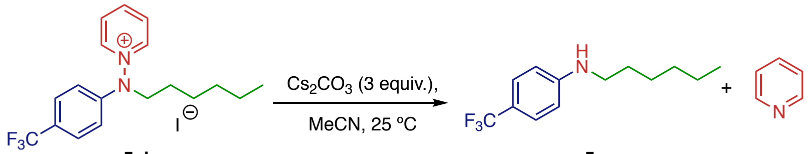

5a' 5a

A 20-mL scintillation vial was charged with 1-(hexyl(4-(trifluoromethyl)phenyl)amino)pyridin-1-ium iodide (**5a'**, 45 mg, 0.10 mmol, 1.0 equiv), cesium carbonate (96 mg, 0.30 mmol, 3.0 equiv), and a magnetic stir bar in an N_2-filled dry box. Acetonitrile (1.5 mL) was then added to the reaction mixture. The reaction vial was sealed under N_2 atmosphere and stirred at 25 °C for 16 h. After this

time, the reaction mixture was concentrated under reduced pressure. CD_3CN was added to the residue followed by 1,3,5-trimethoxybenzene as the internal standard to determine the yield of **5a** (79%) and pyridine (26%).

C.6 Alkylation of 3a with 1-Iodohexane using 1 Equivalent of Cs_2CO_3

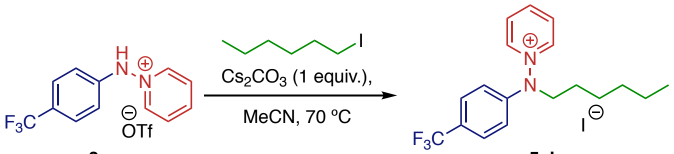

3a **5a’**

A 20-mL scintillation vial was charged with 1-((4-(trifluoromethyl)phenyl)amino)-pyridin-1-ium triflate (**3a**, 39 mg, 0.10 mmol, 1.0 equiv), cesium carbonate (32 mg, 0.10 mmol, 1.0 equiv), and a magnetic stir bar in an N_2-filled dry box. 1-Iodohexane (42 mg, 0.20 mmol, 2.0 equiv) and acetonitrile (1.5 mL) was then added to the reaction vessel. The reaction vial was sealed under N_2 atmosphere and heated at 70 °C inside a reaction block for 16 h. After cooling to 23 °C, the reaction mixture was concentrated under reduced pressure. CD_3CN was added to the residue followed by 1,3,5-trimethoxybenzene as the internal standard. The yield of **5a’** (78%) was determined by ^1H NMR spectroscopy.

C.7 Depyridylation of 5a’ in Presence of Reducing CO_3^{2-} Source

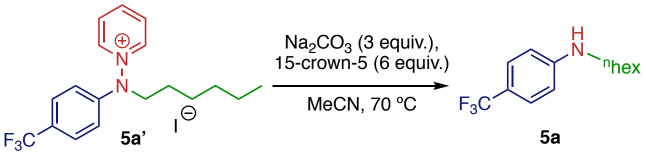

5a’ **5a**

A 20-mL scintillation vial was charged with 1-(hexyl(4-(trifluoromethyl)phenyl)amino)pyridin-1-ium iodide (**5a’**, 45 mg, 0.10 mmol, 1.0 equiv), sodium carbonate (32 mg, 0.30 mmol, 3.0 equiv), and a magnetic stir bar in an N_2-filled dry box. 15-Crown-5 ether (0.13 g, 0.60 mmol, 6.0 equiv) and acetonitrile (1.5 mL) was then added to the reaction mixture. The reaction vial was sealed under N_2 atmosphere and stirred at 70 °C inside a reaction block for 16 h. After this time, the reaction mixture was concentrated under reduced pressure. CD_3CN was added to the residue followed by 1,3,5-trimethoxybenzene as the internal standard to determine the yield of *N*-hexyl-4-(trifluoromethyl)aniline (60%). The headspace of the reaction mixture was also analyzed by gas chromatography. Data are collected in Figure S1.

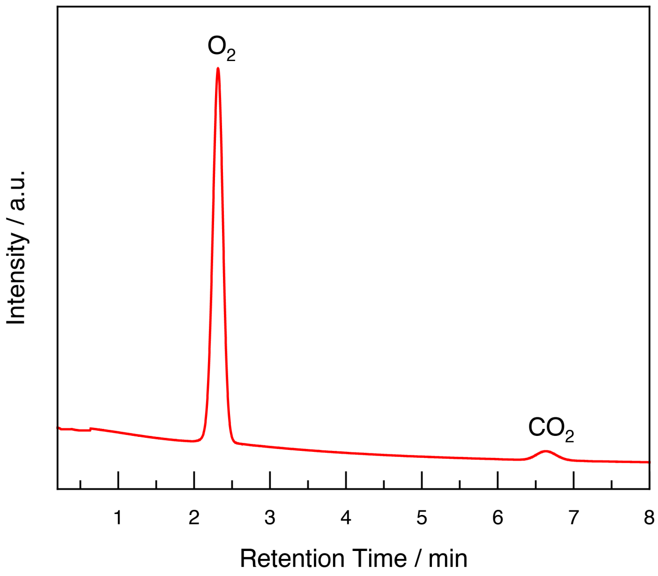

Figure S1. The gas chromatogram illustrates the headspace of the reaction between **5a’** and Na_2CO_3 in the presence of 15-crown-5. The presence of CO_2 gas shows reduction by Na_2CO_3. The oxygen (O_2) peak is from air.

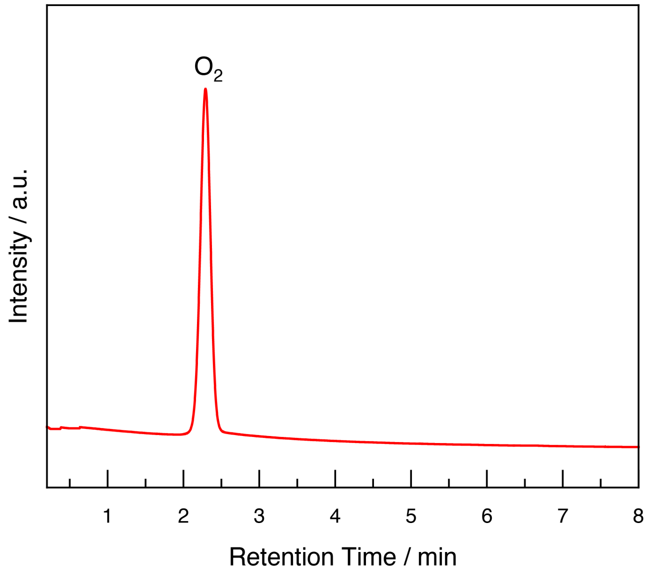

Figure S2. The gas chromatogram illustrates the headspace of the reaction between **5a’** and Na_2CO_3 in the absence of 15-crown-5. No CO_2 gas was detected in the headspace; the oxygen (O_2) peak is from air.

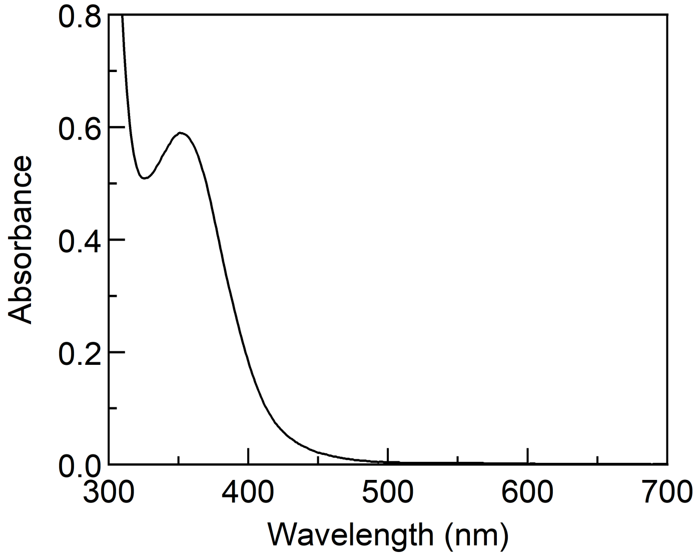

Figure S3. UV-vis spectra of a solution of **5a’** in MeCN.

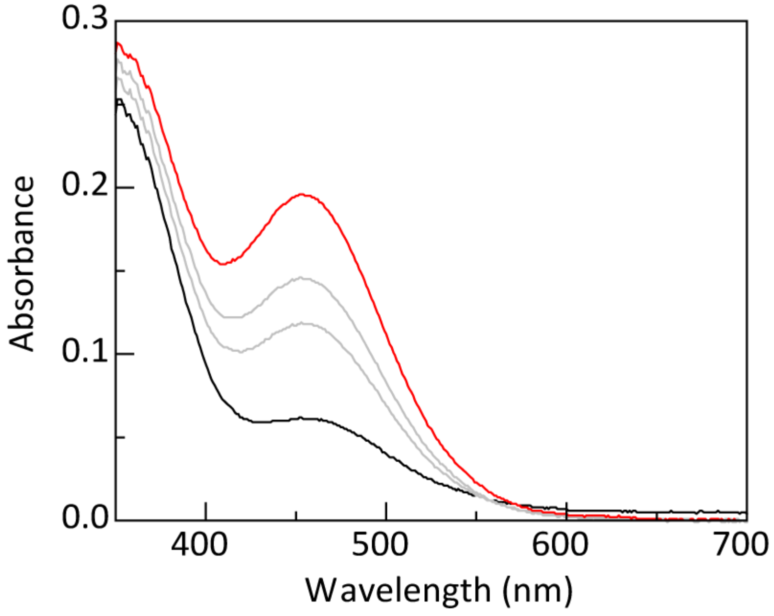

Figure S4. UV-vis spectra obtained by treatment of a solution of **5a’** and 10 equiv. of Cs_2CO_3 in MeCN. Gradual formation of a low-energy absorbance centered at 450 nm corresponds to the EDA complex **5’’**.

D. X-Ray Diffraction Data

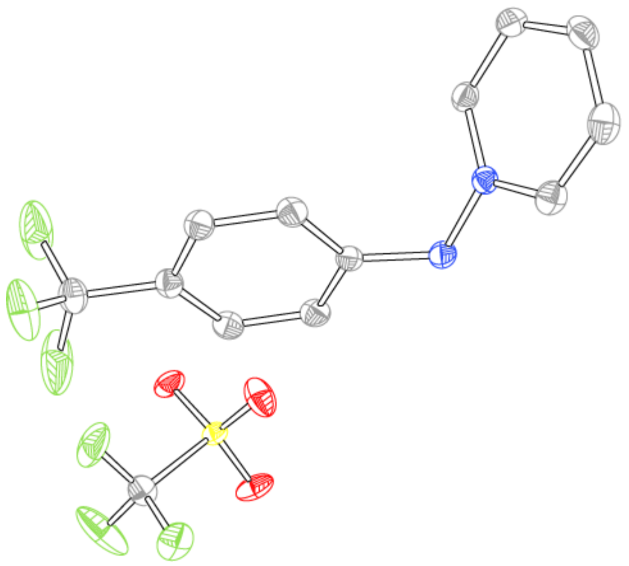

Figure S5. Displacement ellipsoid plot of **3a** plotted at 50% probability. H-atoms, and solvent are removed for clarity. The crystalline sample used in this diffraction experiment was obtained from a concentrated MeCN solution layered with Et_2O at −5 °C.

Table S1. X-ray experimental details of **3a** (CCDC 2284464).

Crystal data	
Chemical formula	$CF_3O_3S{\cdot}C_{12}H_{10}F_3N_2$
M_r	388.29
Crystal system, space group	Orthorhombic, *Pbca*
Temperature (K)	110
a, b, c (Å)	9.7003(5), 14.8142(8), 21.188(1)
V (Å^3)	3044.8(3)
Z	8
Radiation type	Mo $K\alpha$
μ (mm^{-1})	0.30
Crystal size (mm)	0.18 × 0.17 × 0.05
Data collection	
Diffractometer	Bruker *APEX*-II CCD
Absorption correction	Multi-scan<br>*SADABS2016*/2 (Bruker,2016/2) was used for absorption correction. wR2(int) was 0.0885 before and 0.0538 after correction. The Ratio of minimum to maximum transmission is 0.9011. The λ/2 correction factor is Not present.
T_{min}, T_{max}	0.672, 0.746
No. of measured, independent and observed [$I > 2\sigma(I)$] reflections	17246, 4657, 3512
R_{int}	0.043
$(\sin \theta/\lambda)_{max}$ (Å^{-1})	0.716
Refinement	
$R[F^2 > 2\sigma(F^2)]$, $wR(F^2)$, S	0.056, 0.154, 1.02
No. of reflections	4657
No. of parameters	226
H-atom treatment	H-atom parameters constrained
$\Delta\rho_{max}$, $\Delta\rho_{min}$ (e Å^{-3})	0.97, −0.68

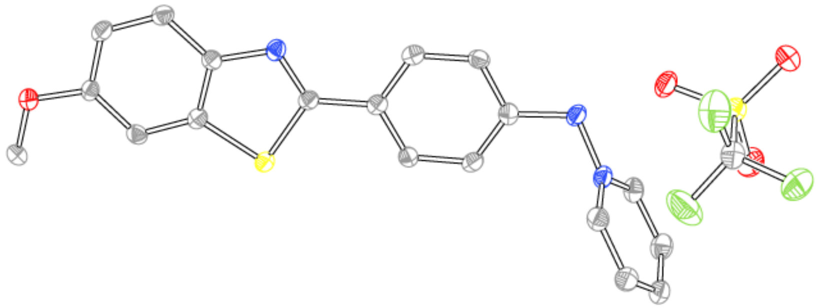

Figure S6. Displacement ellipsoid plot of **3q** plotted at 50% probability. H-atoms, and solvent are removed for clarity. The crystalline sample used in this diffraction experiment was obtained from a concentrated MeCN solution layered with Et_2O at −5 °C.

Table S2. X-ray experimental details of **3q** (CCDC 2284462).

Crystal data	
Chemical formula	$C_{19}H_{16}N_3OS \cdot CF_3O_3S$
M_r	483.48
Crystal system, space group	Triclinic, *P*1
Temperature (K)	110
a, *b*, *c* (Å)	7.2398(8), 7.833(1), 19.047(2)
α, β, γ (°)	87.563(4), 84.476(3), 71.770(3)
V ($Å^3$)	1021.1(2)
Z	2
Radiation type	Mo *K*α
μ (mm^{-1})	0.32
Crystal size (mm)	0.12 × 0.05 × 0.03
Data collection	
Diffractometer	Bruker Quest (PHOTON III) diffractometer.
Absorption correction	Multi-scan *SADABS2016*/2 (Bruker,2016/2) was used for absorption correction. wR2(int) was 0.1120 before and 0.0554 after correction. The Ratio of minimum to maximum transmission is 0.8566. The λ/2 correction factor is Not present.
T_{min}, T_{max}	0.638, 0.745
No. of measured, independent and observed [$I > 2\sigma(I)$] reflections	11241, 3902, 2797
R_{int}	0.048
$(\sin \theta/\lambda)_{max}$ ($Å^{-1}$)	0.612
Refinement	
$R[F^2 > 2\sigma(F^2)]$, $wR(F^2)$, S	0.047, 0.115, 1.04
No. of reflections	3902
No. of parameters	294
H-atom treatment	H atoms treated by a mixture of independent and constrained refinement
$\Delta\rho_{max}$, $\Delta\rho_{min}$ (e $Å^{-3}$)	0.28, −0.45

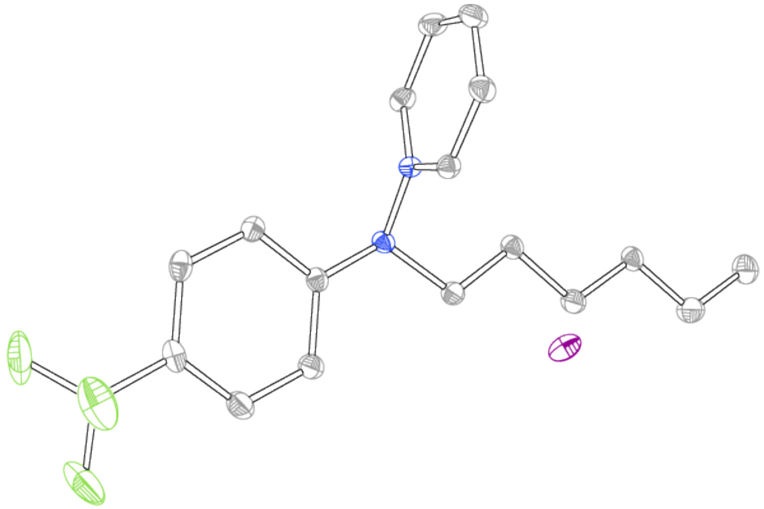

Figure S7. Displacement ellipsoid plot of **5a'** plotted at 50% probability. H-atoms, and solvent are removed for clarity. The crystalline sample used in this diffraction experiment was obtained from a concentrated MeCN solution layered with Et_2O at −5 °C.

Table S3. X-ray experimental details of **5a'** (CCDC 2284463).

Crystal data	
Chemical formula	$C_{18}H_{23}F_3N_2I$
M_r	451.28
Crystal system, space group	Monoclinic, $P2_1/c$
Temperature (K)	110
a, b, c (Å)	10.638(1), 9.4414(9), 18.804(2)
β (°)	92.006(3)
V (Å^3)	1887.4(3)
Z	4
Radiation type	Mo $K\alpha$
μ (mm^{-1})	1.73
Crystal size (mm)	0.2 × 0.2 × 0.14
Data collection	
Diffractometer	Bruker *APEX*-II CCD
Absorption correction	Multi-scan *SADABS2016*/2 (Bruker,2016/2) was used for absorption correction. wR2(int) was 0.1111 before and 0.0480 after correction. The Ratio of minimum to maximum transmission is 0.8431. The λ/2 correction factor is Not present.
T_{min}, T_{max}	0.629, 0.746
No. of measured, independent and observed [$I > 2\sigma(I)$] reflections	81423, 5777, 5547
R_{int}	0.031
$(\sin \theta/\lambda)_{max}$ (Å^{-1})	0.717
Refinement	
$R[F^2 > 2\sigma(F^2)]$, $wR(F^2)$, S	0.018, 0.045, 1.10
No. of reflections	5777
No. of parameters	218
H-atom treatment	H-atom parameters constrained
$\Delta\rho_{max}$, $\Delta\rho_{min}$ (e Å^{-3})	0.47, −1.00

E. Additional Data

E.1 Optimization Studies for the C_{sp2} *N*-Aminopyridylation

Table S4. Effect of deviation from the standard conditions for coupling boronic acids with *N*-aminopyridinium triflate

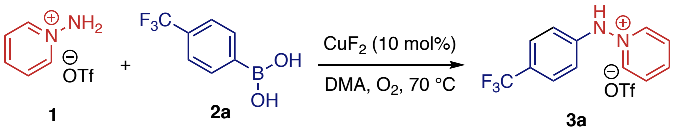

Entry	Deviation from Standard Conditions	Yield[a]
1	None	98%
2	Cu(OTf) as a catalyst	90%
3	$CuBr_2$ as a catalyst	15%
4	$Cu(acac)_2$ as a catalyst	21%
5	MeOH as a solvent	14%
6	DMF as a solvent	88%
7	DMSO as a solvent	13%
8	Using 5 mol% CuF_2	45%
9	Without O_2 bubbling	41%
10	50 °C instead of 70 °C	45%

[a] Determined by ^{1}H NMR spectroscopy using 1,3,5-trimethoxybenzene as internal standard

Table S5. Optimization of the reaction conditions for coupling (4-methoxyphenyl)boronic acid with *N*-aminopyridinium triflate.

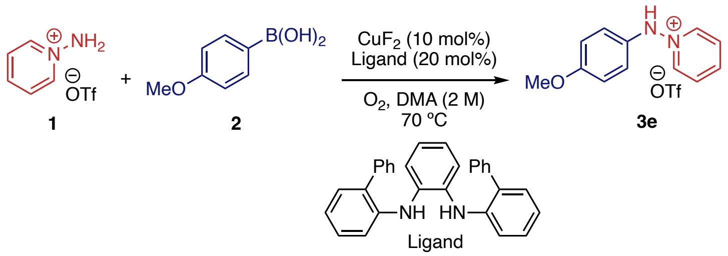

Entry	Deviation from Standard Conditions	Yield[a]
1	None	76%
2	No Ligand	54%
3	$Cu(OTf)_2$ as a catalyst	62%
4	Using 1 M DMA	44%
5	DMSO as a solvent	trace
6	50 °C instead of 70 °C	trace
7	TMEDA as a ligand	52%
8	2,2'-Bipyridine as a ligand	45%
9	Without O_2 bubbling	trace

[a] Determined by ^{1}H NMR using 1,3,5-trimethoxybenzene as internal standard

E.2 Summary of Unproductive Substrates

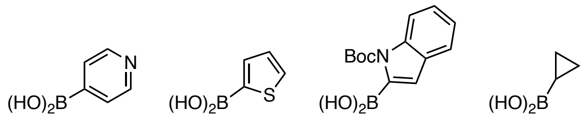

Figure S8. Summary of boronic acids that do not participate in productive Chan-Lam cross coupling under the optimized reaction conditions.

F. NMR Spectra for New Compounds

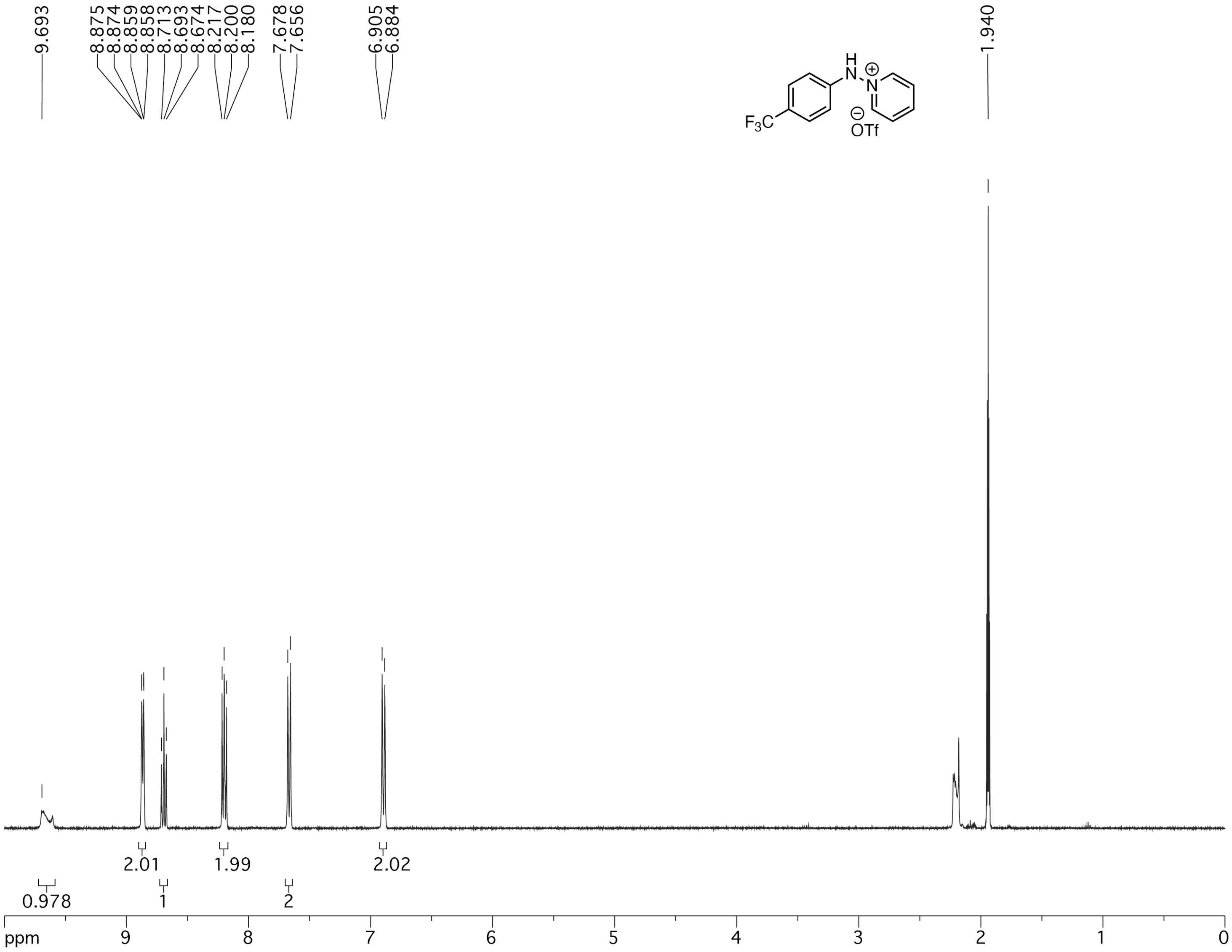

Figure S9. ^{1}H NMR spectrum of **3a** in CD_3CN (400 MHz) at 23 °C.

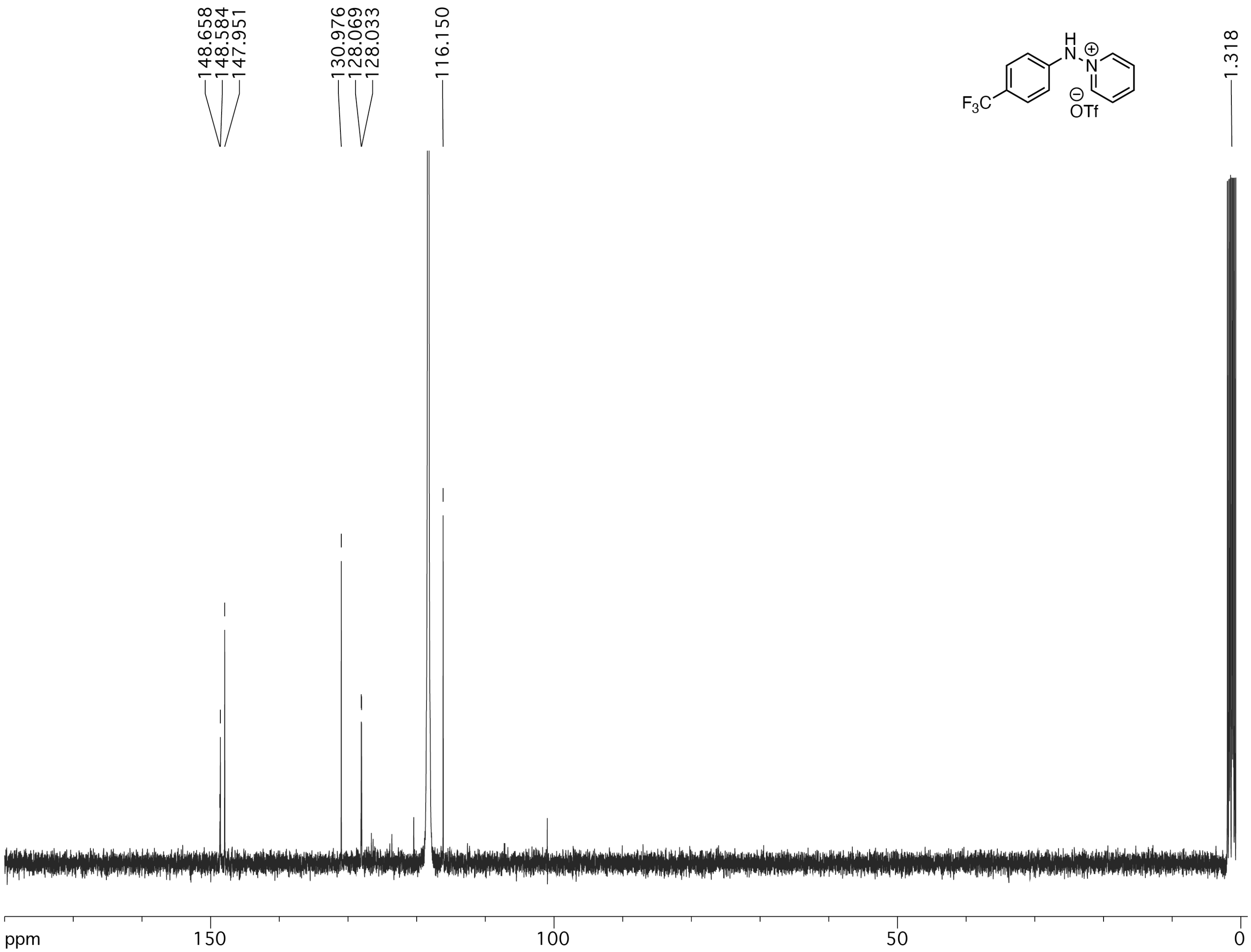

Figure S10. ^{13}C NMR spectrum of **3a** in CD_3CN (100 MHz) at 23 °C.

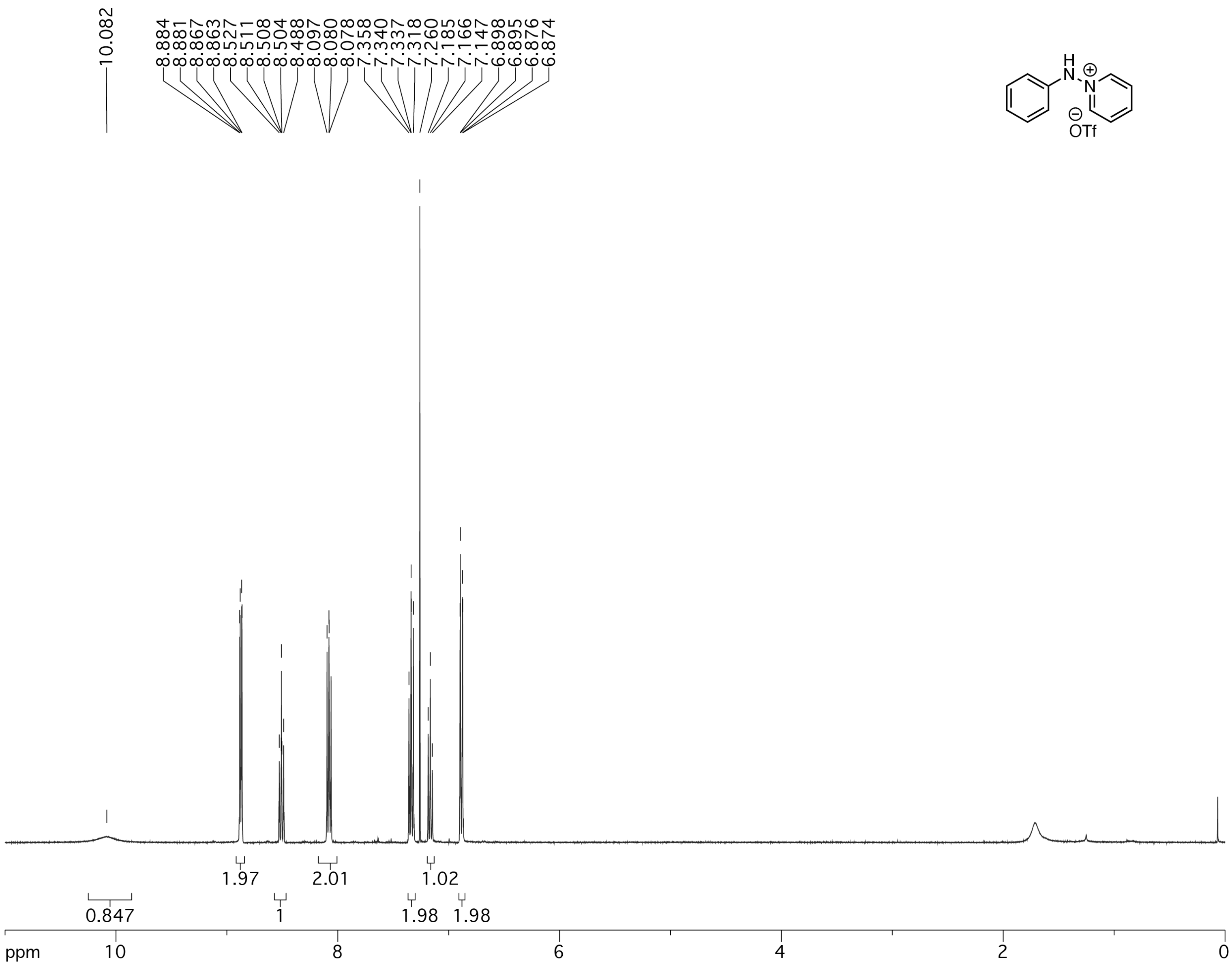

Figure S11. ^{1}H NMR spectrum of **3b** in $CDCl_3$ (400 MHz) at 23 °C.

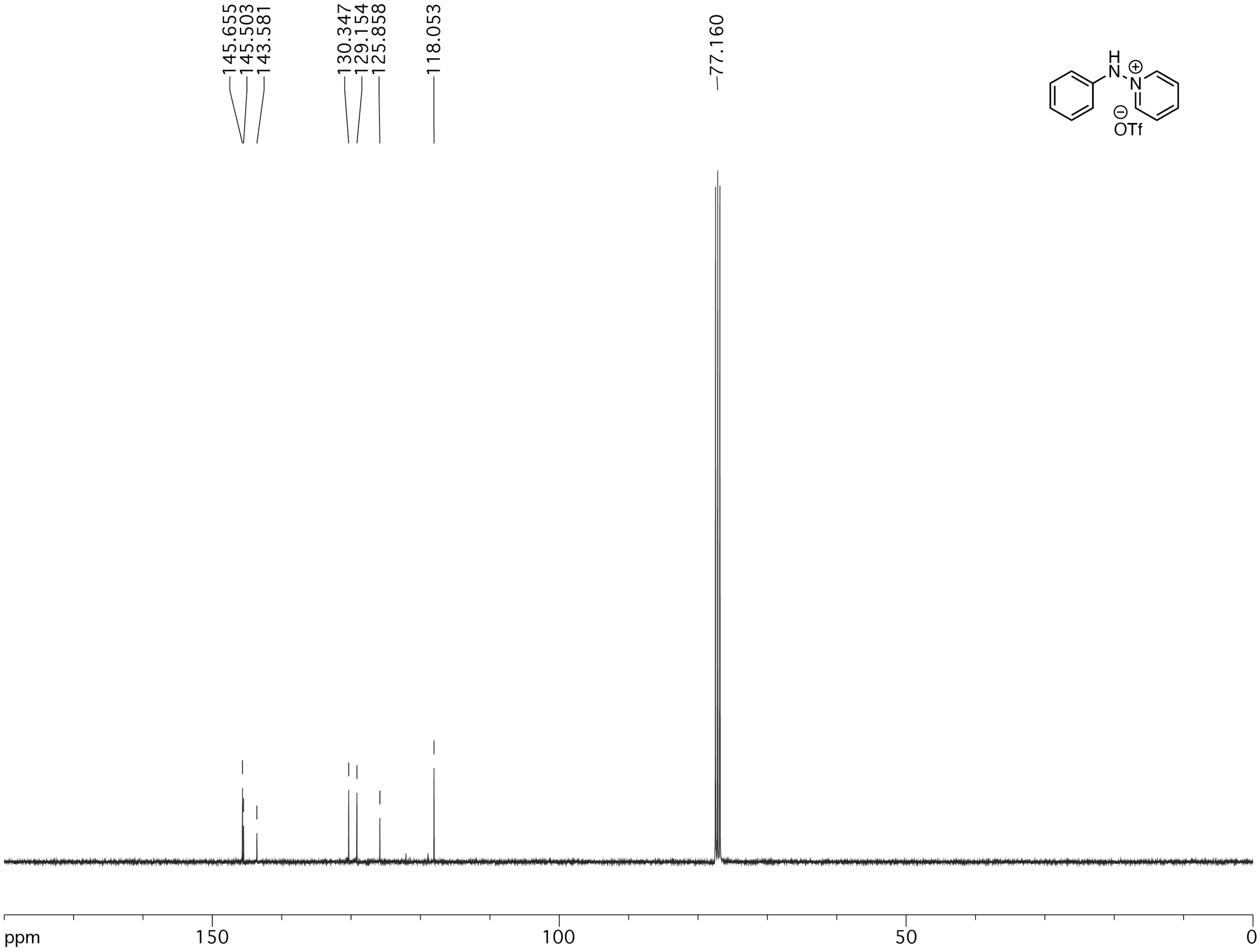

Figure S12. ^{13}C NMR spectrum of **3b** in $CDCl_3$ (100 MHz) at 23 °C.

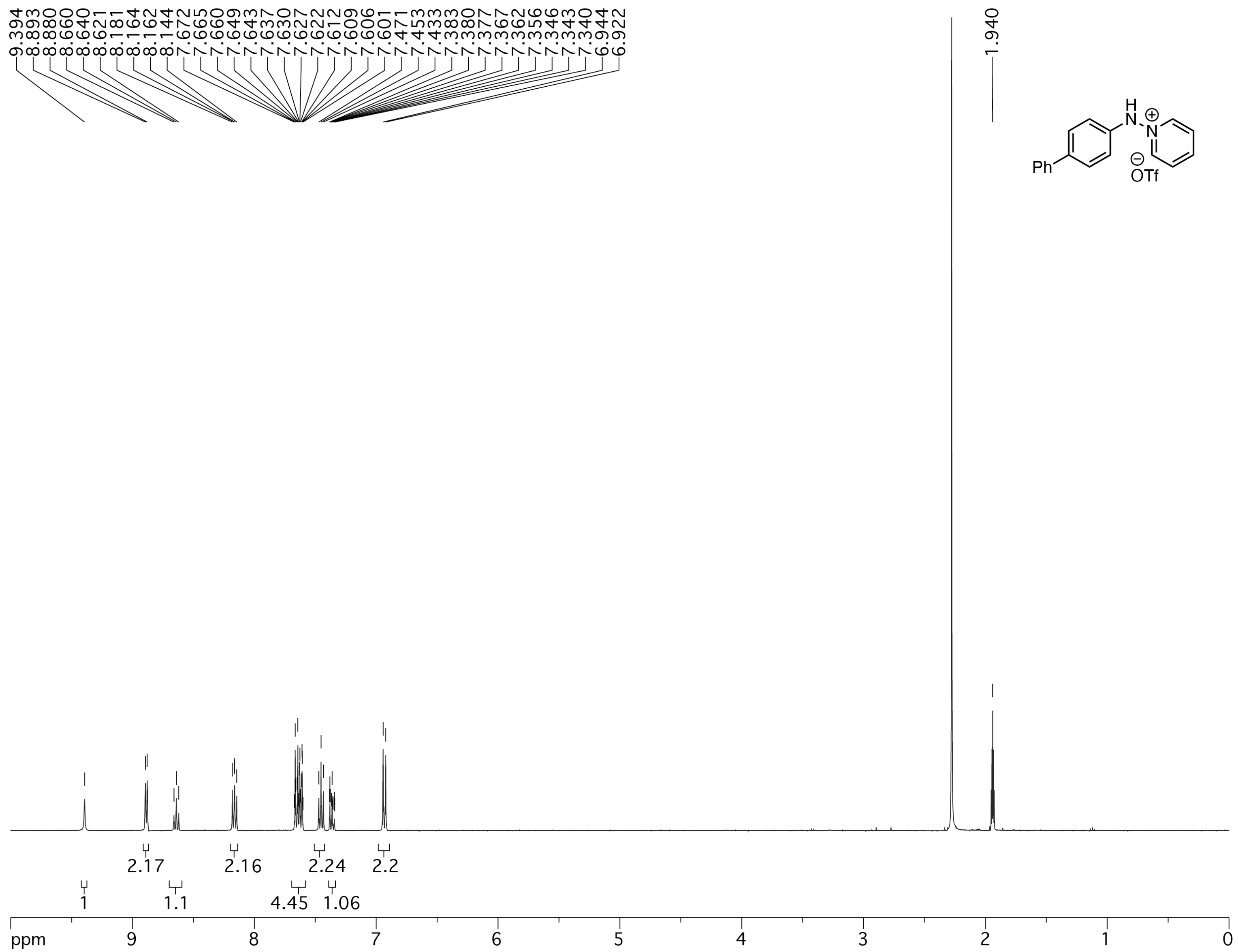

Figure S13. ^{1}H NMR spectrum of **3c** in CD_3CN (400 MHz) at 23 °C.

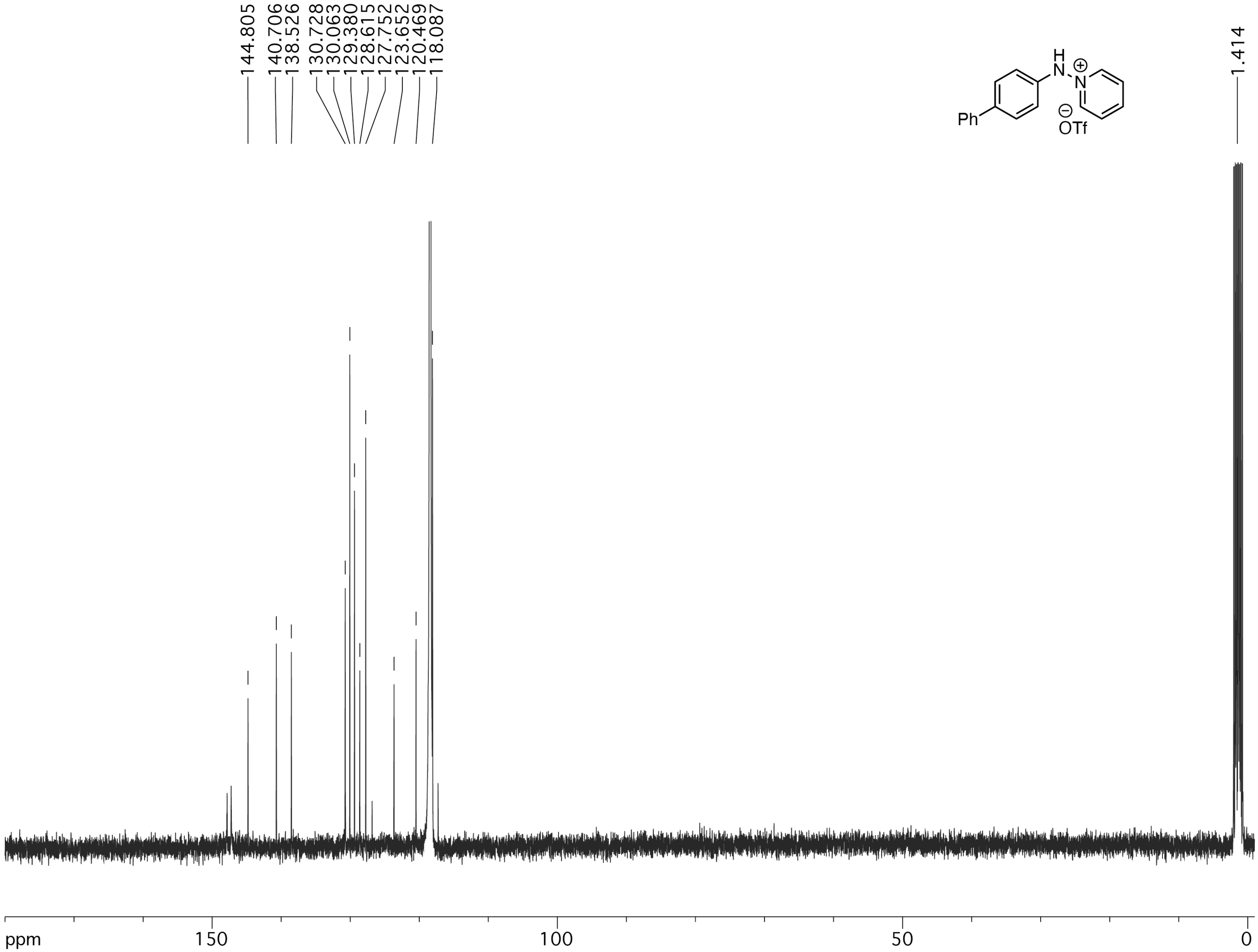

Figure S14. ^{13}C NMR spectrum of **3c** in CD_3CN (100 MHz) at 23 °C.

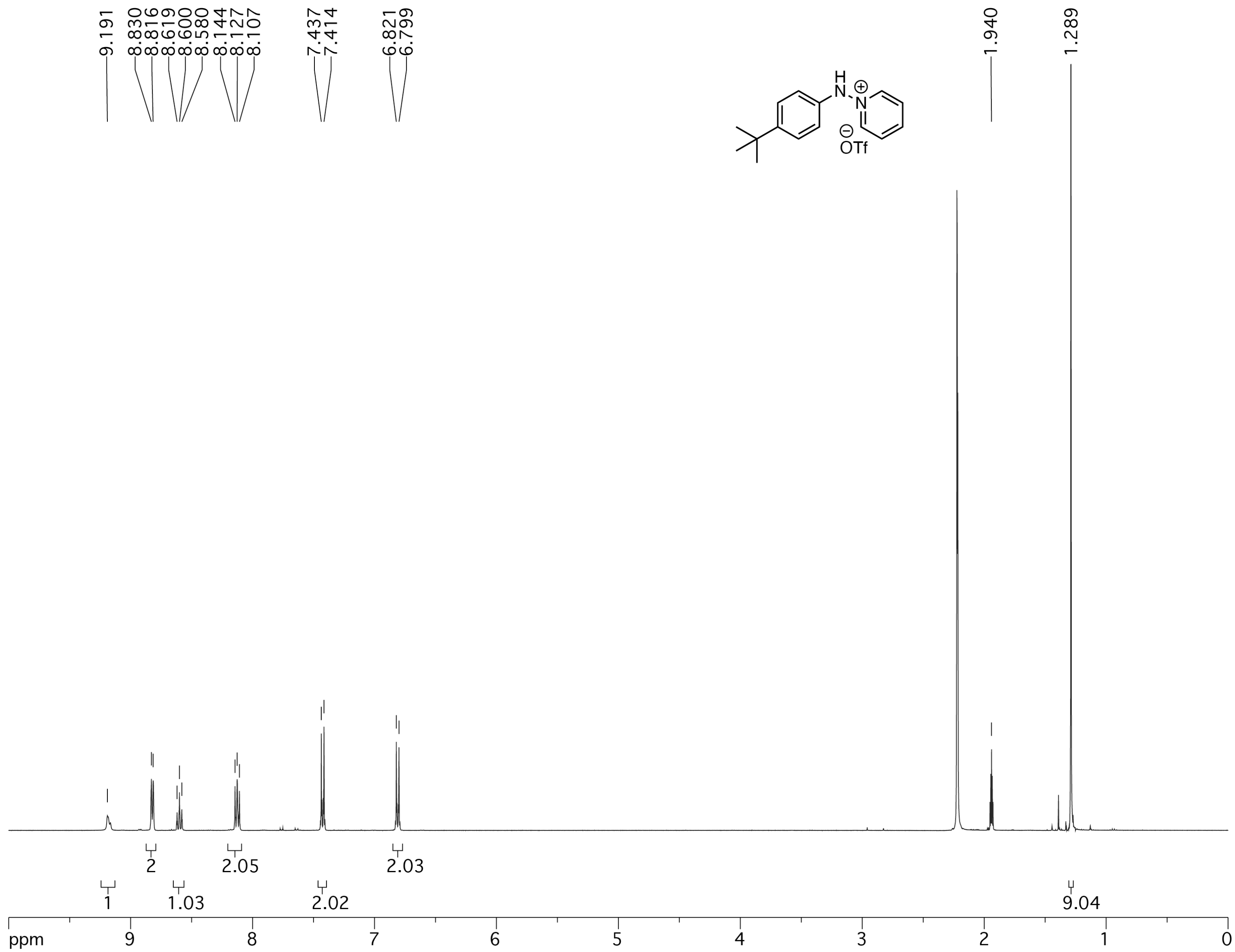

Figure S15. ^{1}H NMR spectrum of **3d** in CD_3CN (400 MHz) at 23 °C.

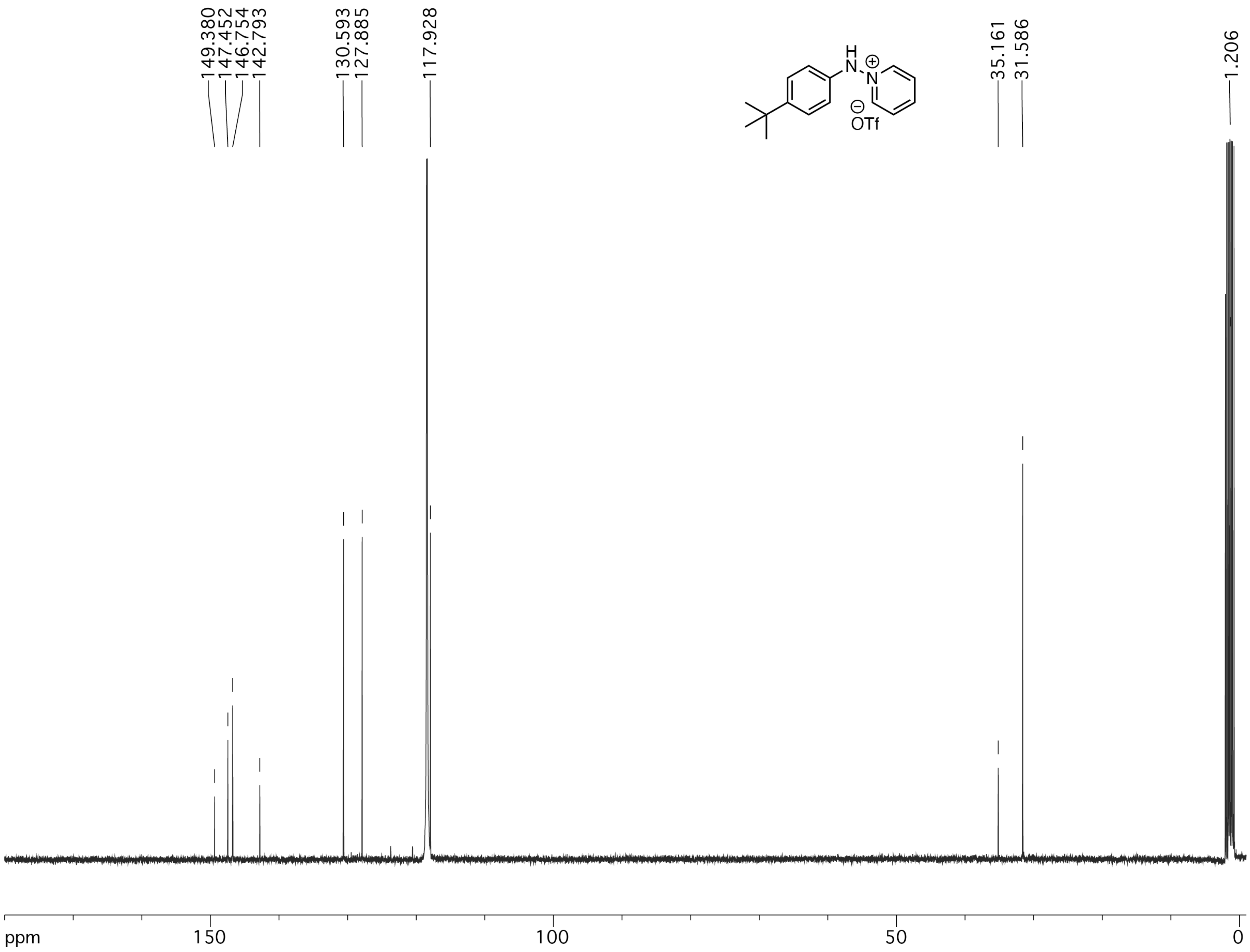

Figure S16. ^{13}C NMR spectrum of **3d** in CD_3CN (100 MHz) at 23 °C.

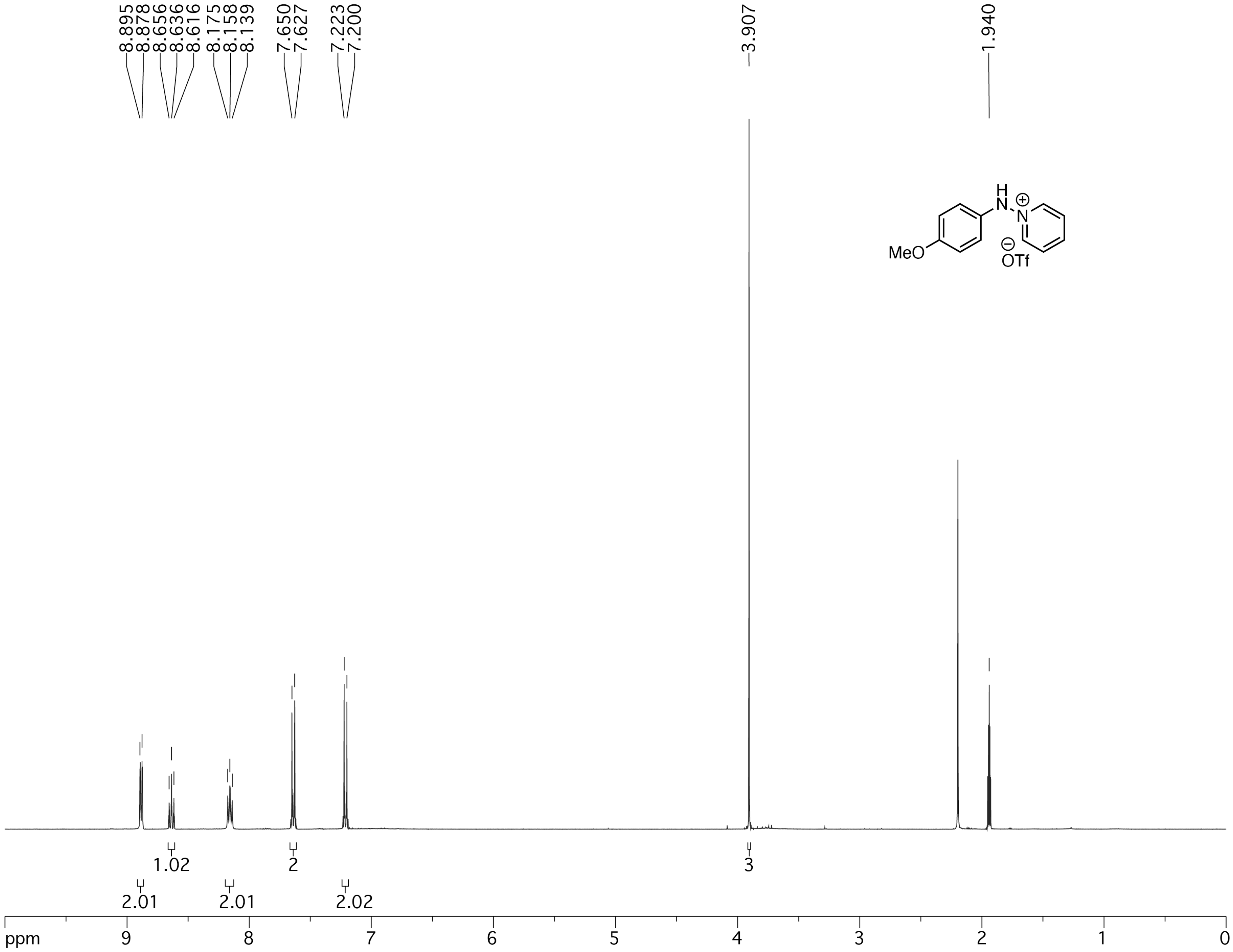

Figure S17. ^{1}H NMR spectrum of **3e** in CD_3CN (400 MHz) at 23 °C.

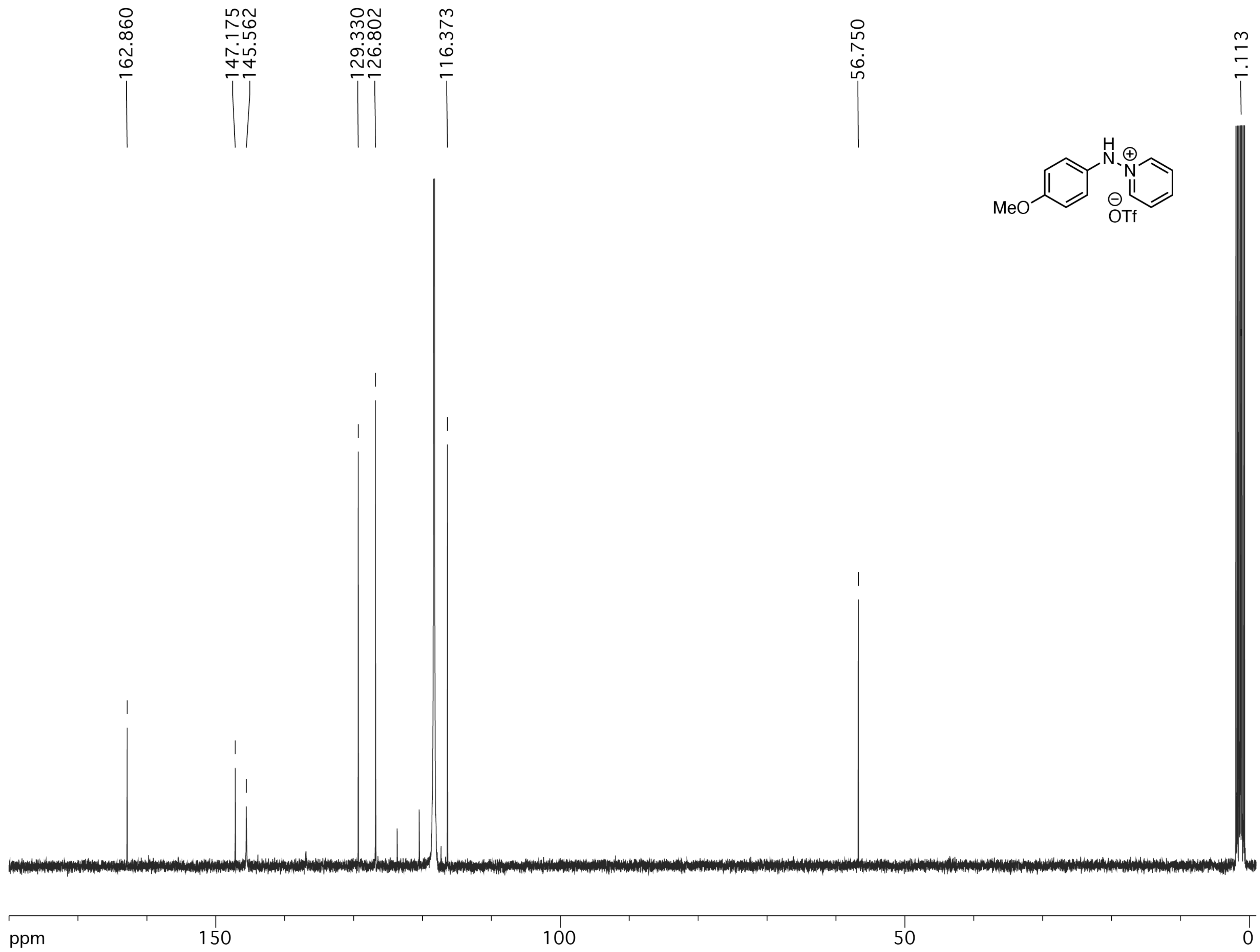

Figure S18. ^{13}C NMR spectrum of **3e** in CD_3CN (100 MHz) at 23 °C.

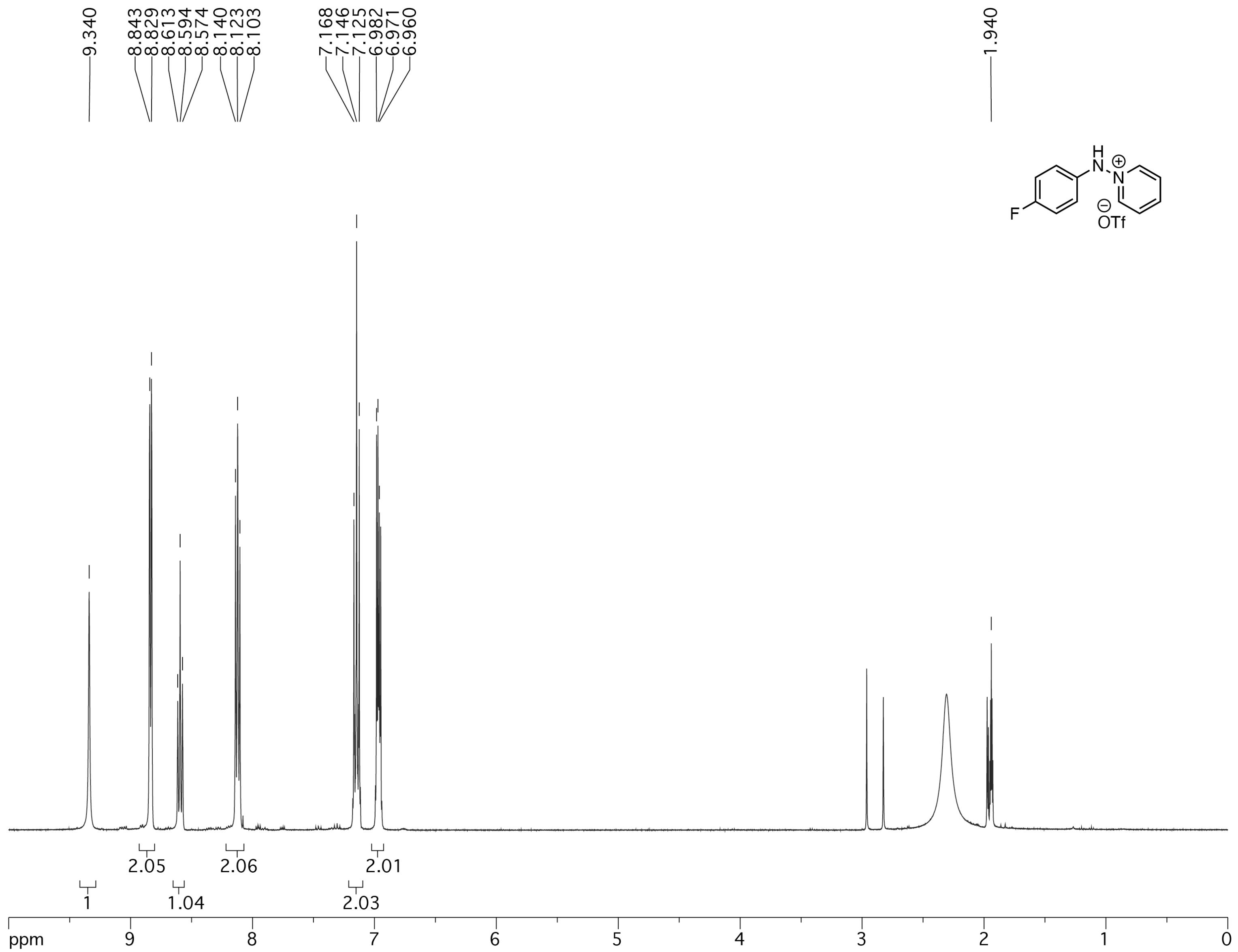

Figure S19. ^{1}H NMR spectrum of **3f** in CD_3CN (400 MHz) at 23 °C.

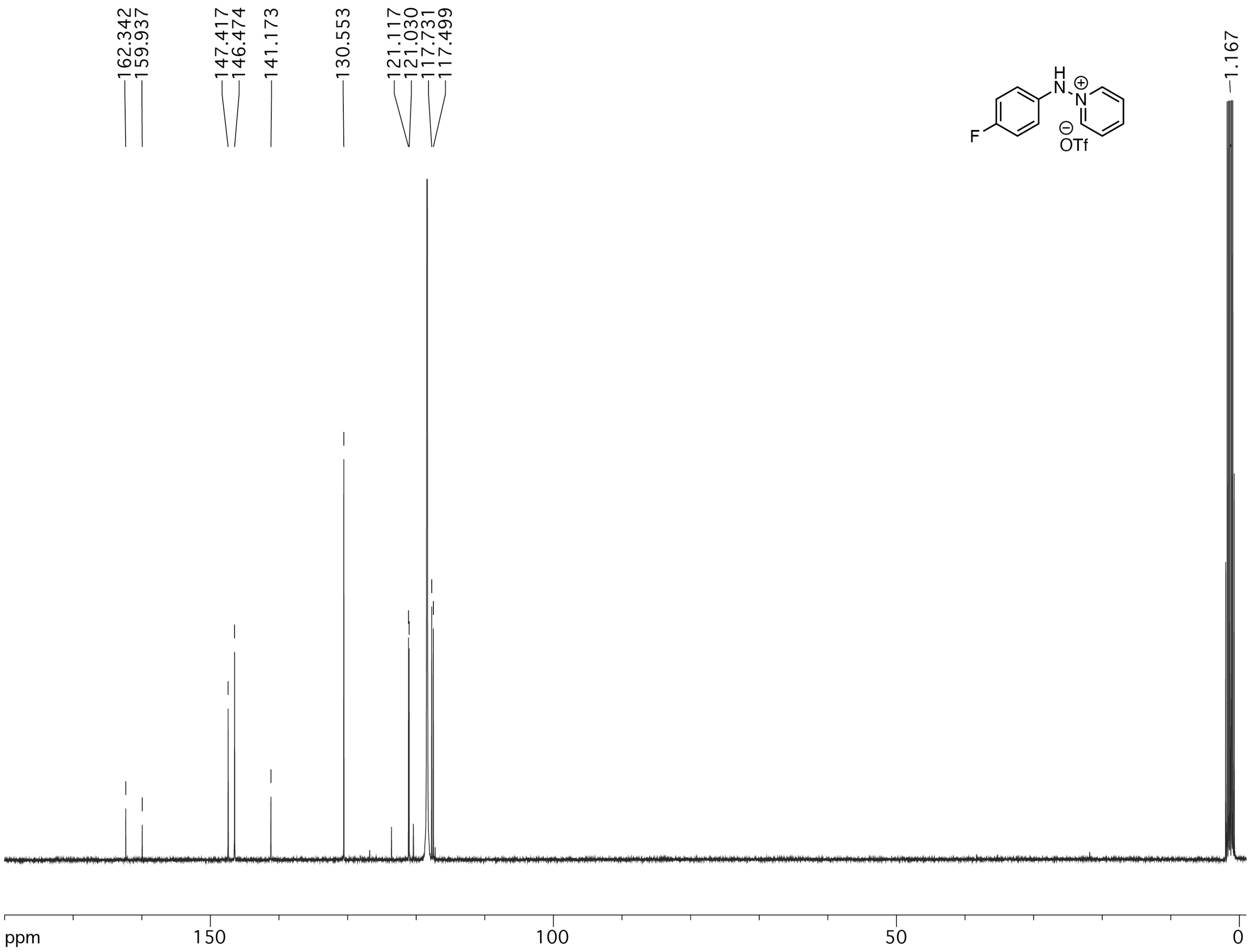

Figure S20. ^{13}C NMR spectrum of **3f** in CD_3CN (100 MHz) at 23 °C.

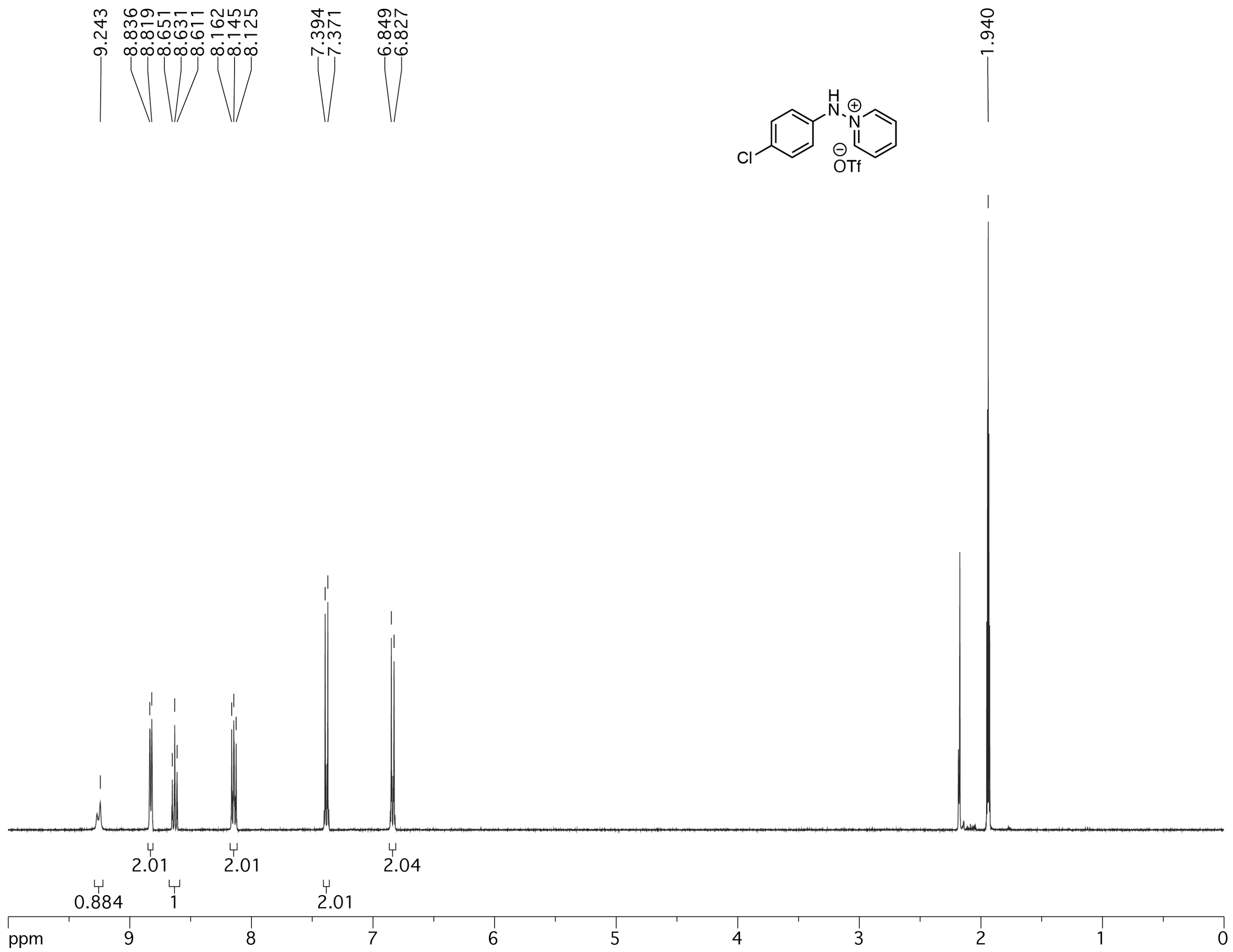

Figure S21. ^{1}H NMR spectrum of **3g** in CD_3CN (400 MHz) at 23 °C.

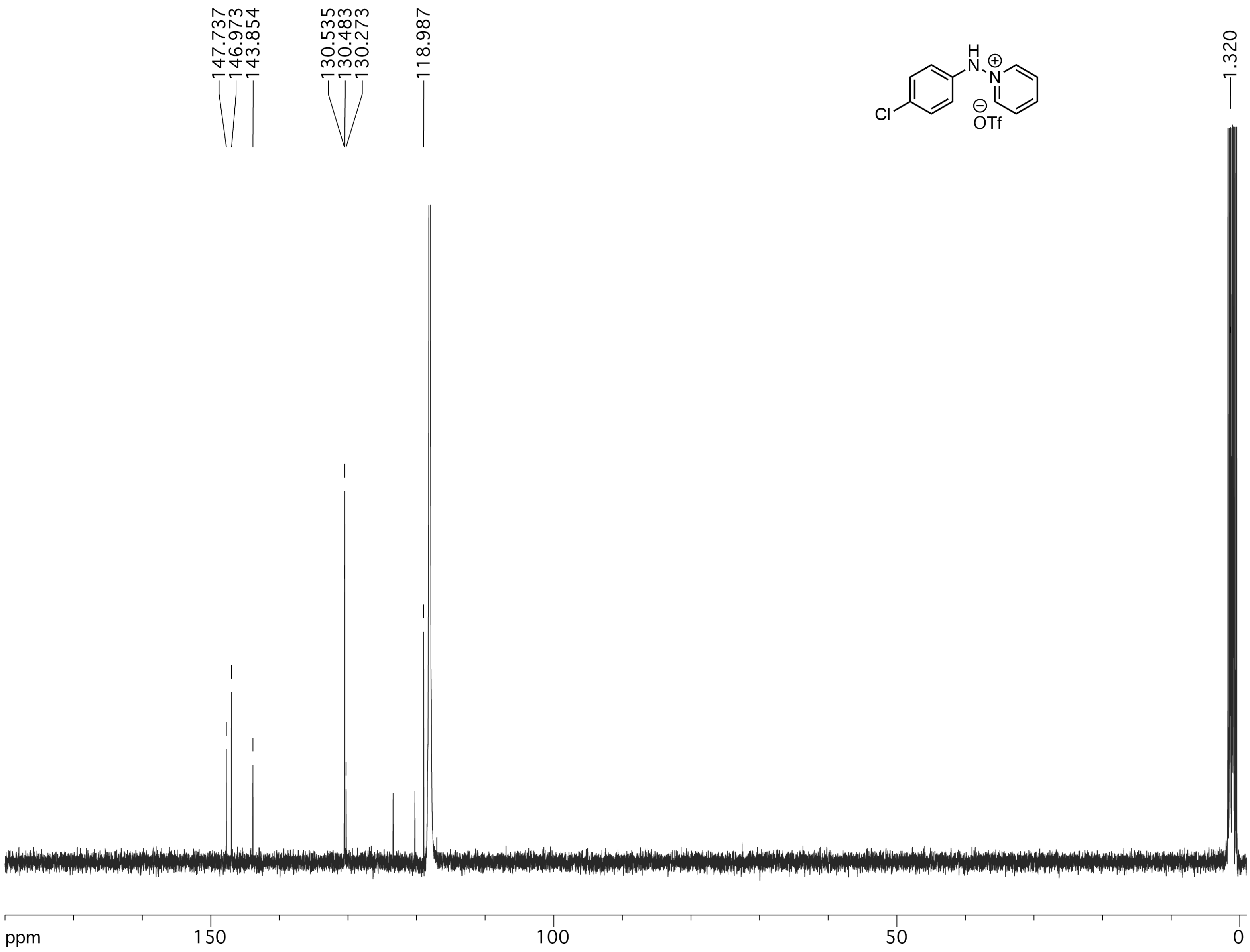

Figure S22. ^{13}C NMR spectrum of **3g** in CD_3CN (100 MHz) at 23 °C.

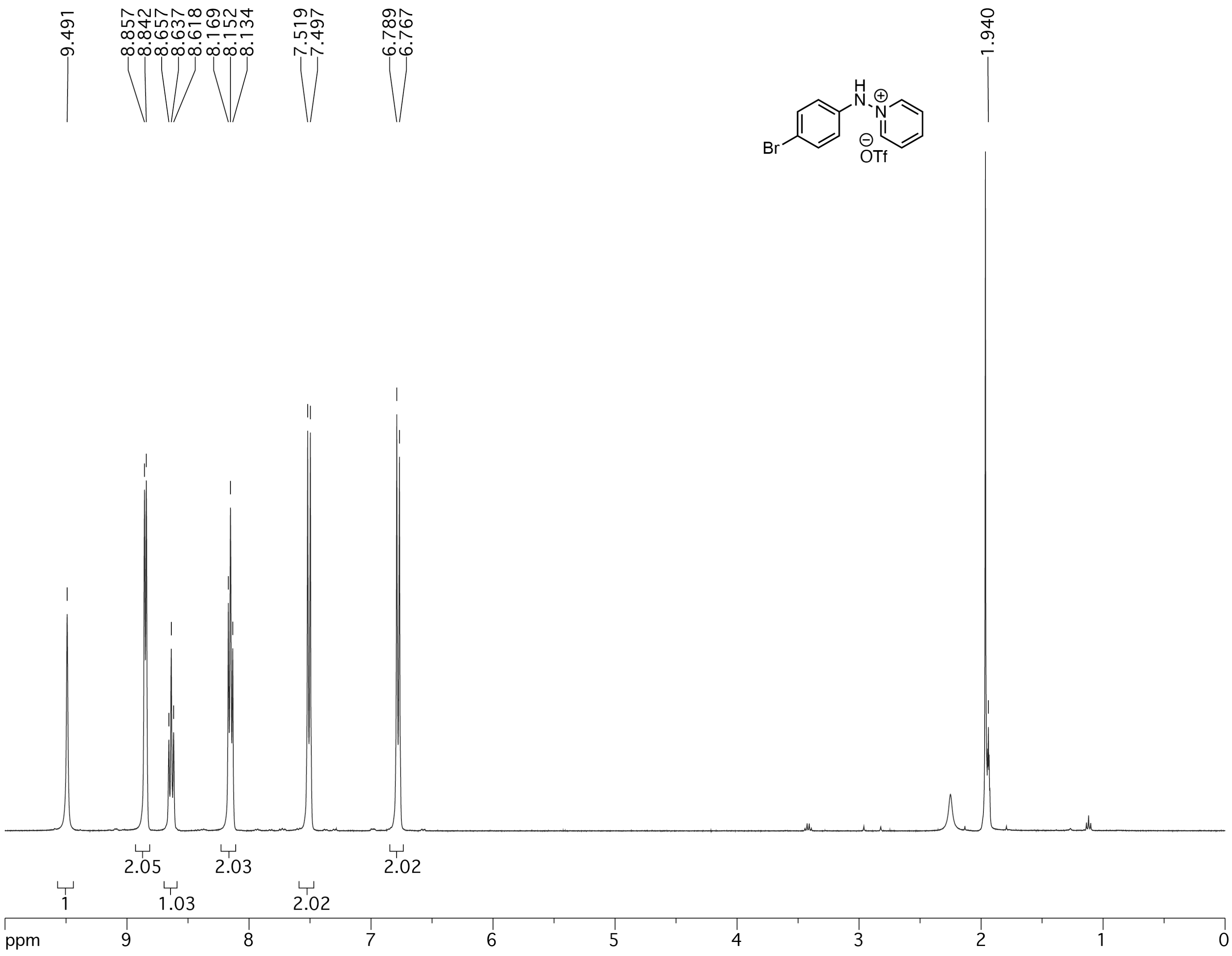

Figure S23. ^{1}H NMR spectrum of **3h** in CD_3CN (400 MHz) at 23 °C.

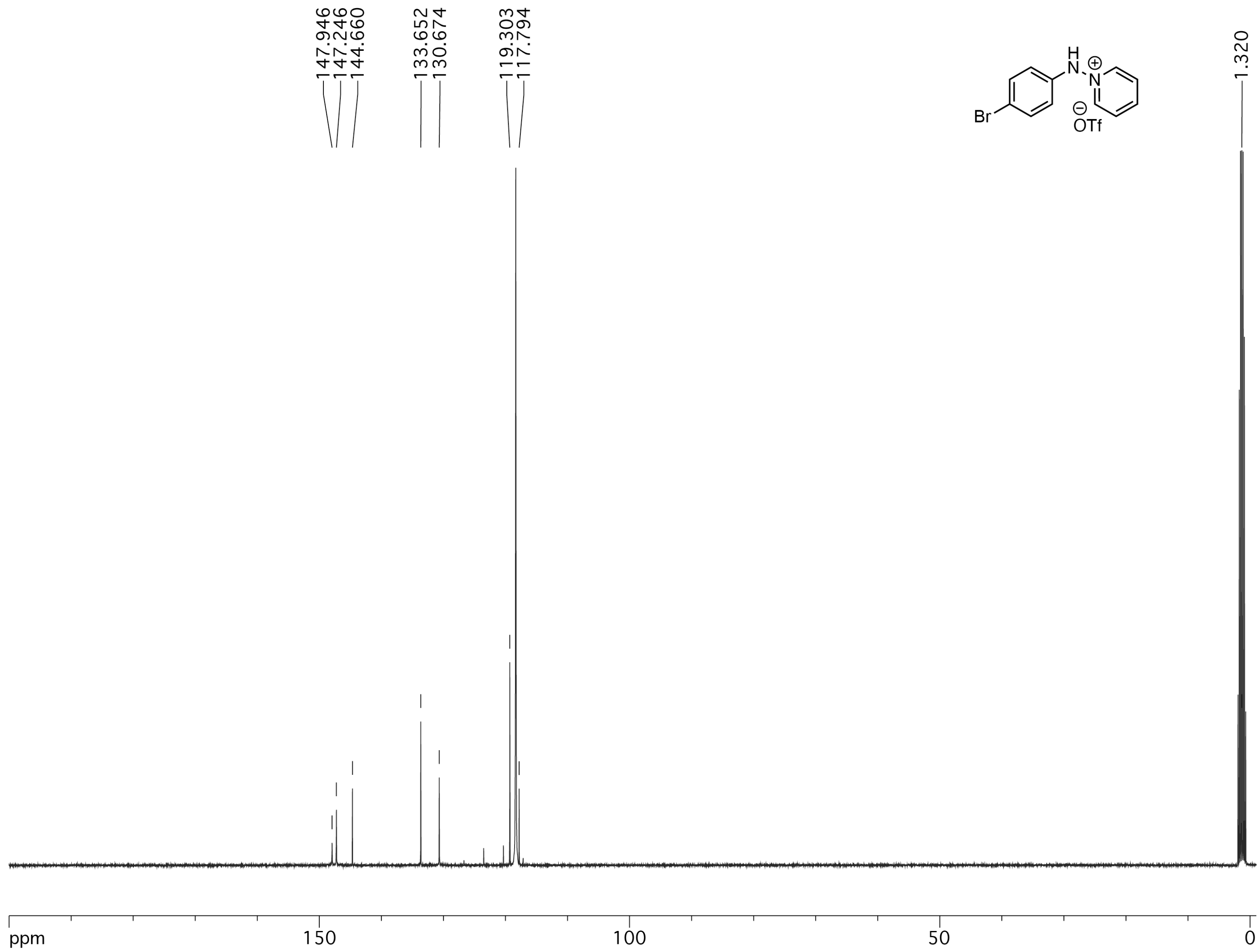

Figure S24. ^{13}C NMR spectrum of **3h** in CD_3CN (100 MHz) at 23 °C.

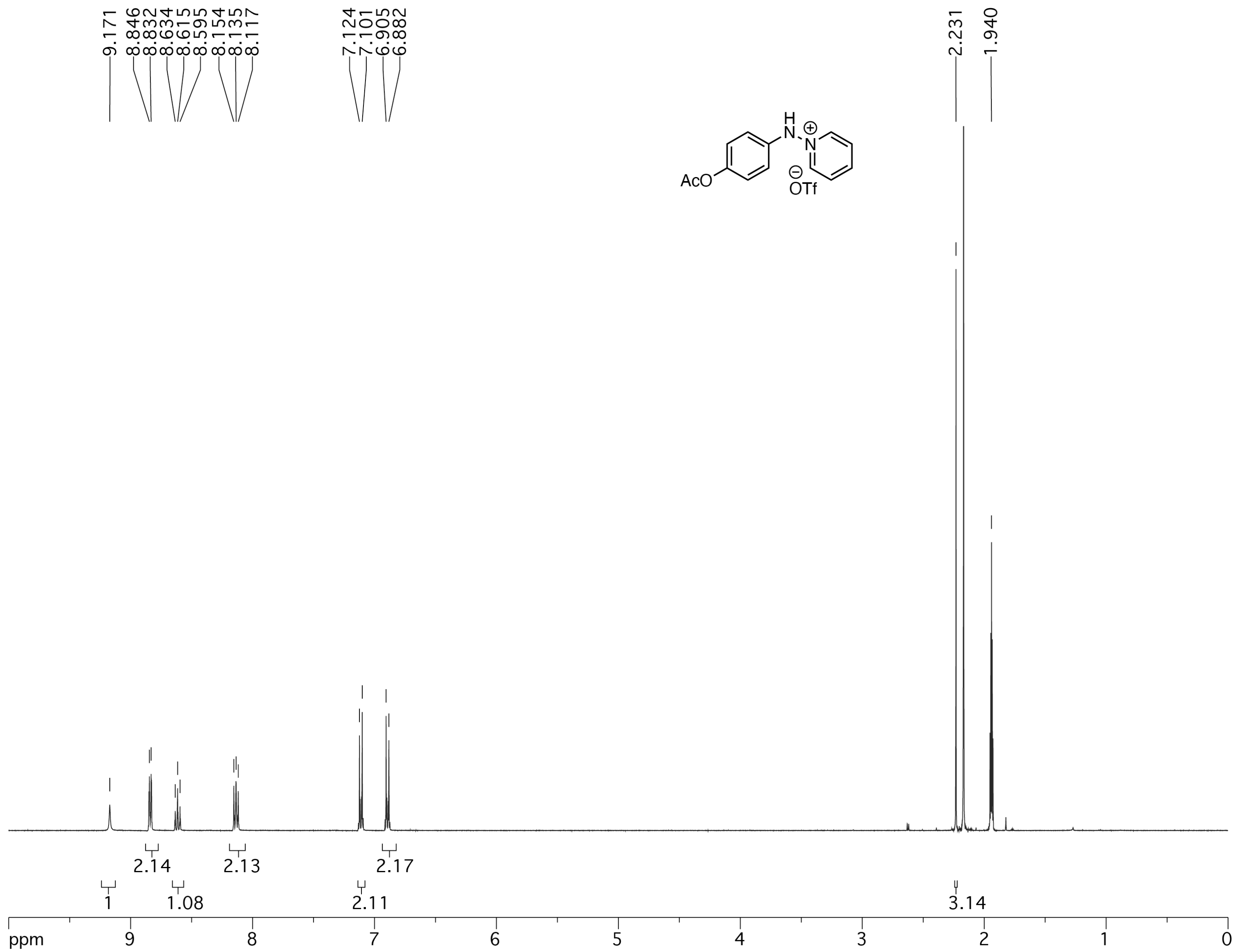

Figure S25. ^{1}H NMR spectrum of **3i** in CD_3CN (400 MHz) at 23 °C.

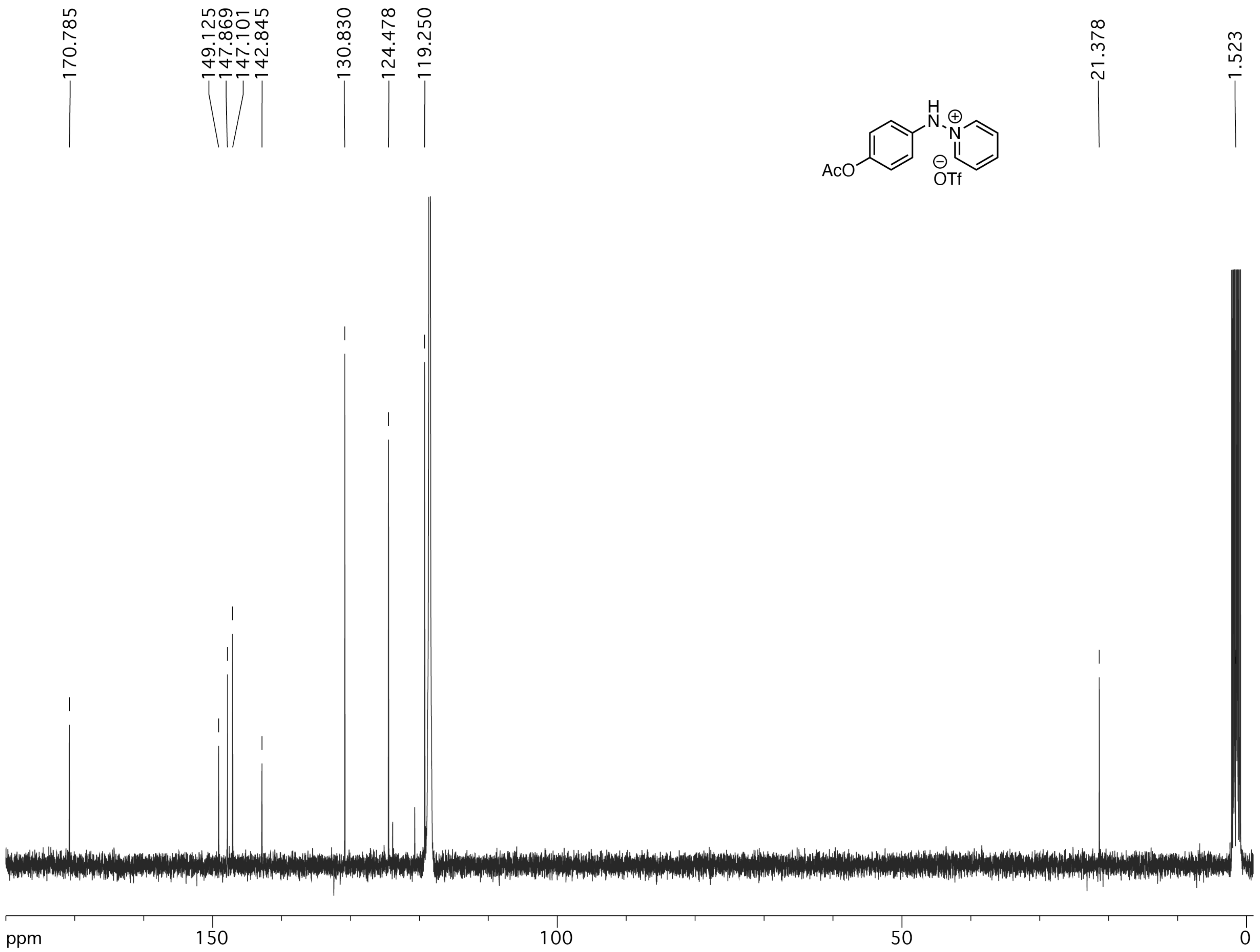

Figure S26. ^{13}C NMR spectrum of **3i** in CD_3CN (100 MHz) at 23 °C.

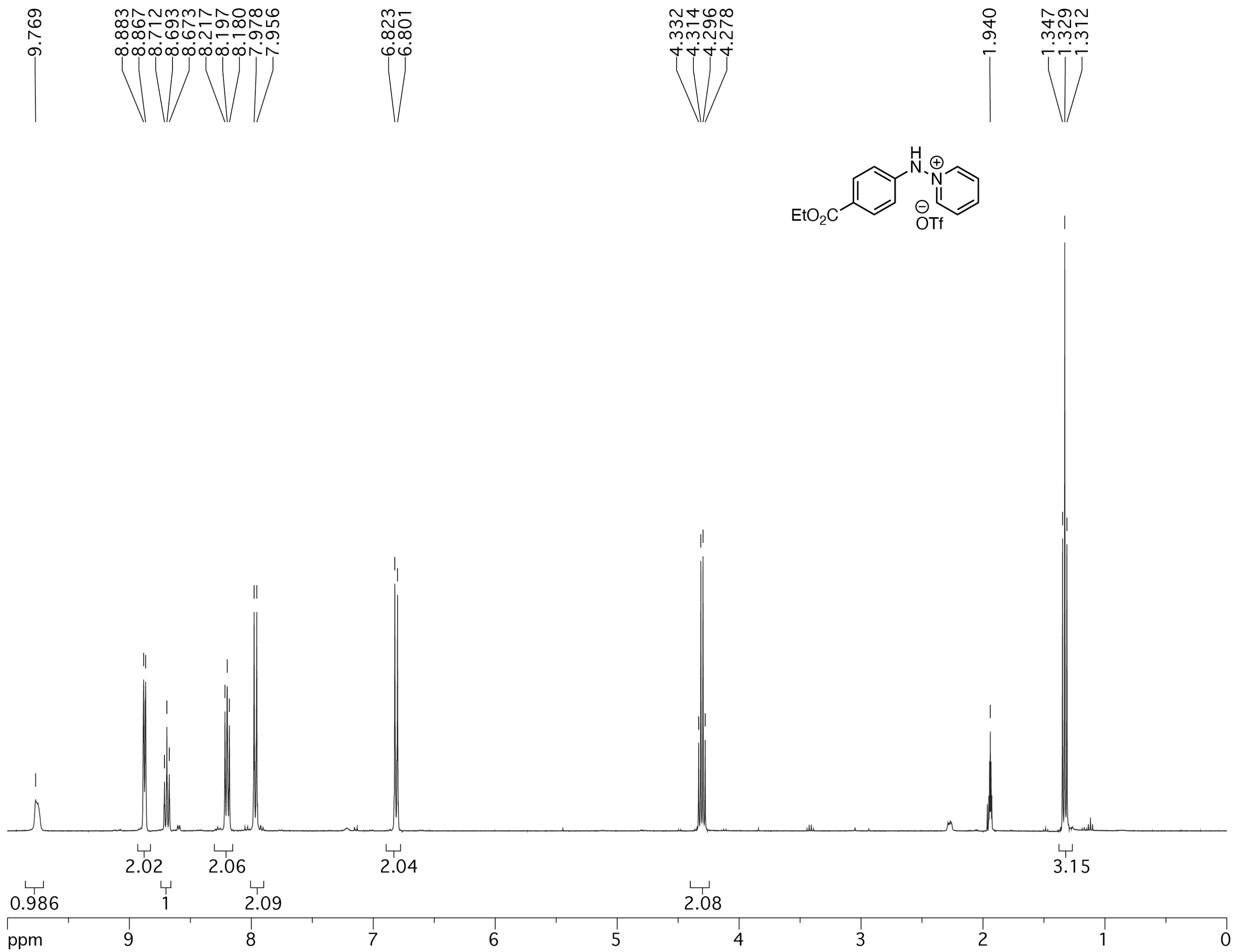

Figure S27. ^{1}H NMR spectrum of **3j** in CD_3CN (400 MHz) at 23 °C.

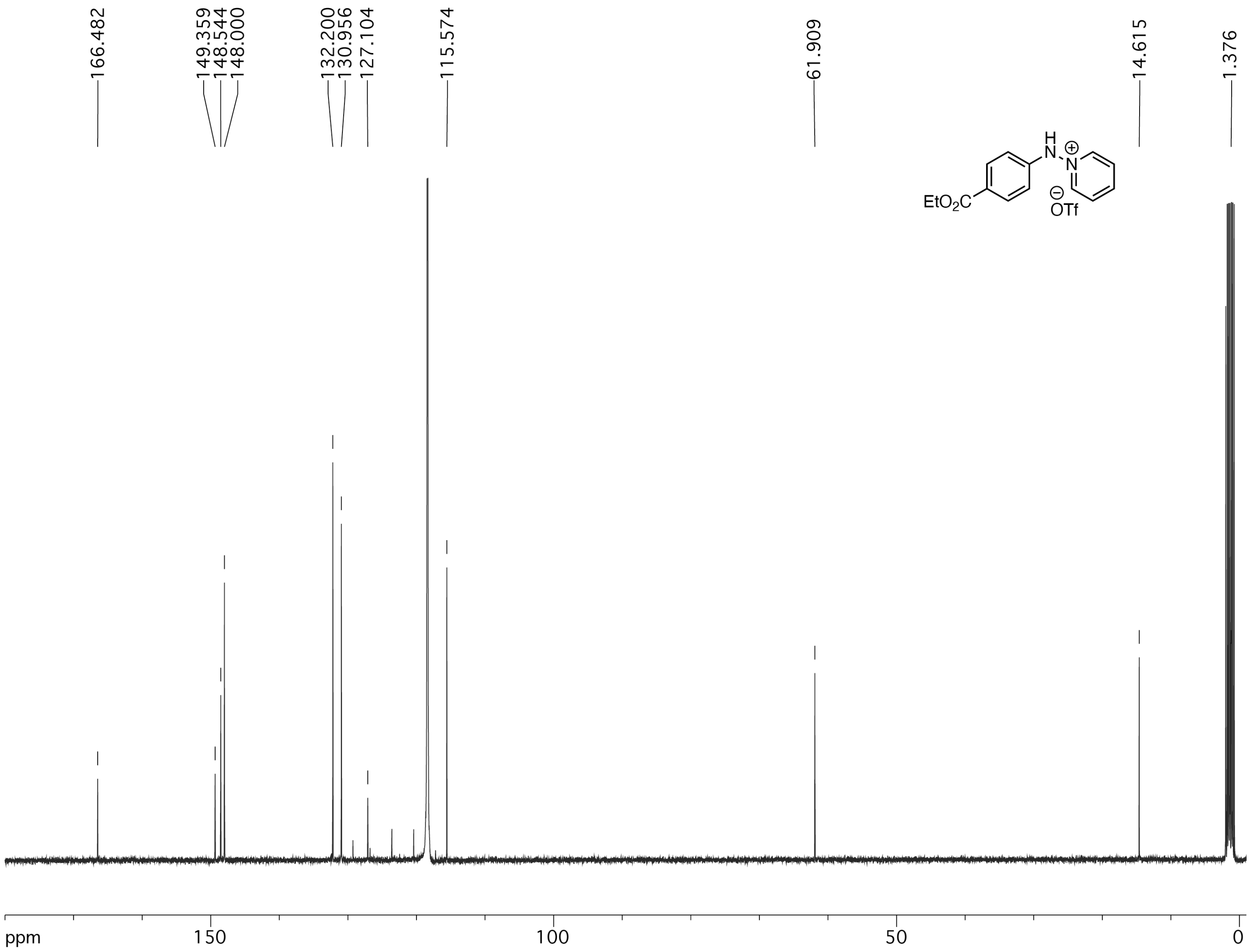

Figure S28. ^{13}C NMR spectrum of **3j** in CD_3CN (100 MHz) at 23 °C.

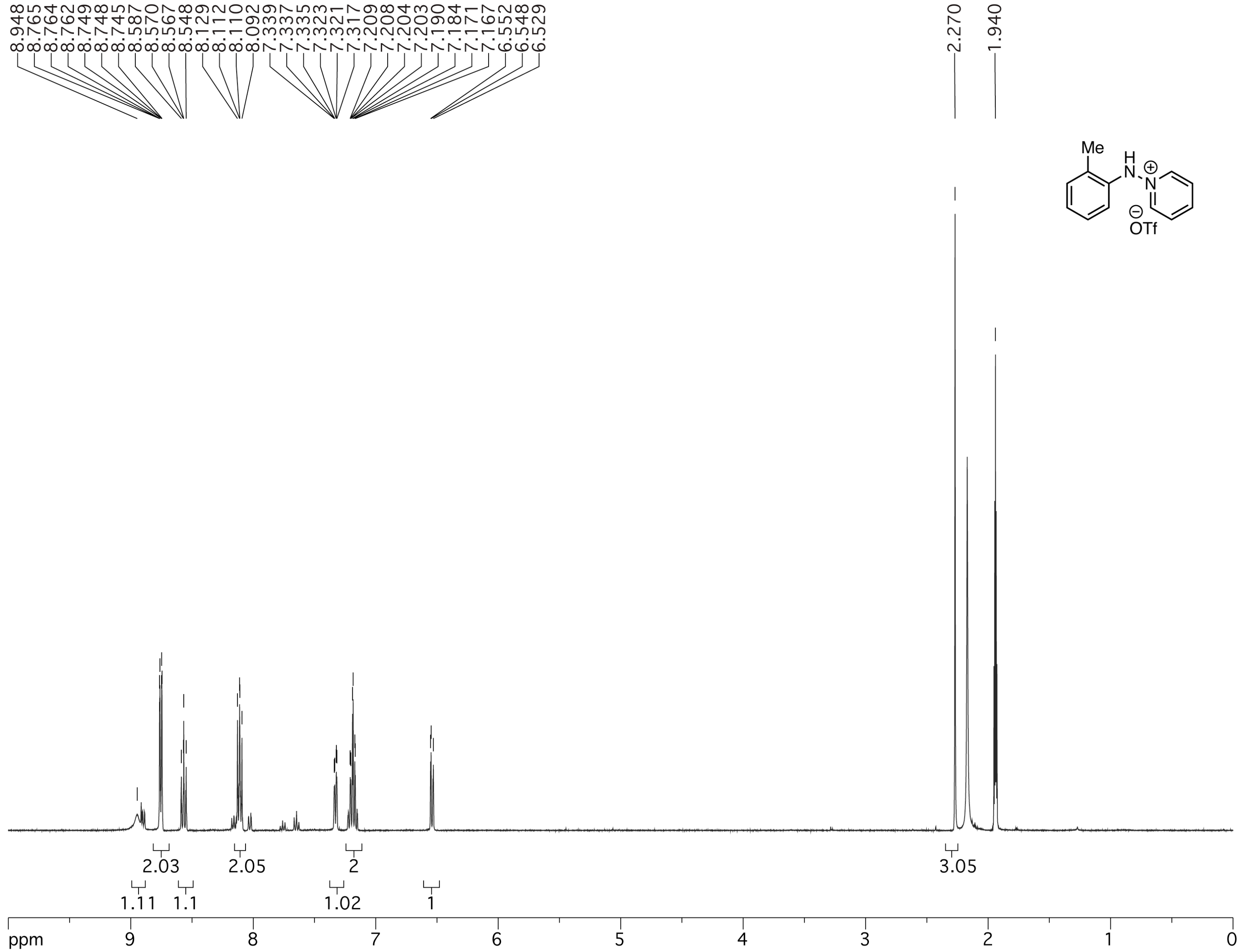

Figure S29. ^{1}H NMR spectrum of **3k** in CD_3CN (400 MHz) at 23 °C.

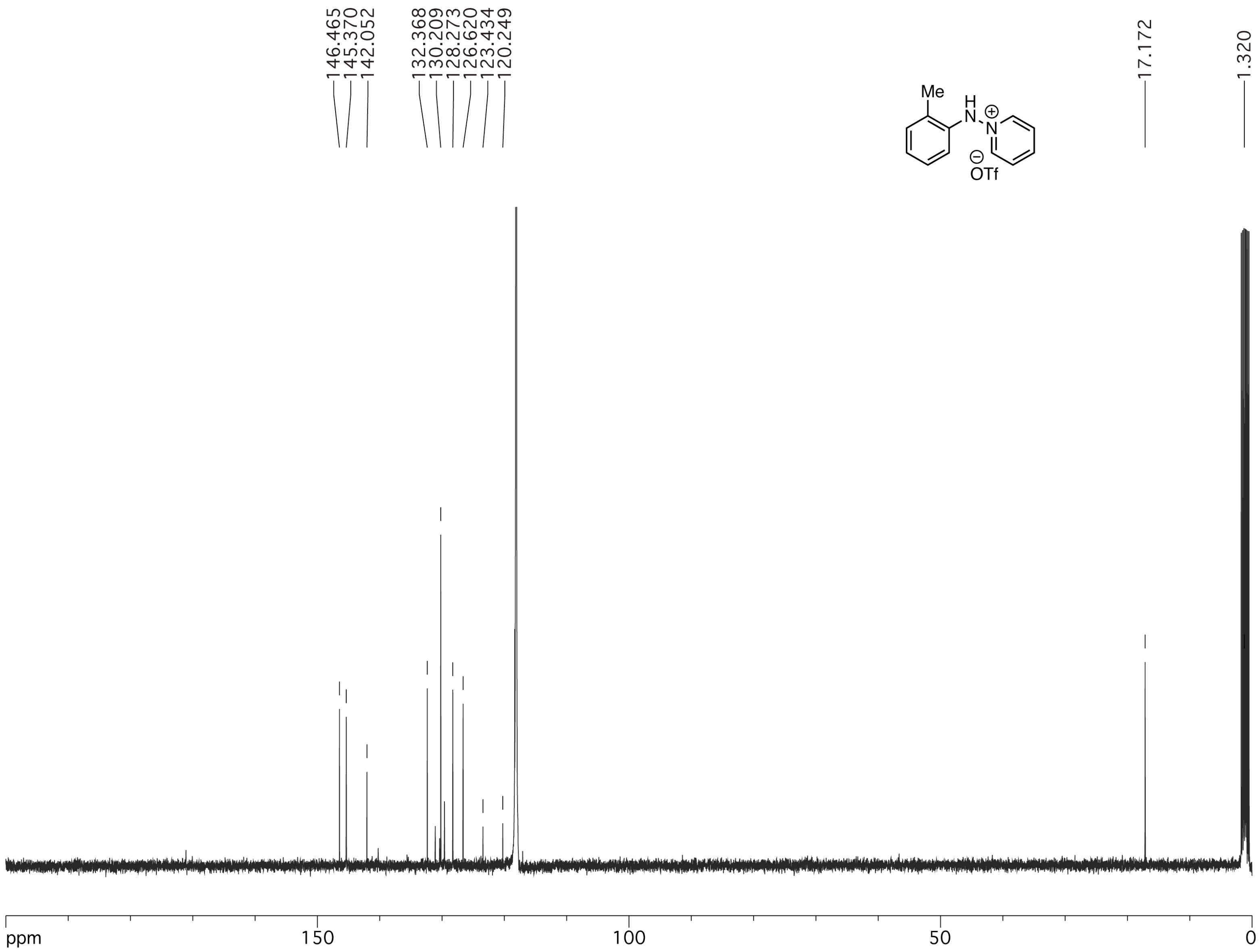

Figure S30. ^{13}C NMR spectrum of **3k** in CD_3CN (100 MHz) at 23 °C.

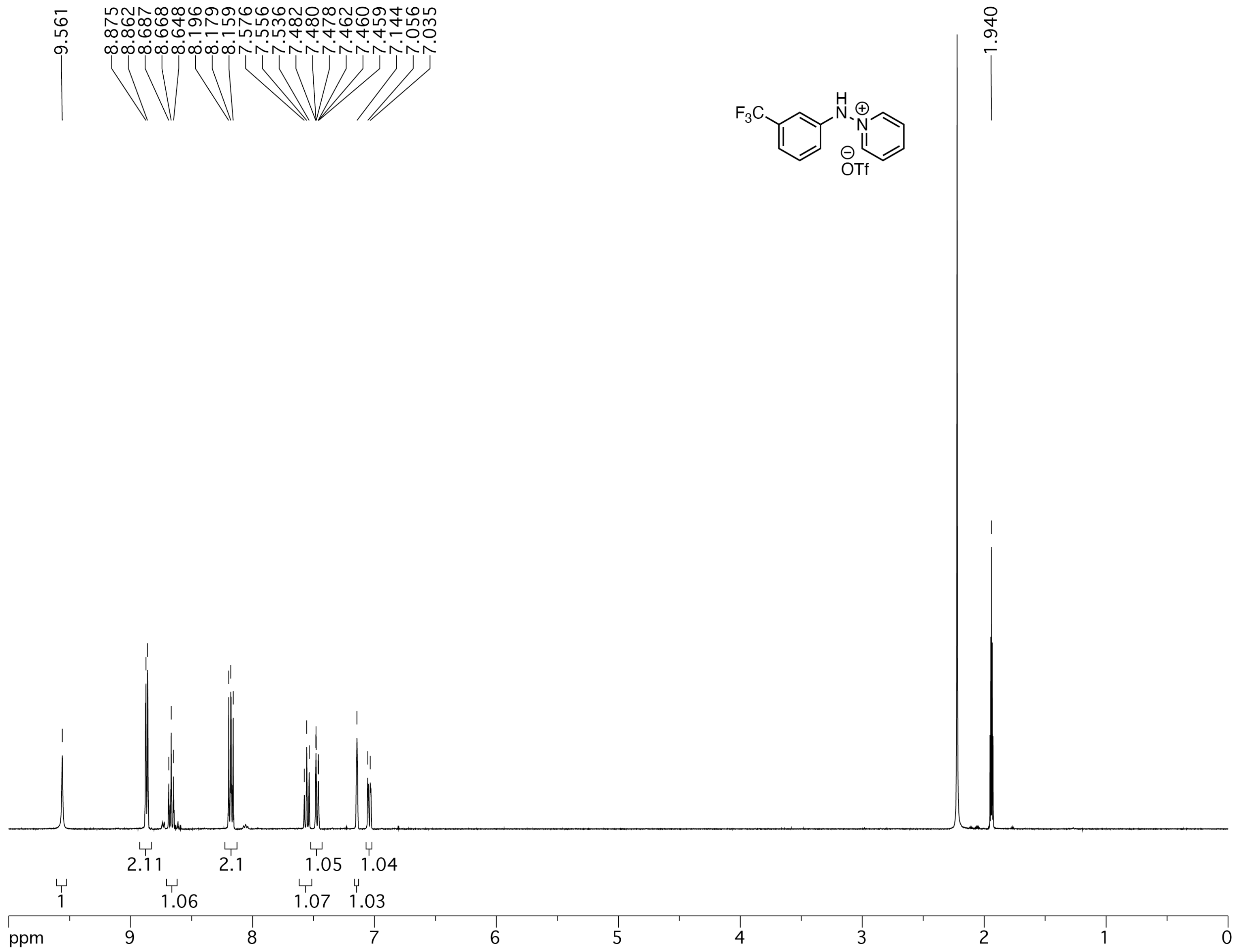

Figure S31. ^{1}H NMR spectrum of **3l** in CD_3CN (400 MHz) at 23 °C.

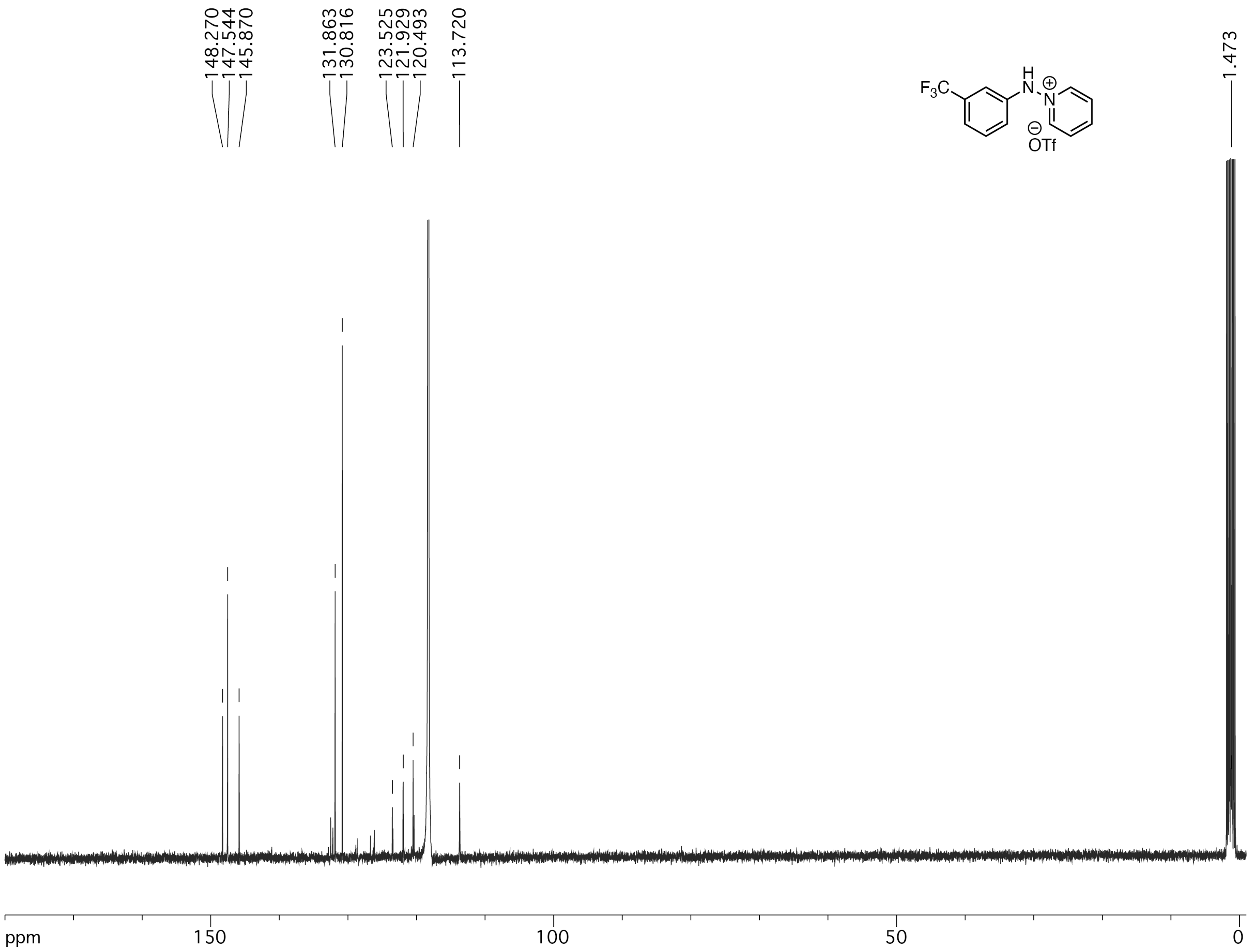

Figure S32. ^{13}C NMR spectrum of **3l** in CD_3CN (100 MHz) at 23 °C.

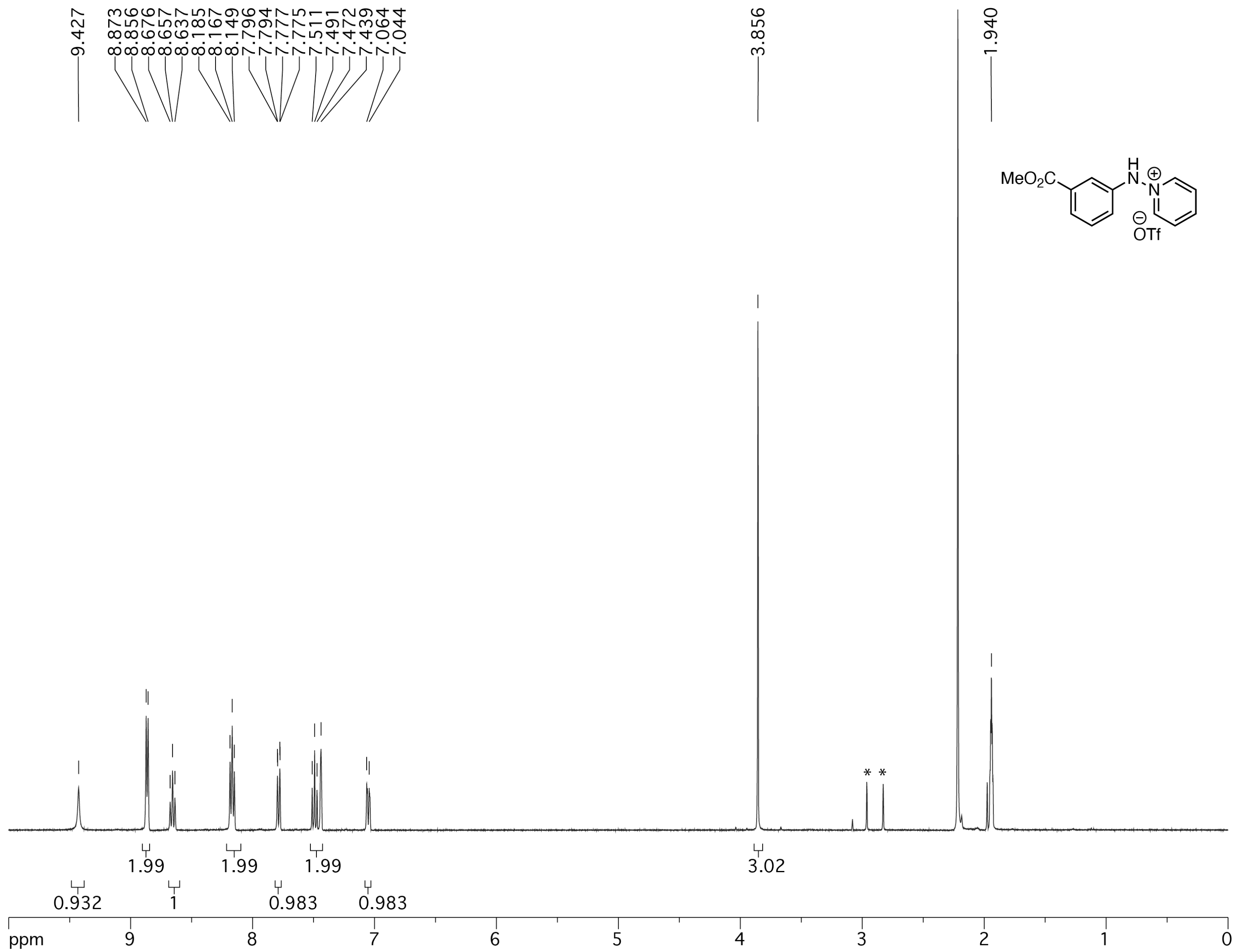

Figure S33. ^{1}H NMR spectrum of **3m** in CD_3CN (400 MHz) at 23 °C. Residual DMA peaks are marked with *.

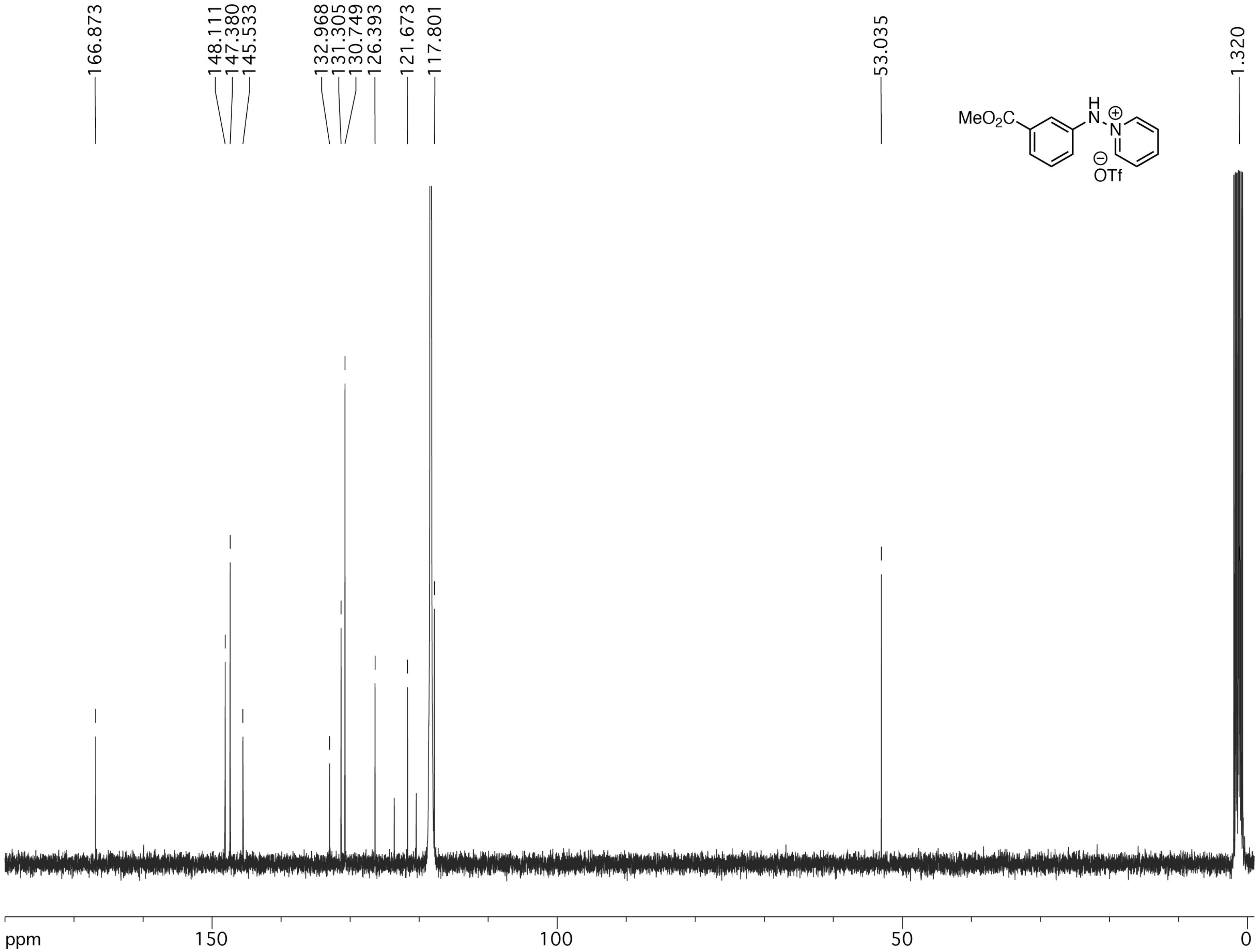

Figure S34. ^{13}C NMR spectrum of **3m** in CD_3CN (100 MHz) at 23 °C.

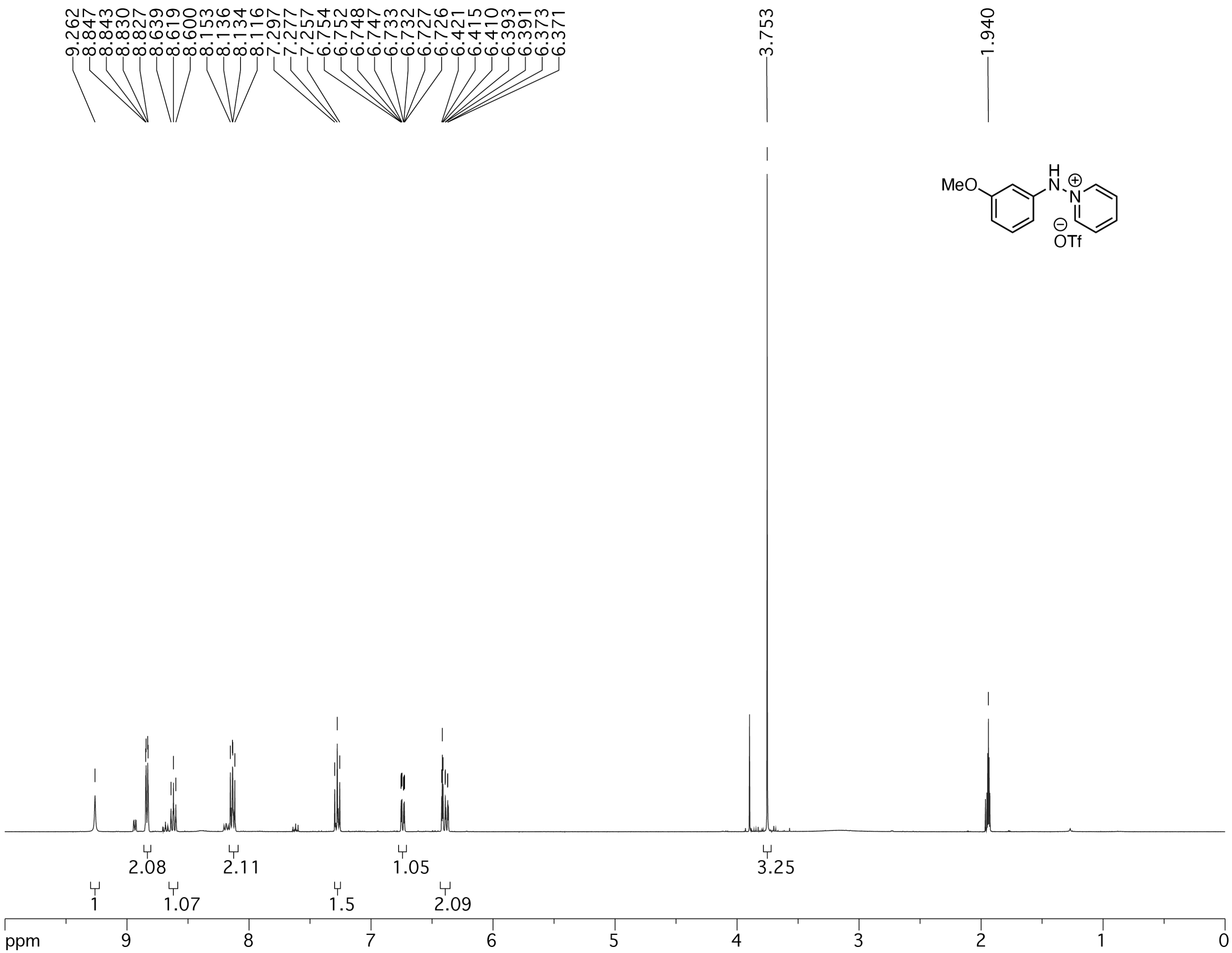

Figure S35. ^{1}H NMR spectrum of **3n** in CD_3CN (400 MHz) at 23 °C.

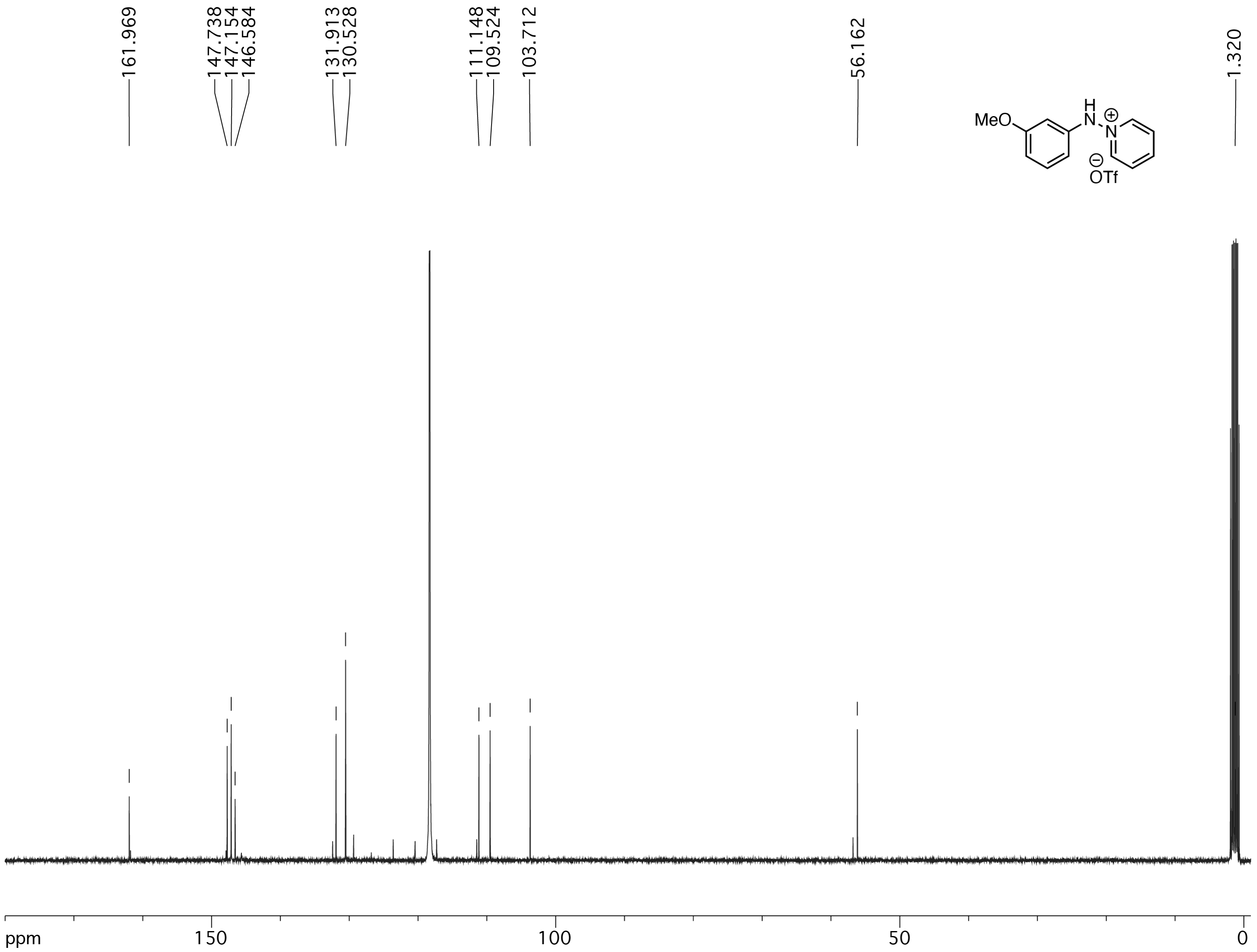

Figure S36. ^{13}C NMR spectrum of **3n** in CD_3CN (100 MHz) at 23 °C.

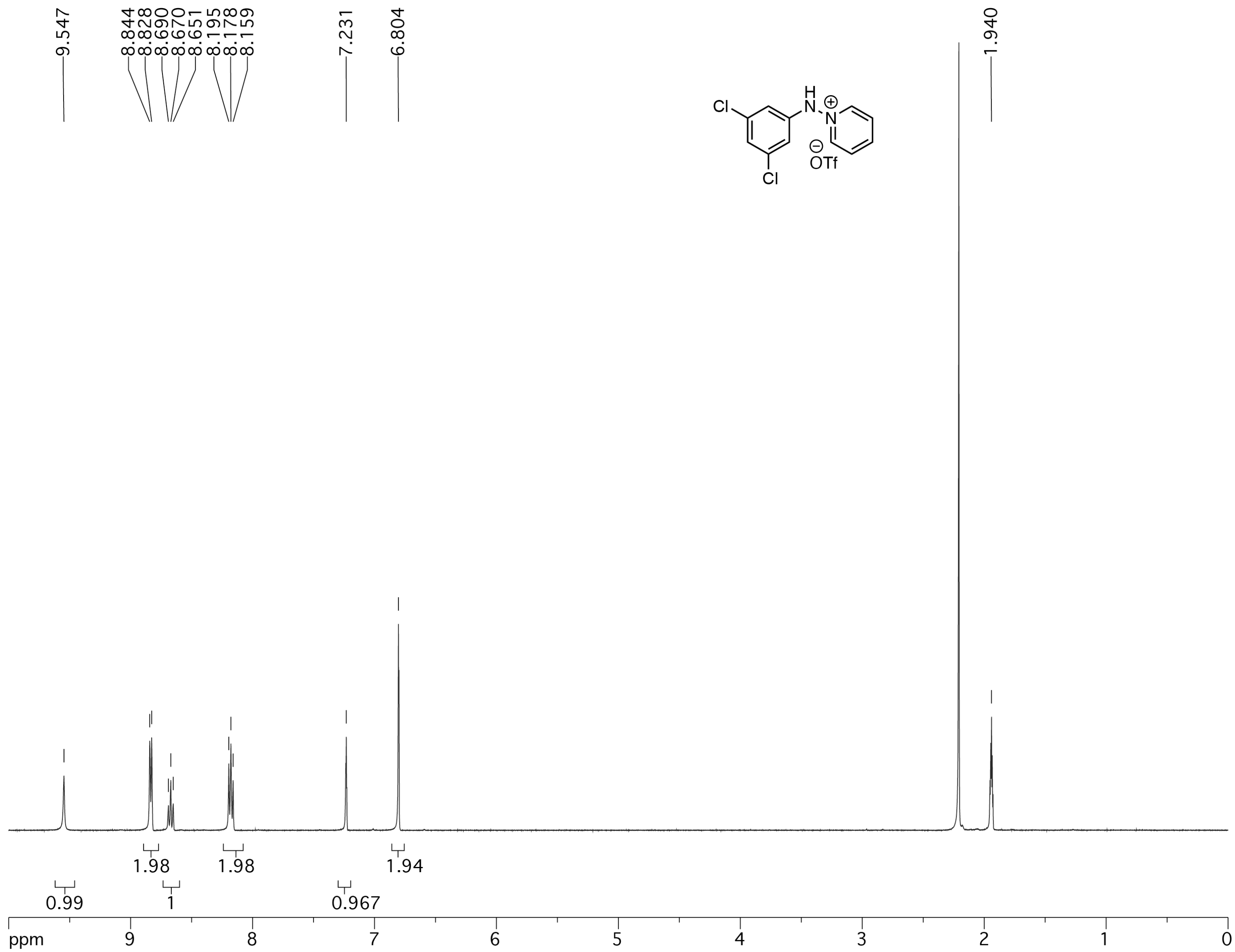

Figure S37. ^{1}H NMR spectrum of **3o** in CD_3CN (400 MHz) at 23 °C.

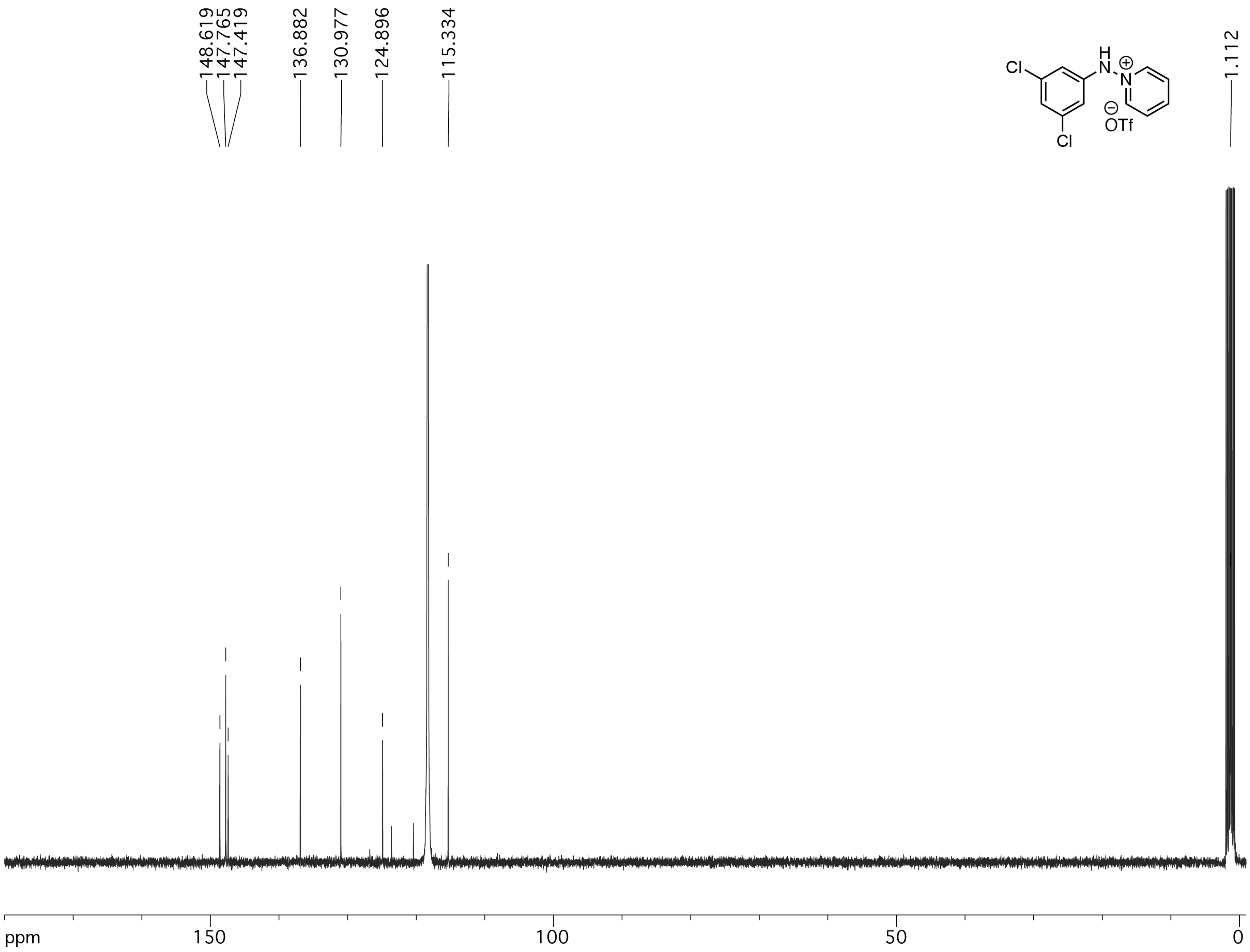

Figure S38. ^{13}C NMR spectrum of **3o** in CD_3CN (100 MHz) at 23 °C.

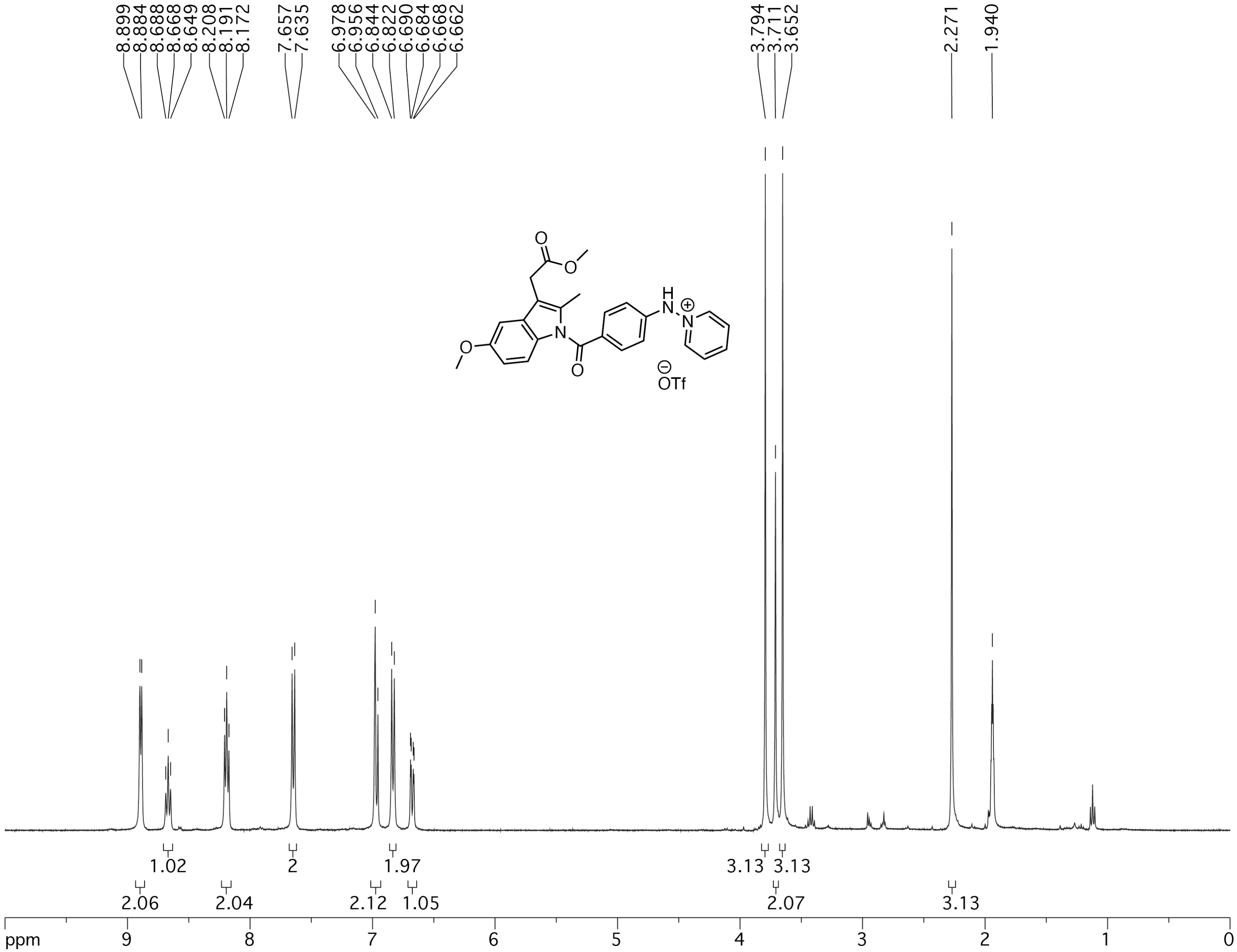

Figure S39. ^{1}H NMR spectrum of **3p** in CD_3CN (400 MHz) at 23 °C.

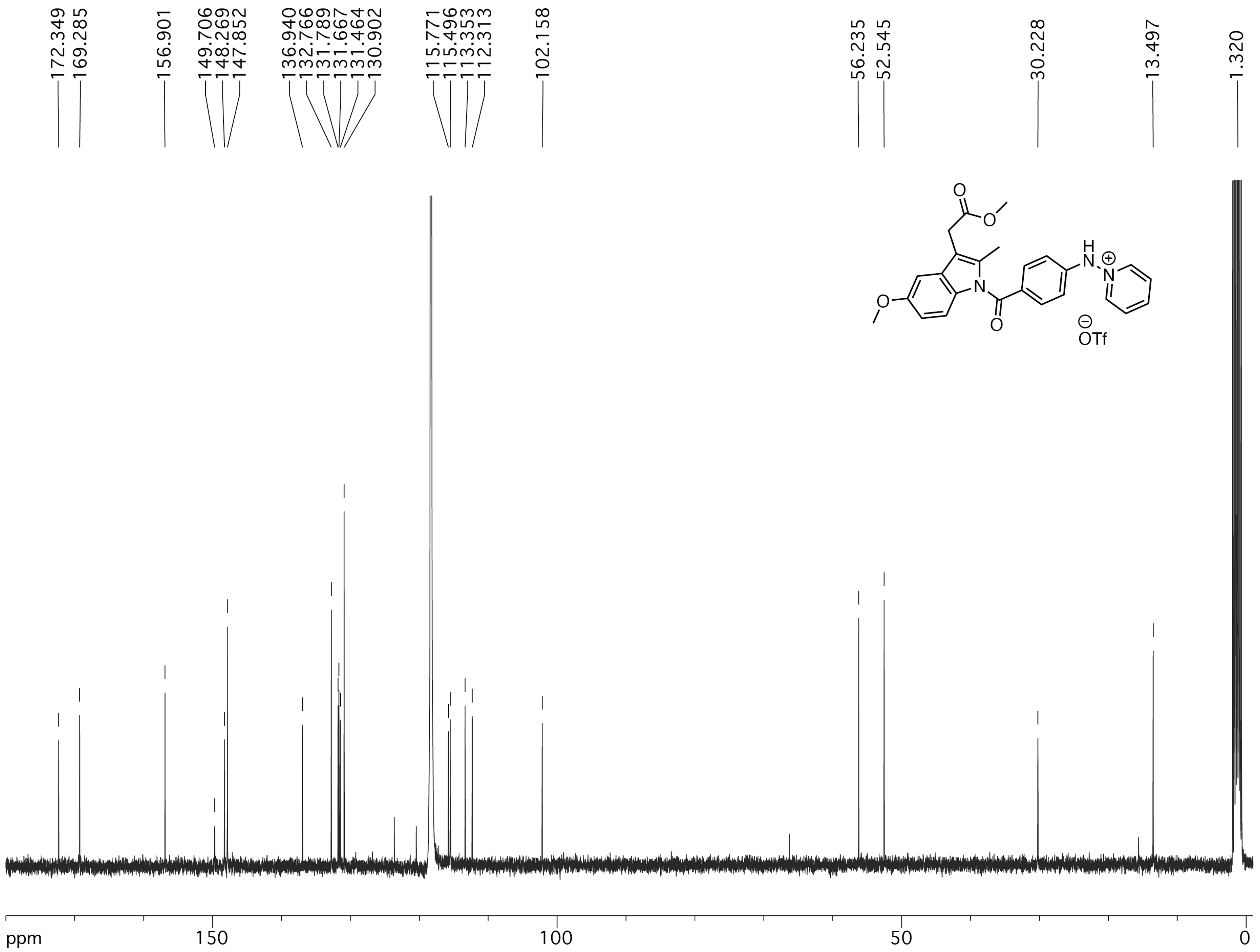

Figure S40. ^{13}C NMR spectrum of **3p** in CD_3CN (100 MHz) at 23 °C.

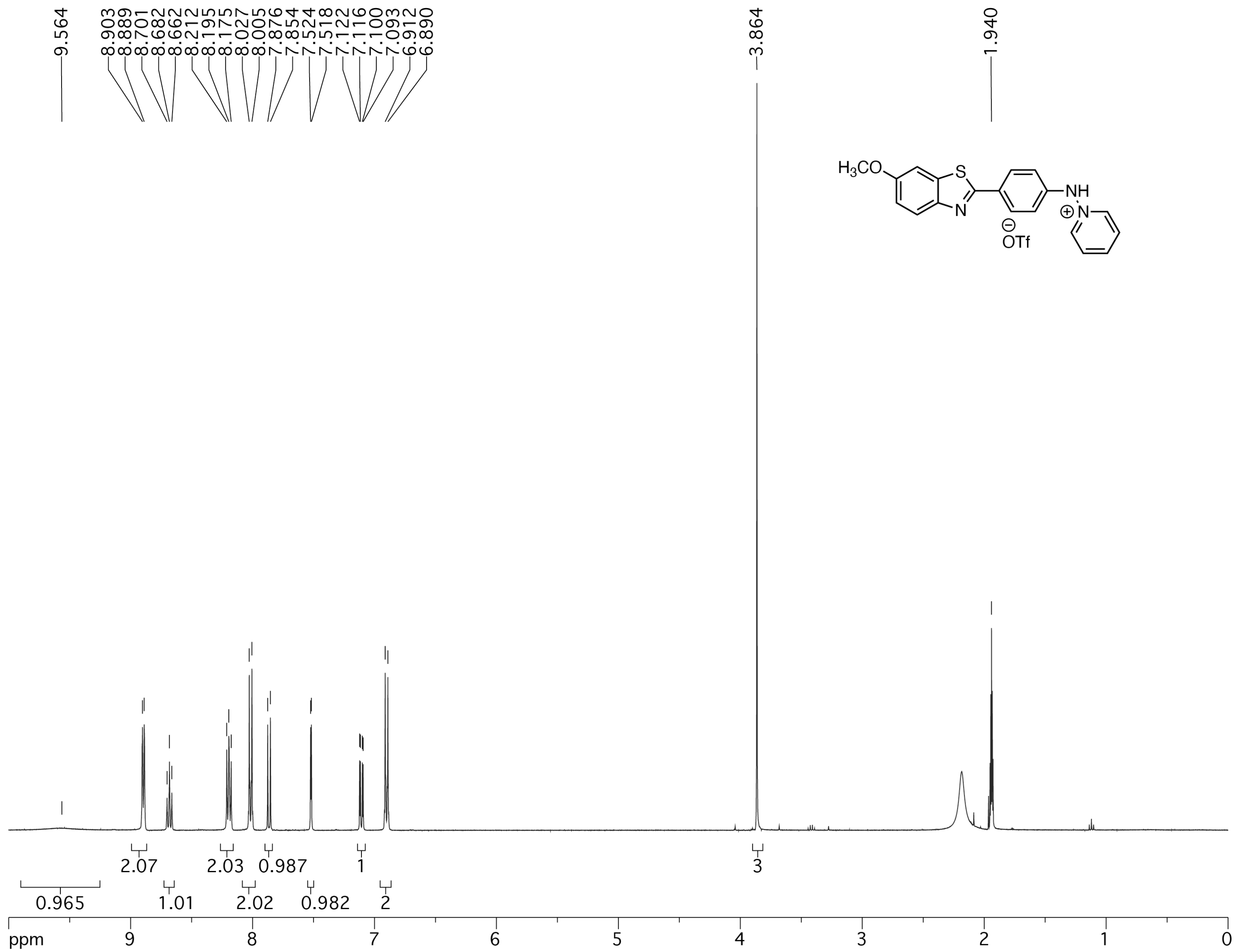

Figure S41. ^{1}H NMR spectrum of **3q** in CD_3CN (400 MHz) at 23 °C.

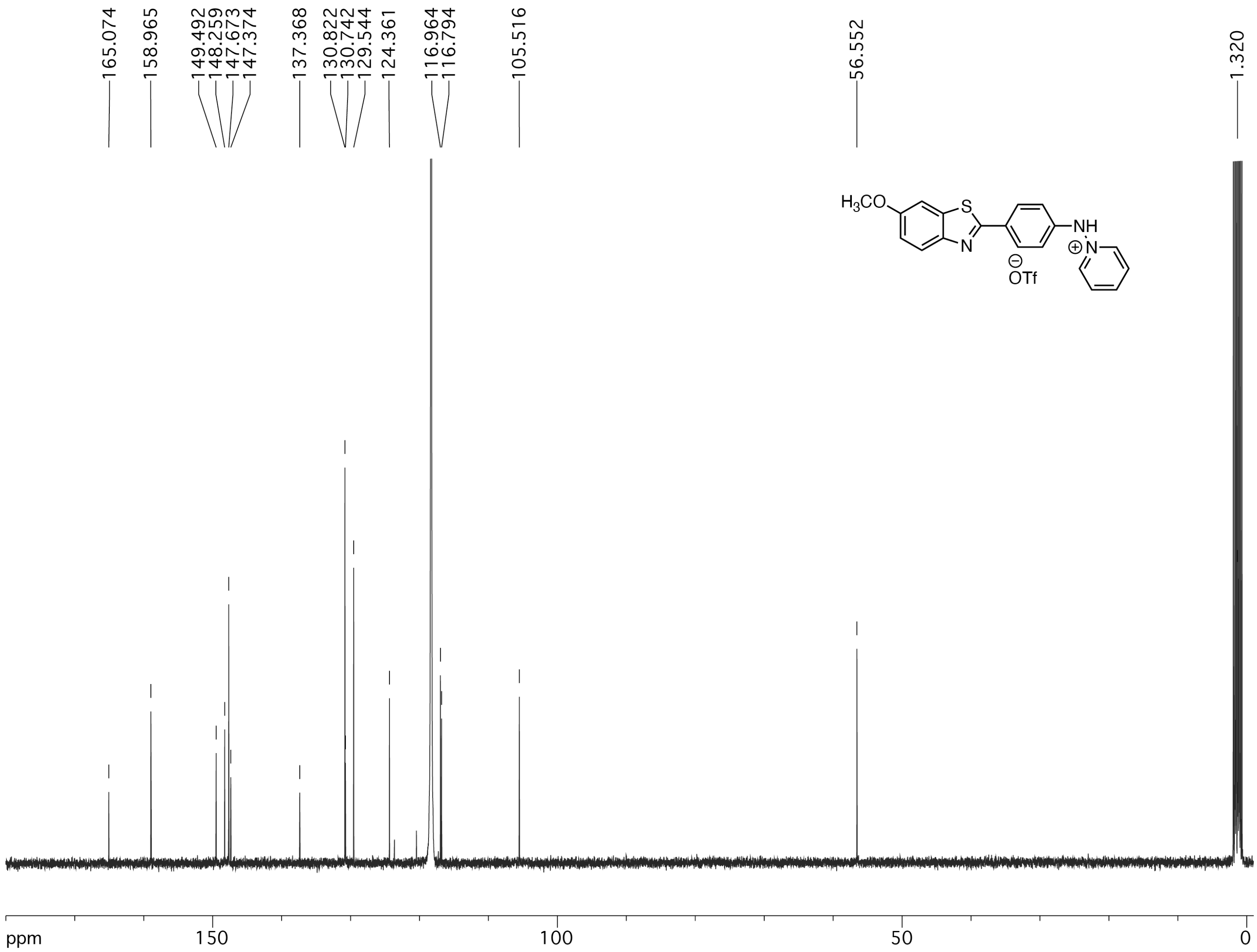

Figure S42. ${}^{13}C$ NMR spectrum of **3q** in CD_3CN (100 MHz) at 23 °C.

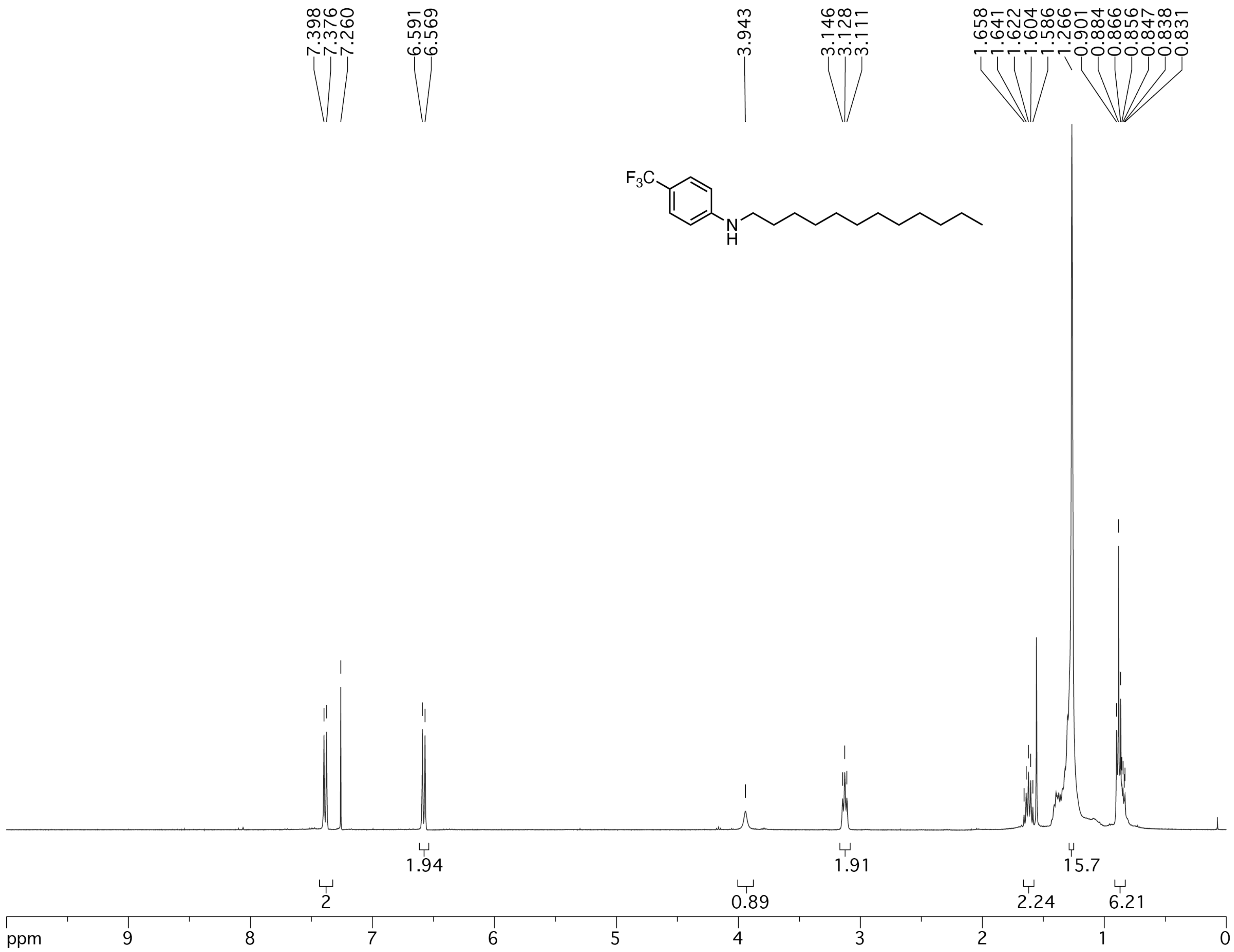

Figure S43. ^{1}H NMR spectrum of **5b** in $CDCl_3$ (400 MHz) at 23 °C.

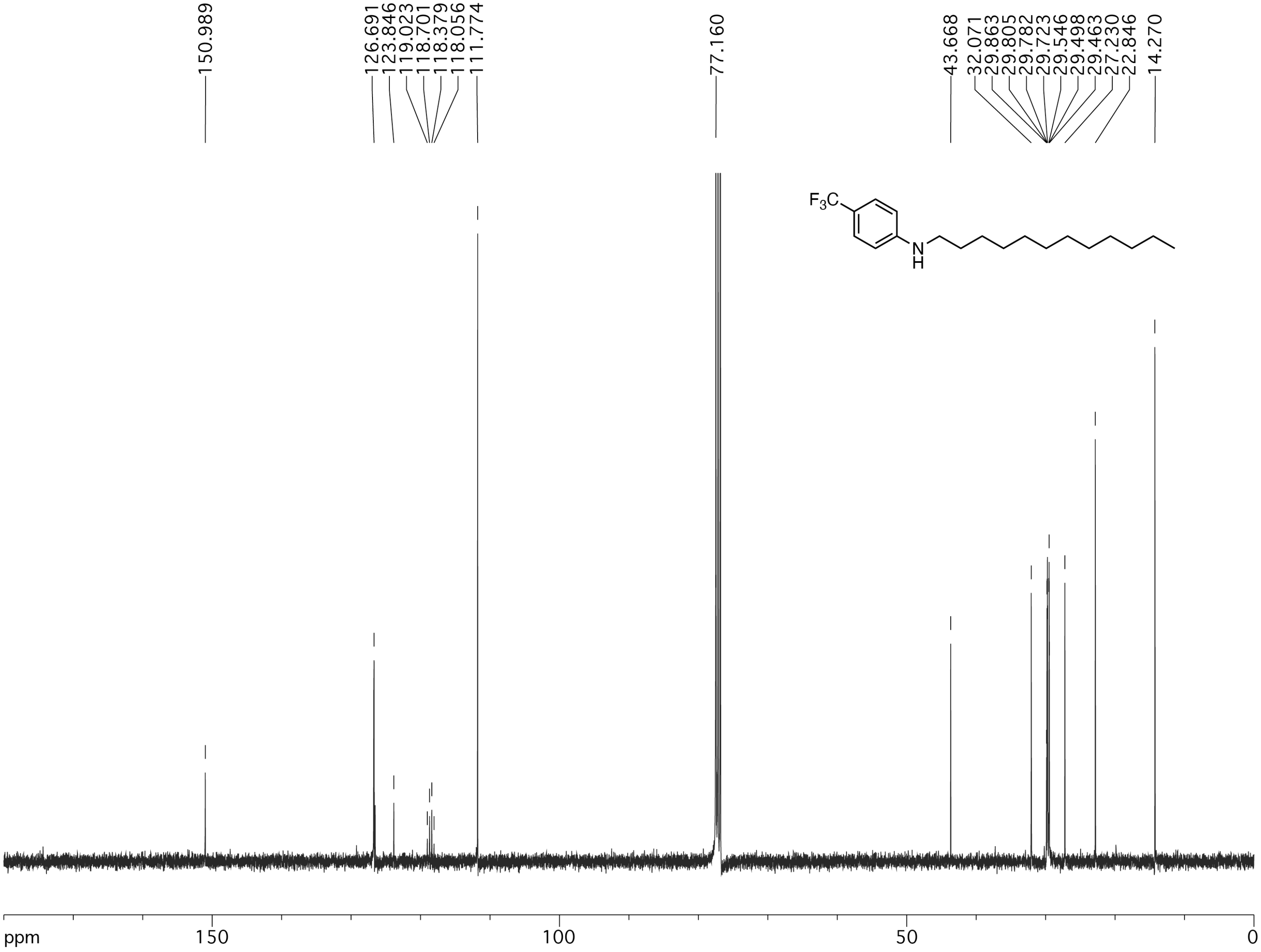

Figure S44. ^{13}C NMR spectrum of **5b** in $CDCl_3$ (100 MHz) at 23 °C.

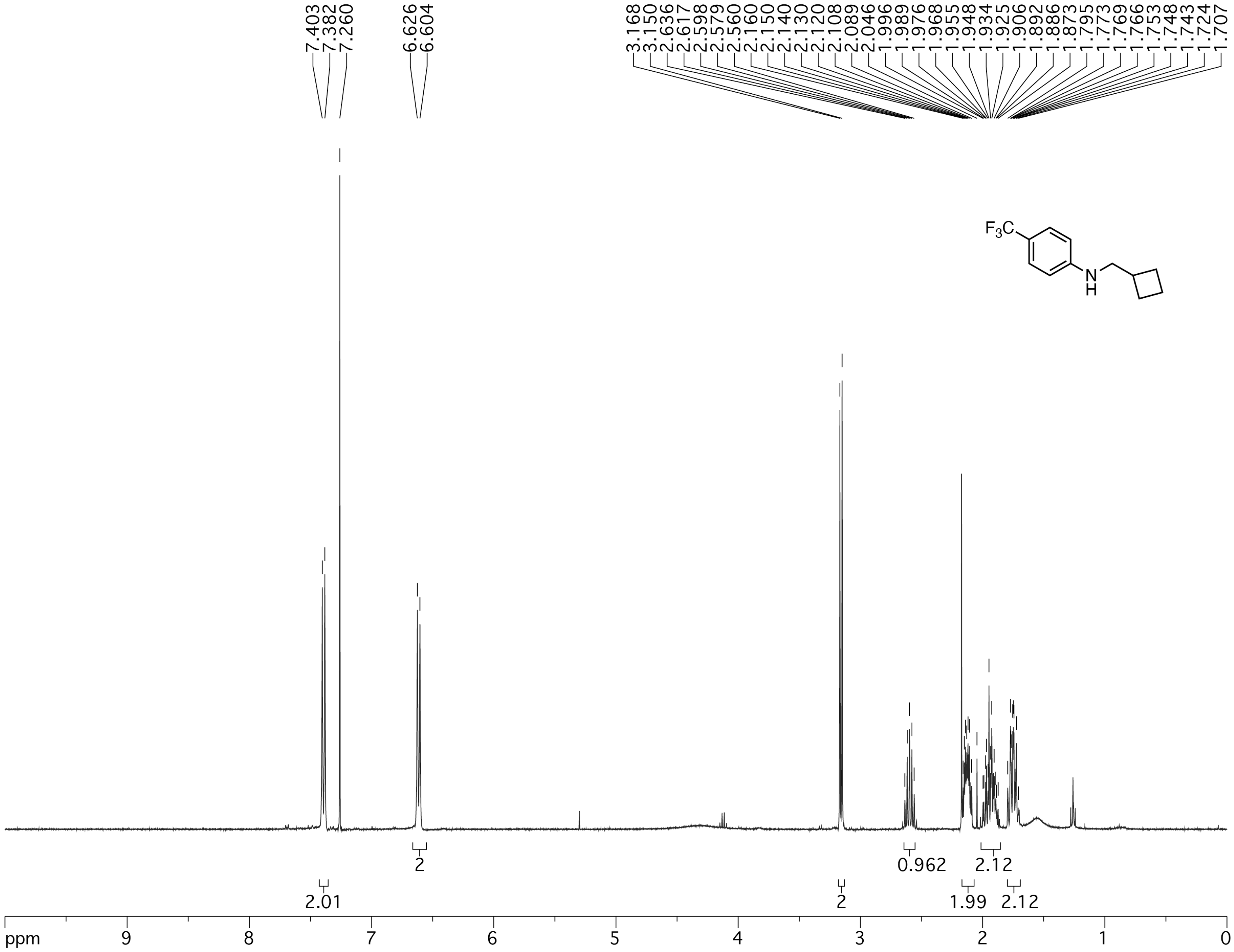

Figure S45. ^{1}H NMR spectrum of **5d** in $CDCl_3$ (400 MHz) at 23 °C.

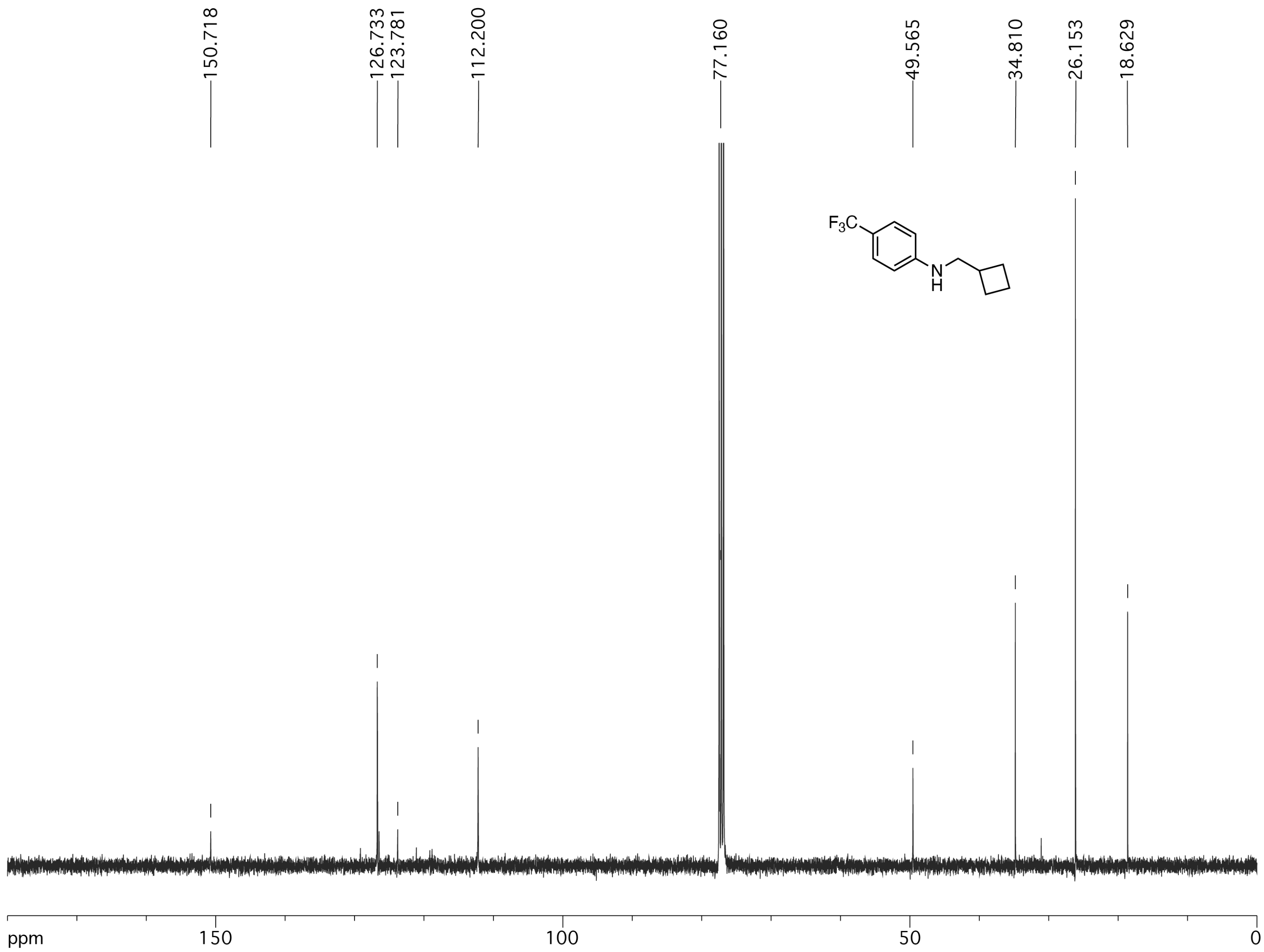

Figure S46. ^{13}C NMR spectrum of **5d** in $CDCl_3$ (100 MHz) at 23 °C.

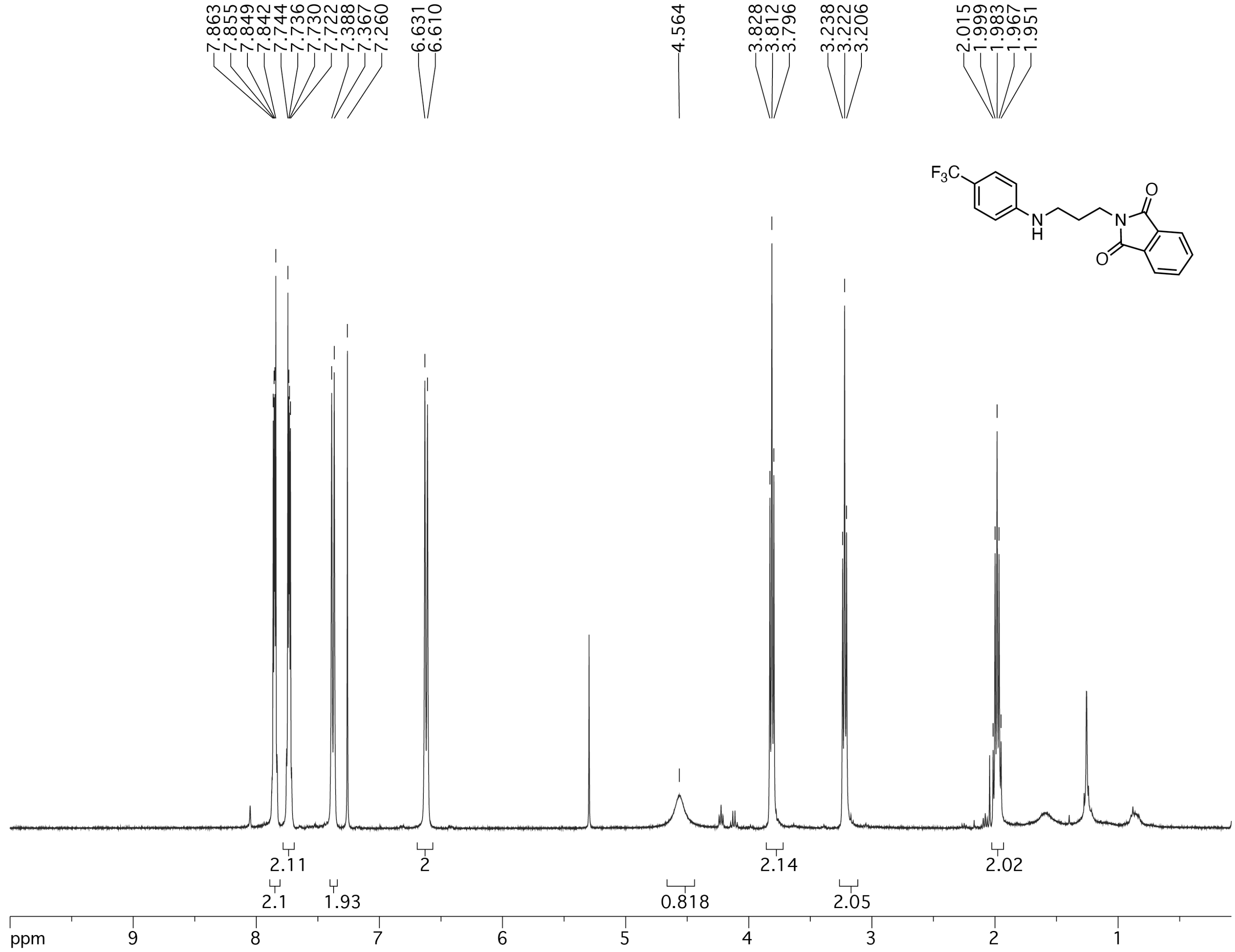

Figure S47. ^{1}H NMR spectrum of **5e** in $CDCl_3$ (400 MHz) at 23 °C.

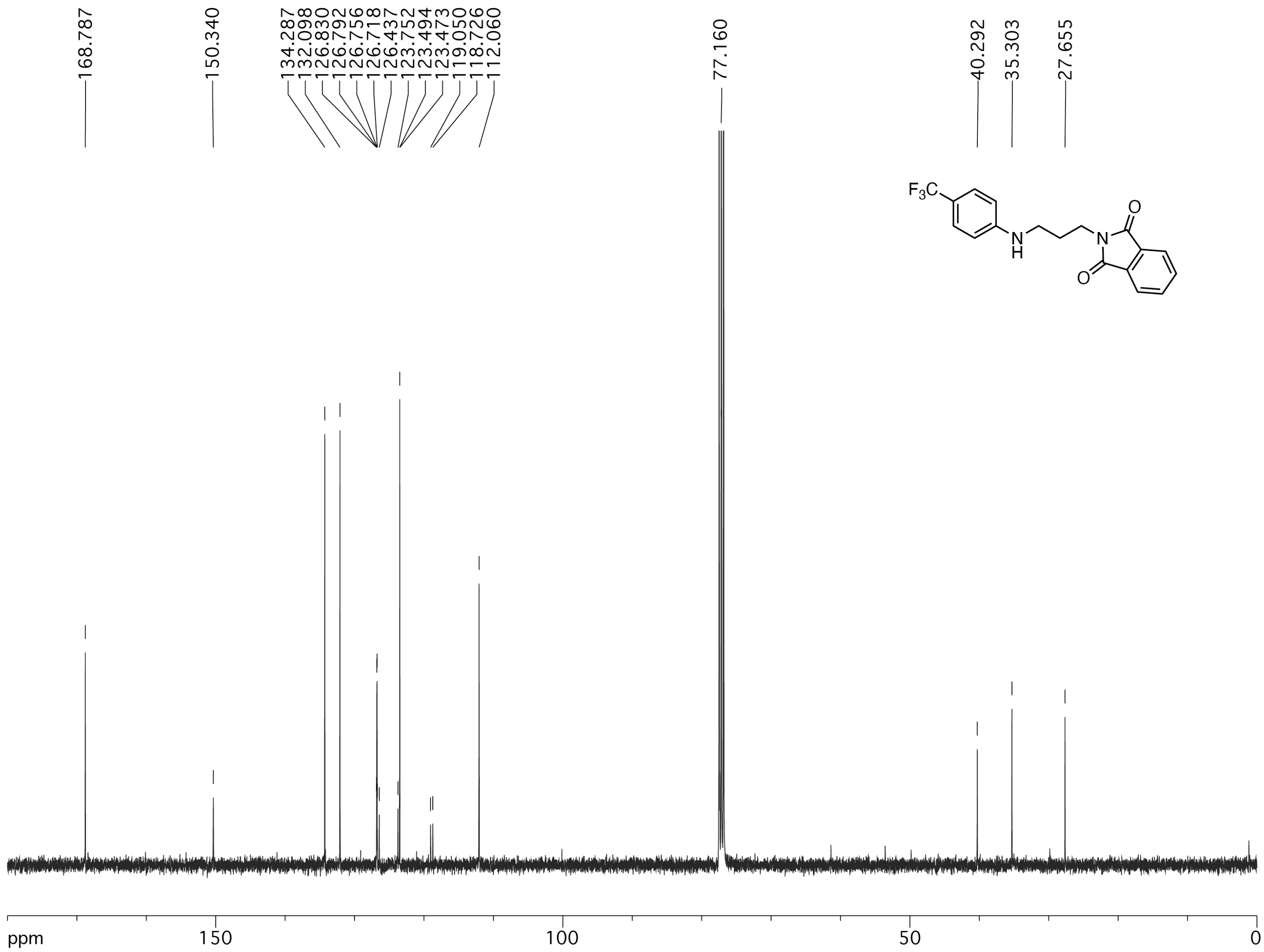

Figure S48. ^{13}C NMR spectrum of **5e** in $CDCl_3$ (100 MHz) at 23 °C.

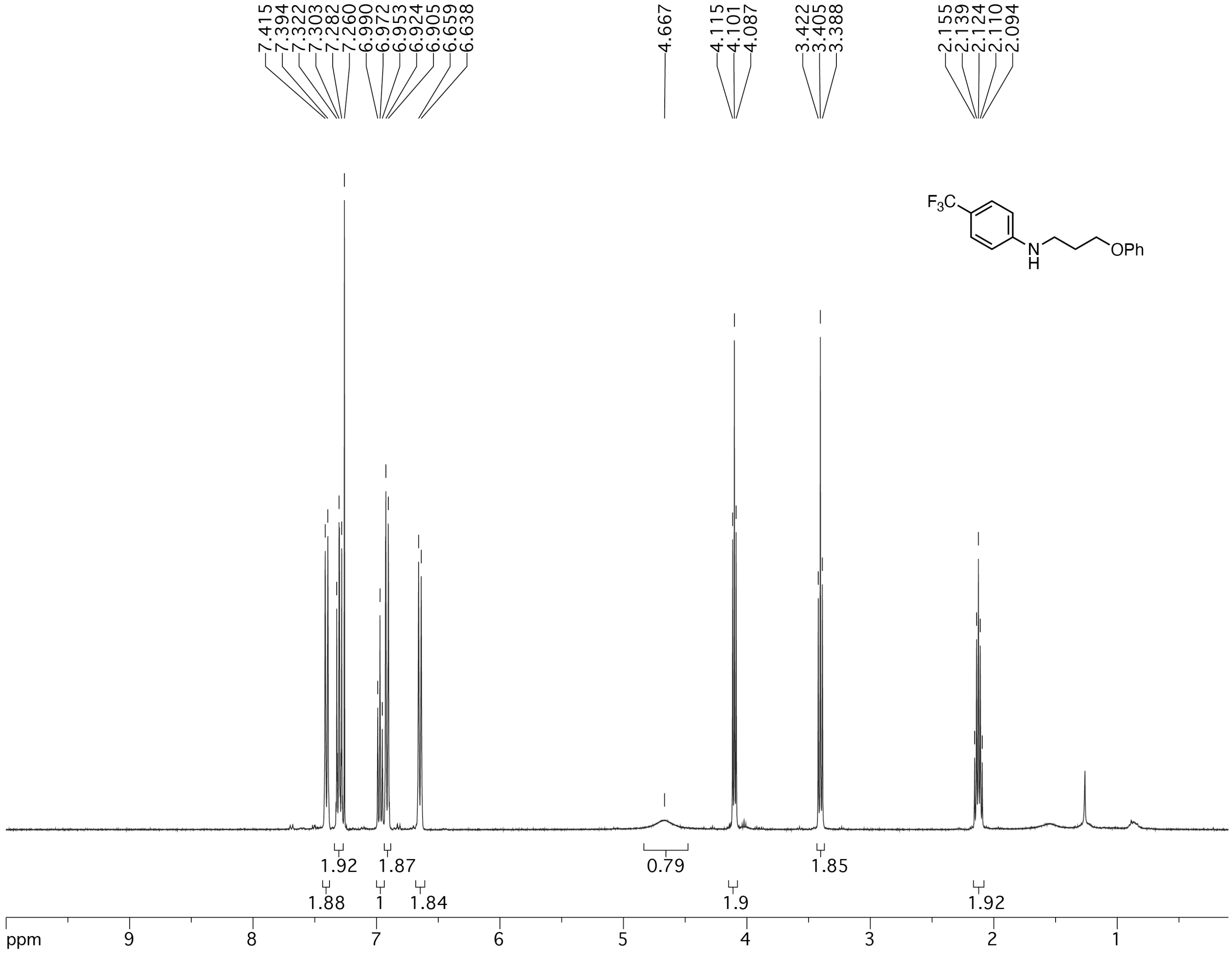

Figure S49. ^{1}H NMR spectrum of **5f** in $CDCl_3$ (400 MHz) at 23 °C.

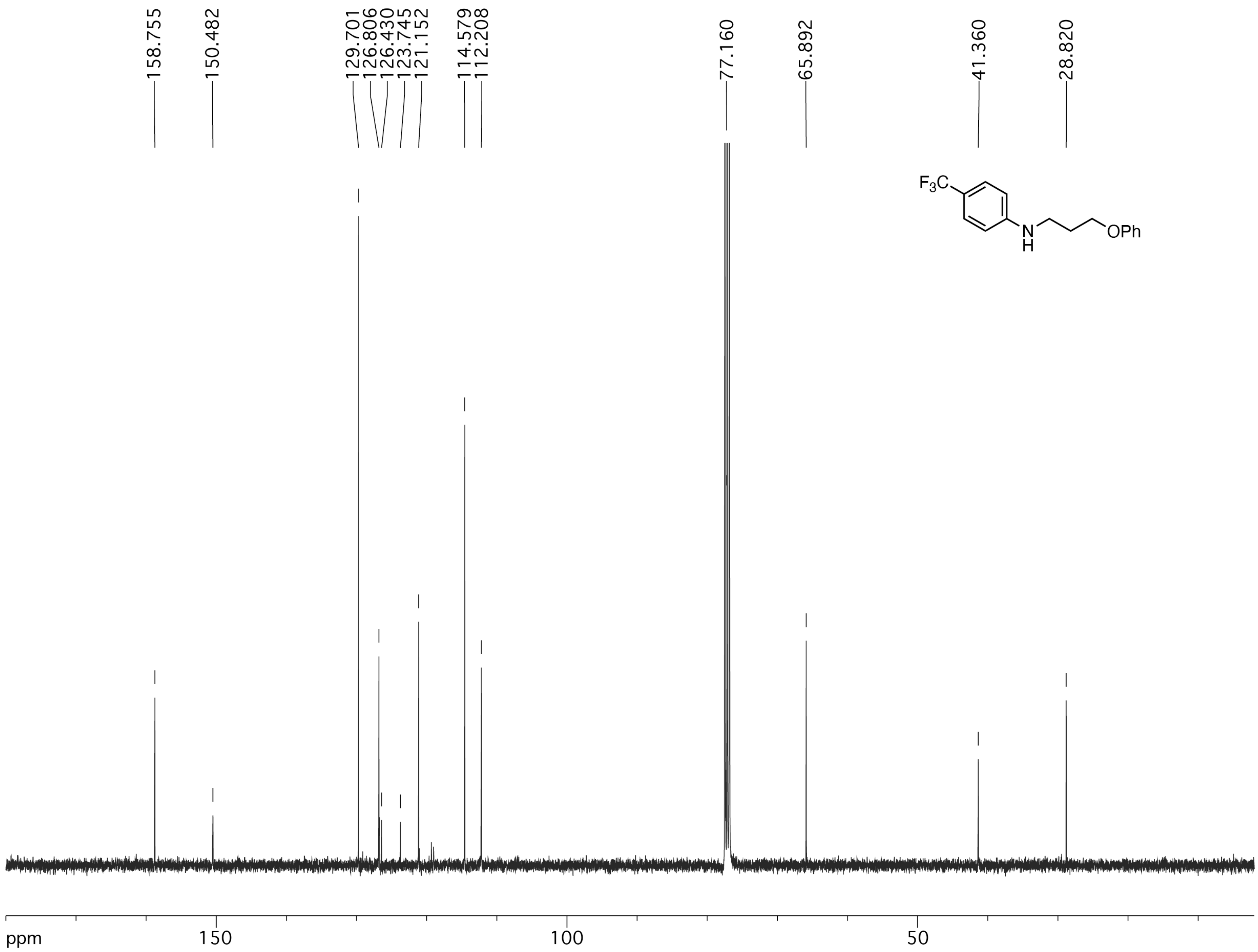

Figure S50. ^{13}C NMR spectrum of **5f** in $CDCl_3$ (100 MHz) at 23 °C.

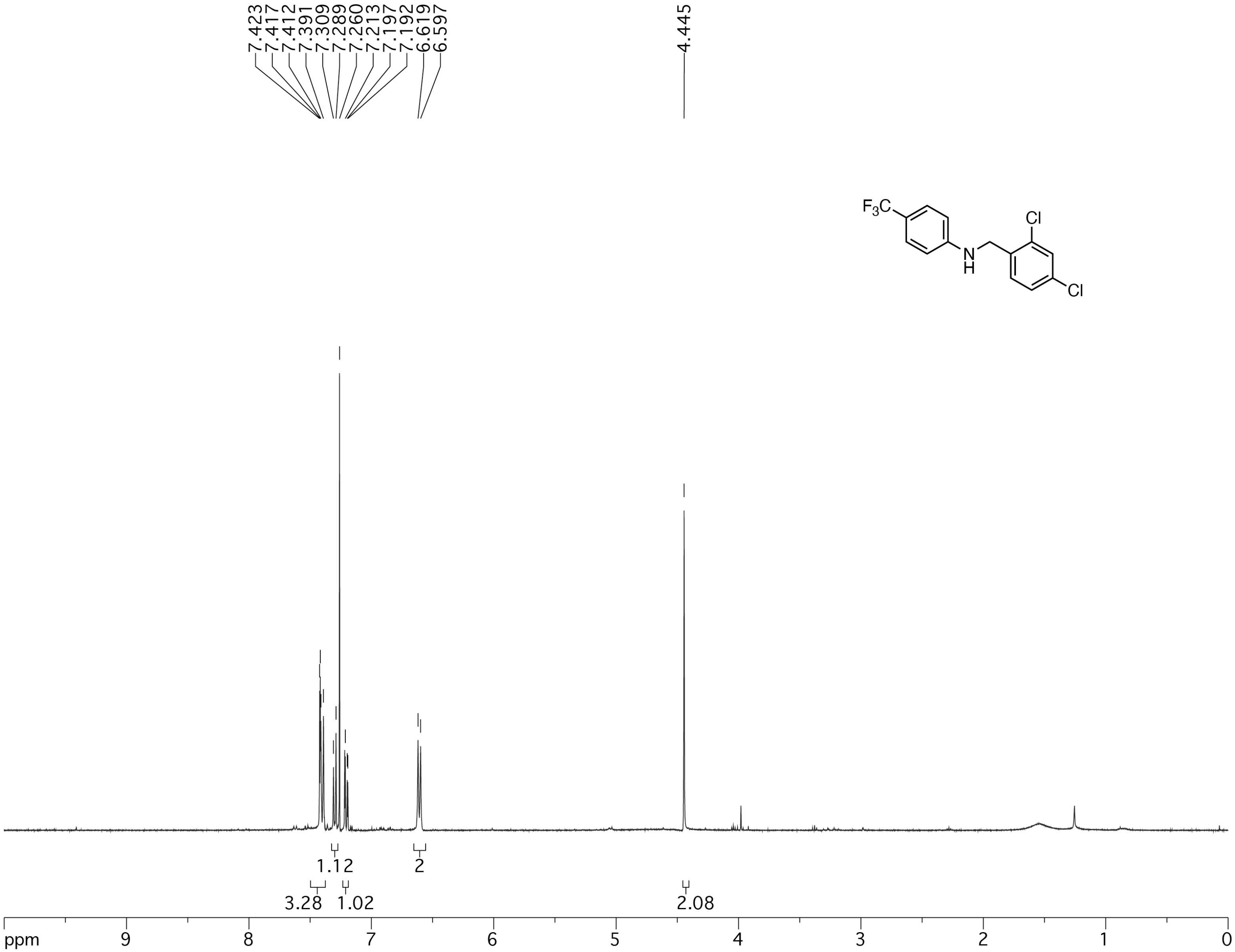

Figure S51. ^{1}H NMR spectrum of **5l** in $CDCl_3$ (400 MHz) at 23 °C.

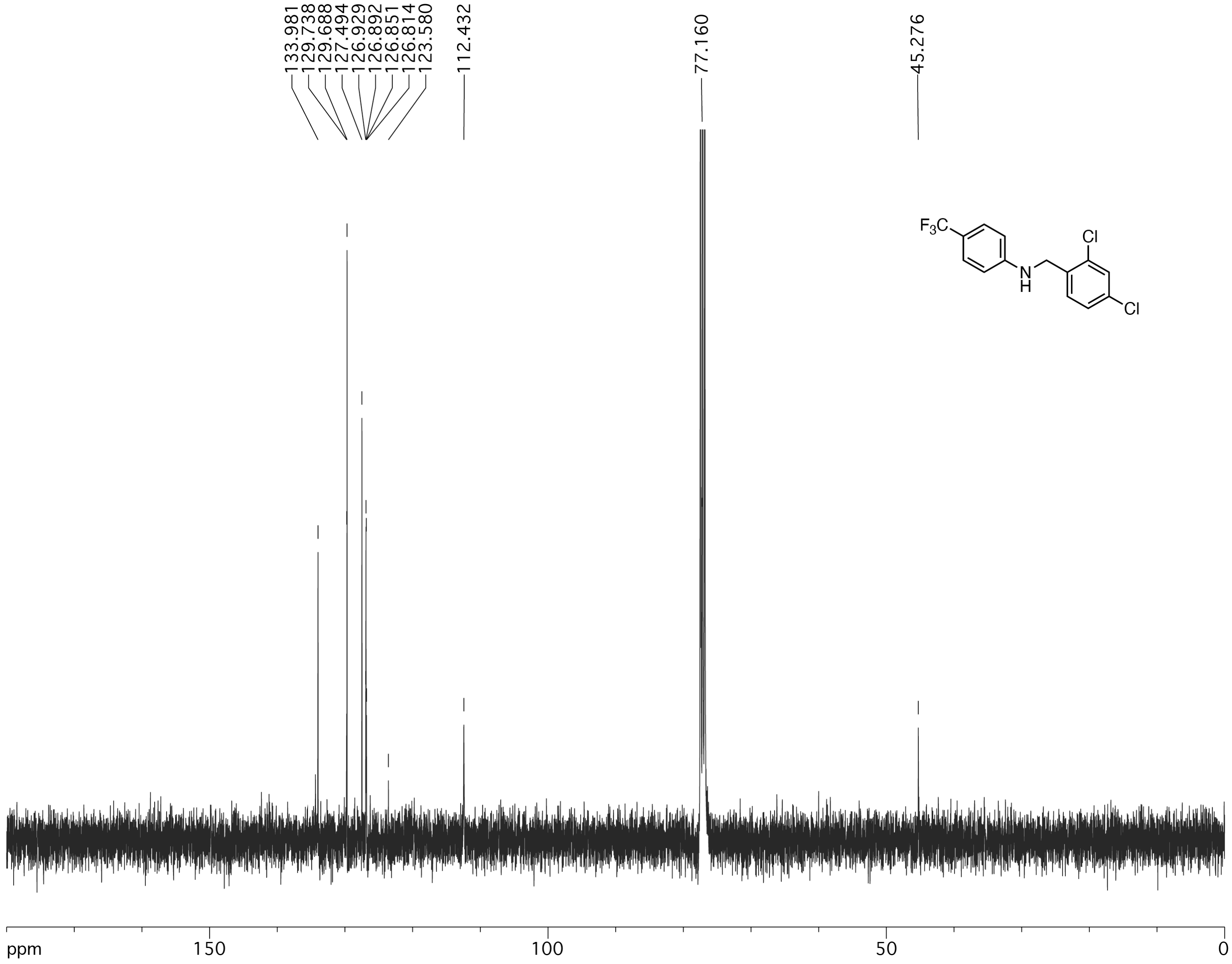

Figure S52. ^{13}C NMR spectrum of **5l** in $CDCl_3$ (100 MHz) at 23 °C.

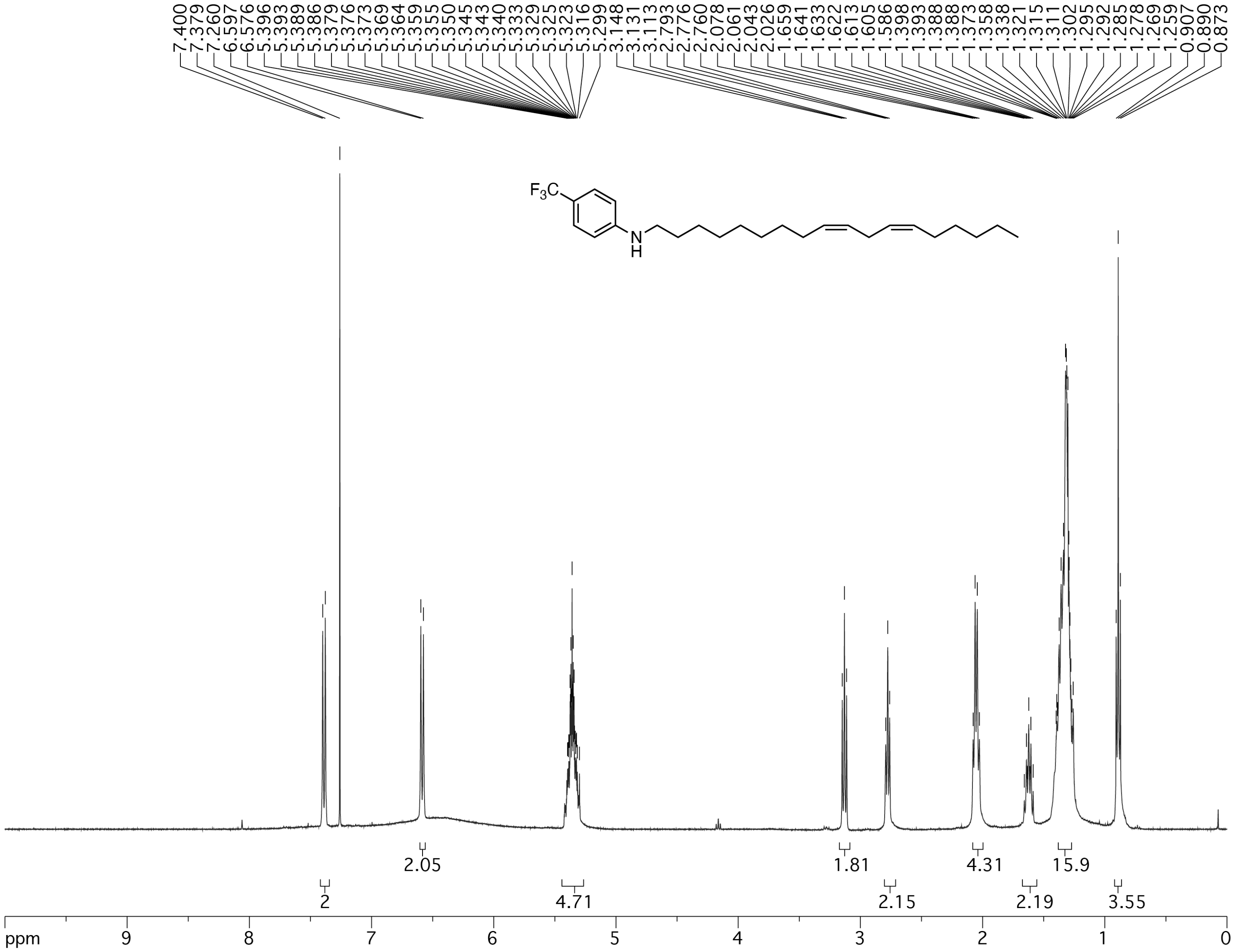

Figure S53. ^{1}H NMR spectrum of **5s** in $CDCl_3$ (400 MHz) at 23 °C.

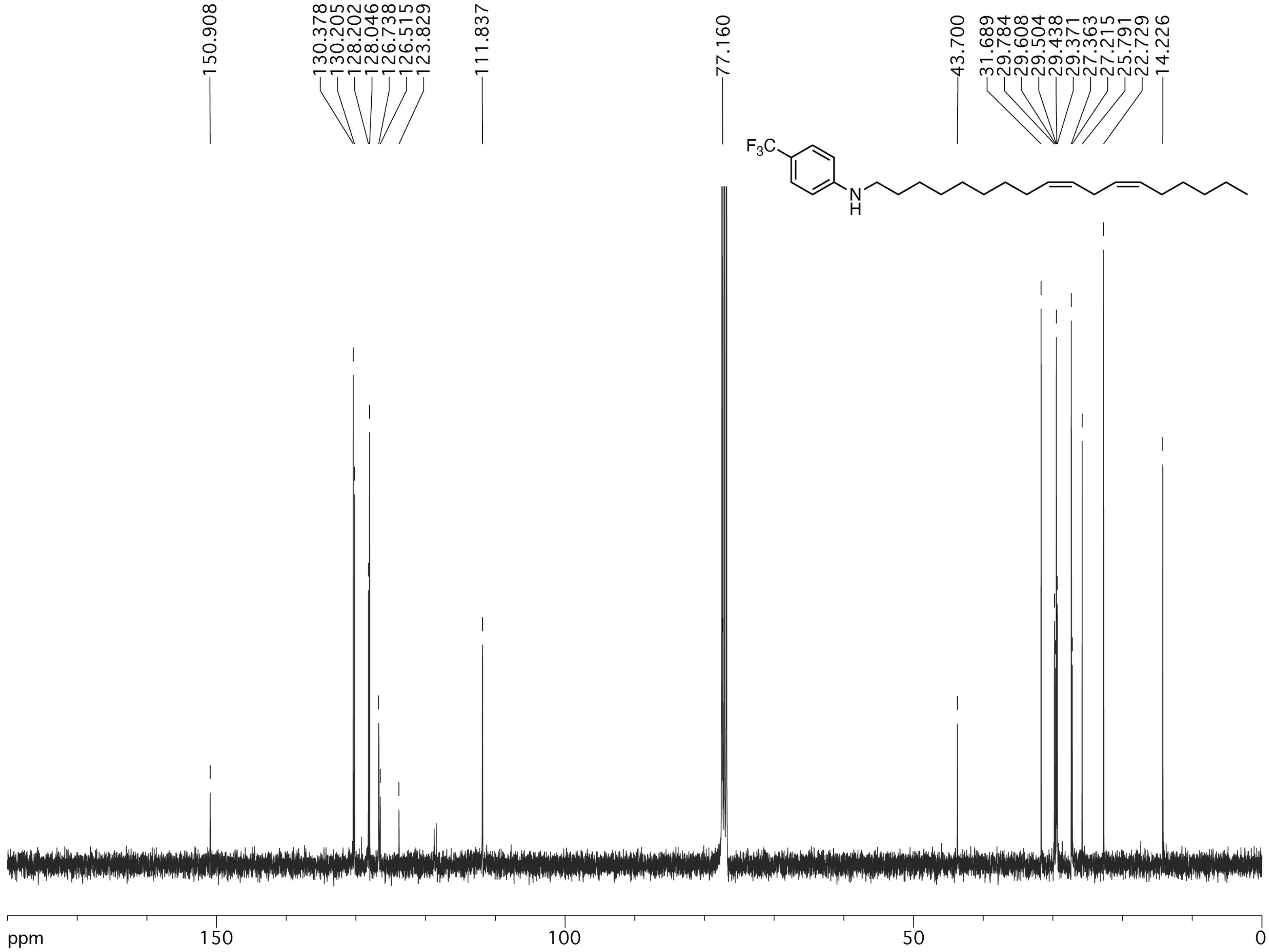

Figure S54. ^{13}C NMR spectrum of **5s** in $CDCl_3$ (100 MHz) at 23 °C.

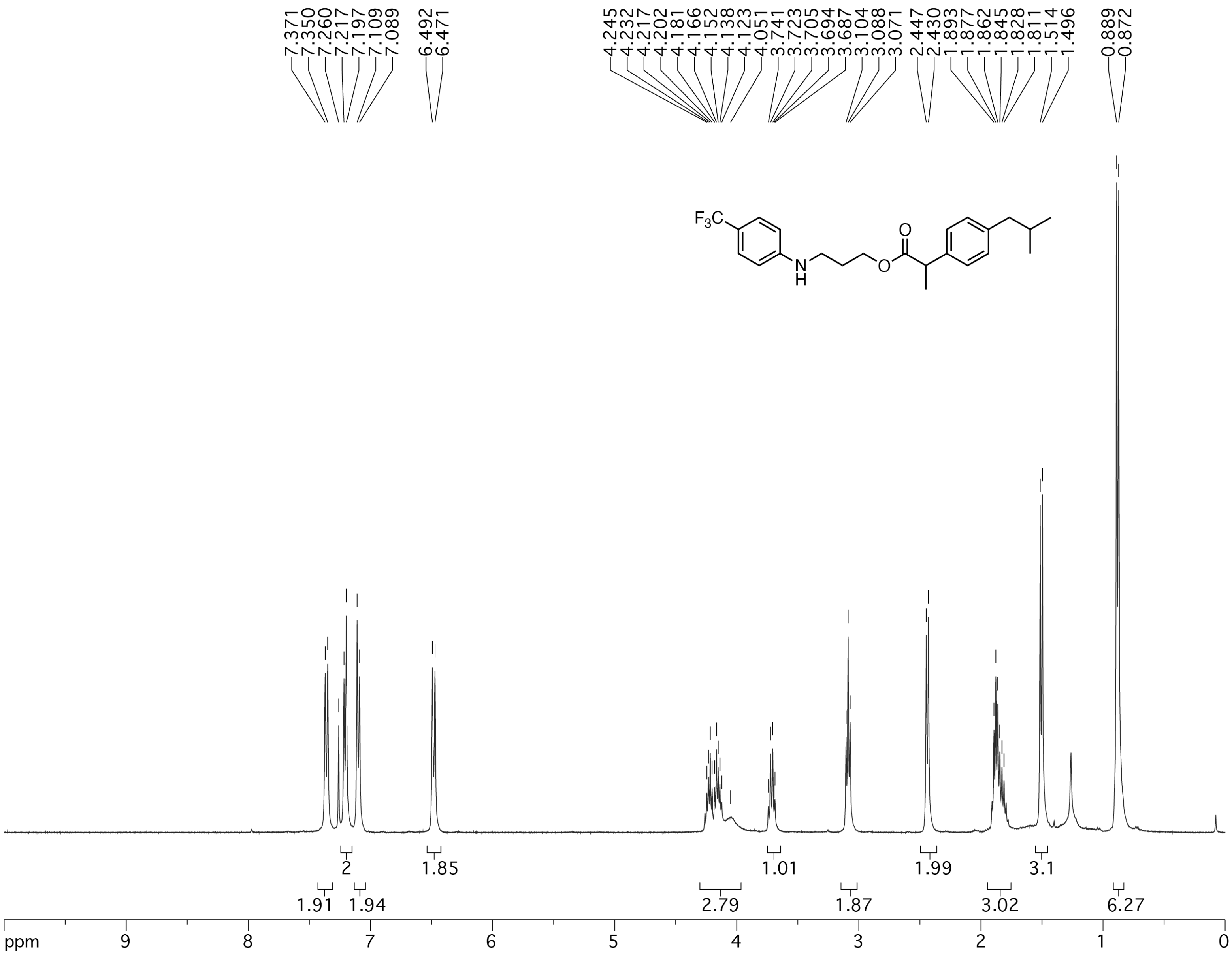

Figure S55. ^{1}H NMR spectrum of **5t** in $CDCl_3$ (400 MHz) at 23 °C.

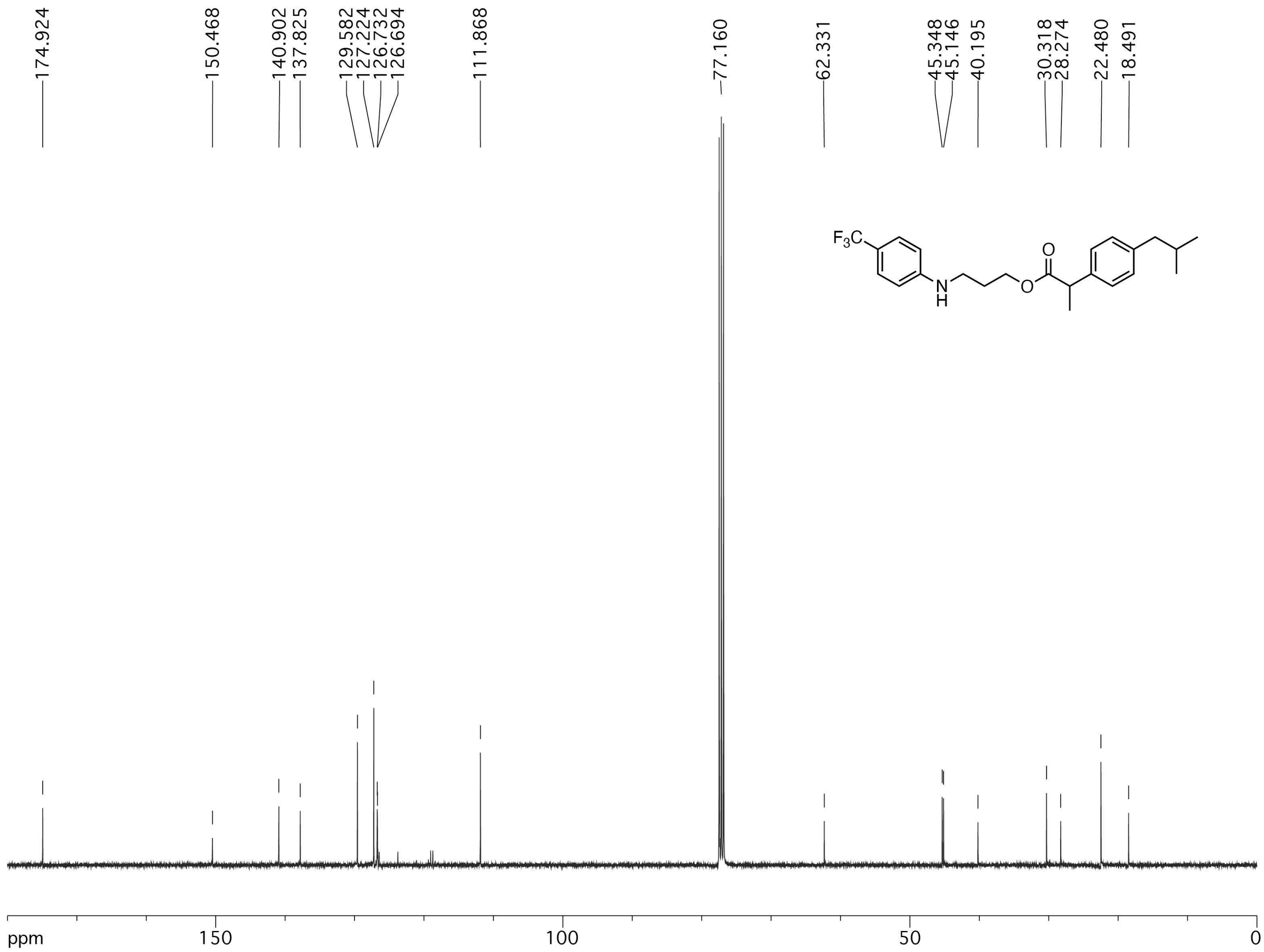

Figure S56. ^{13}C NMR spectrum of **5t** in $CDCl_3$ (100 MHz) at 23 °C.

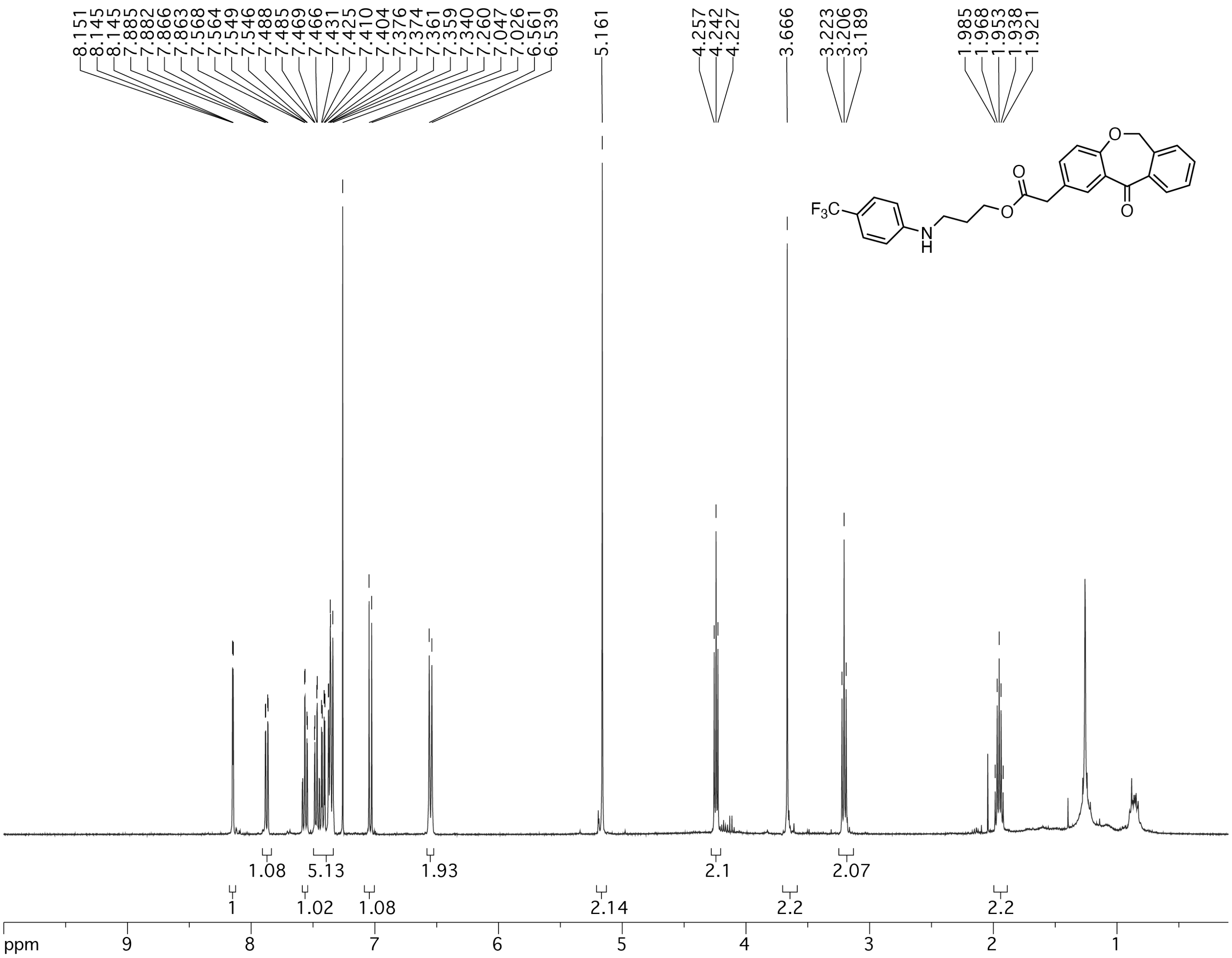

Figure S57. ^{1}H NMR spectrum of **5u** in $CDCl_3$ (400 MHz) at 23 °C.

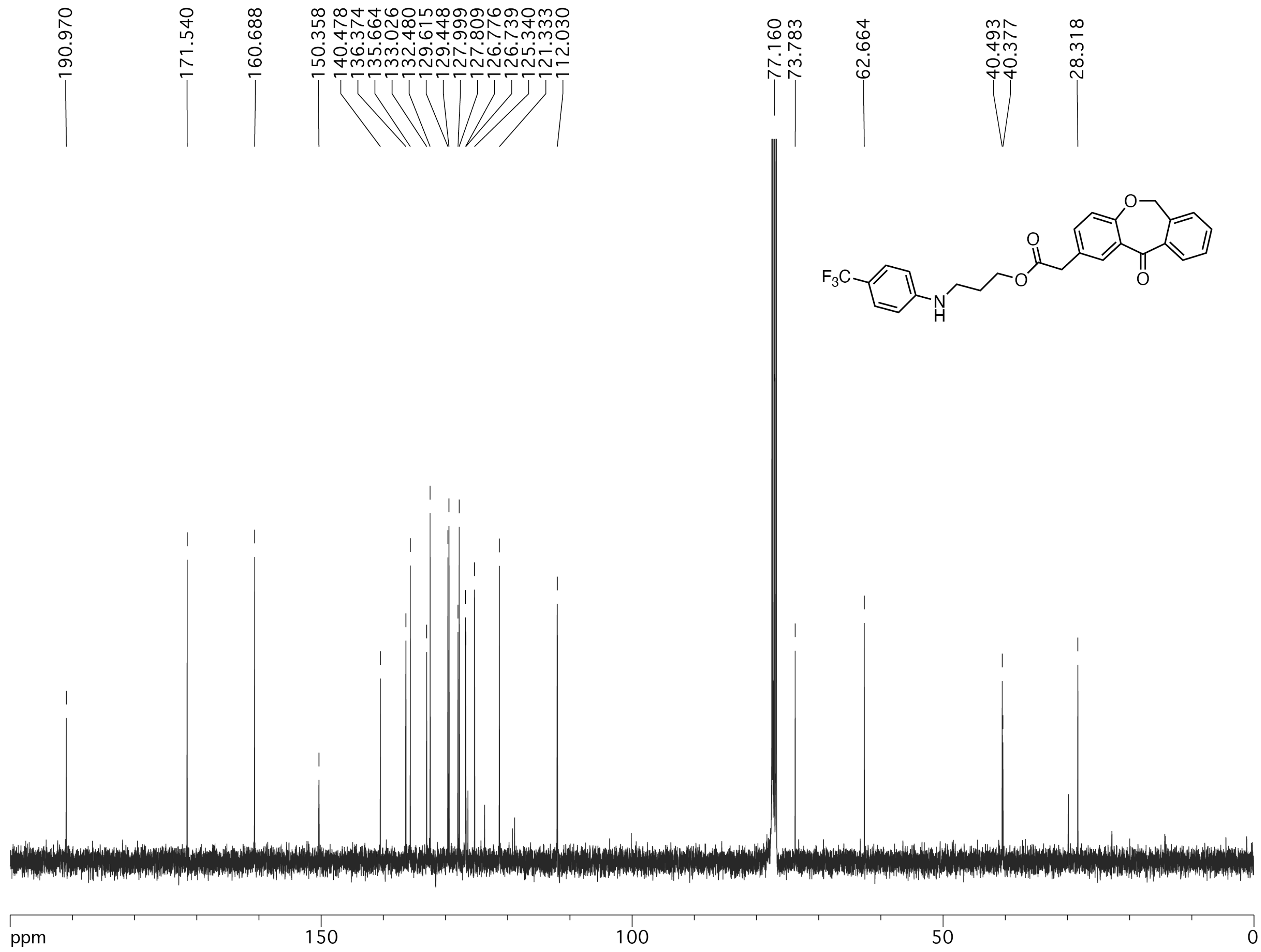

Figure S58. ^{13}C NMR spectrum of **5u** in $CDCl_3$ (100 MHz) at 23 °C.

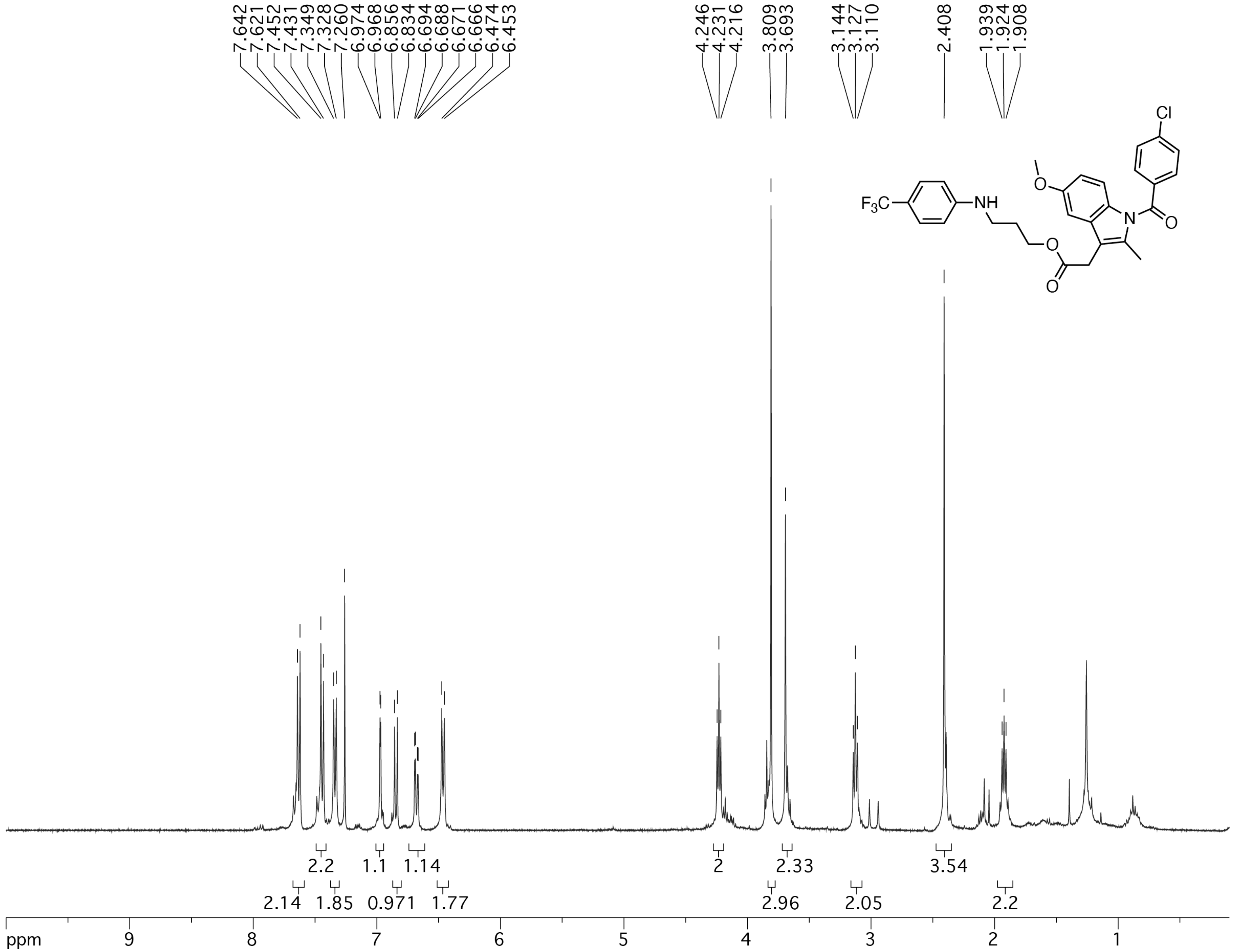

Figure S59. ^{1}H NMR spectrum of **5v** in $CDCl_3$ (400 MHz) at 23 °C.

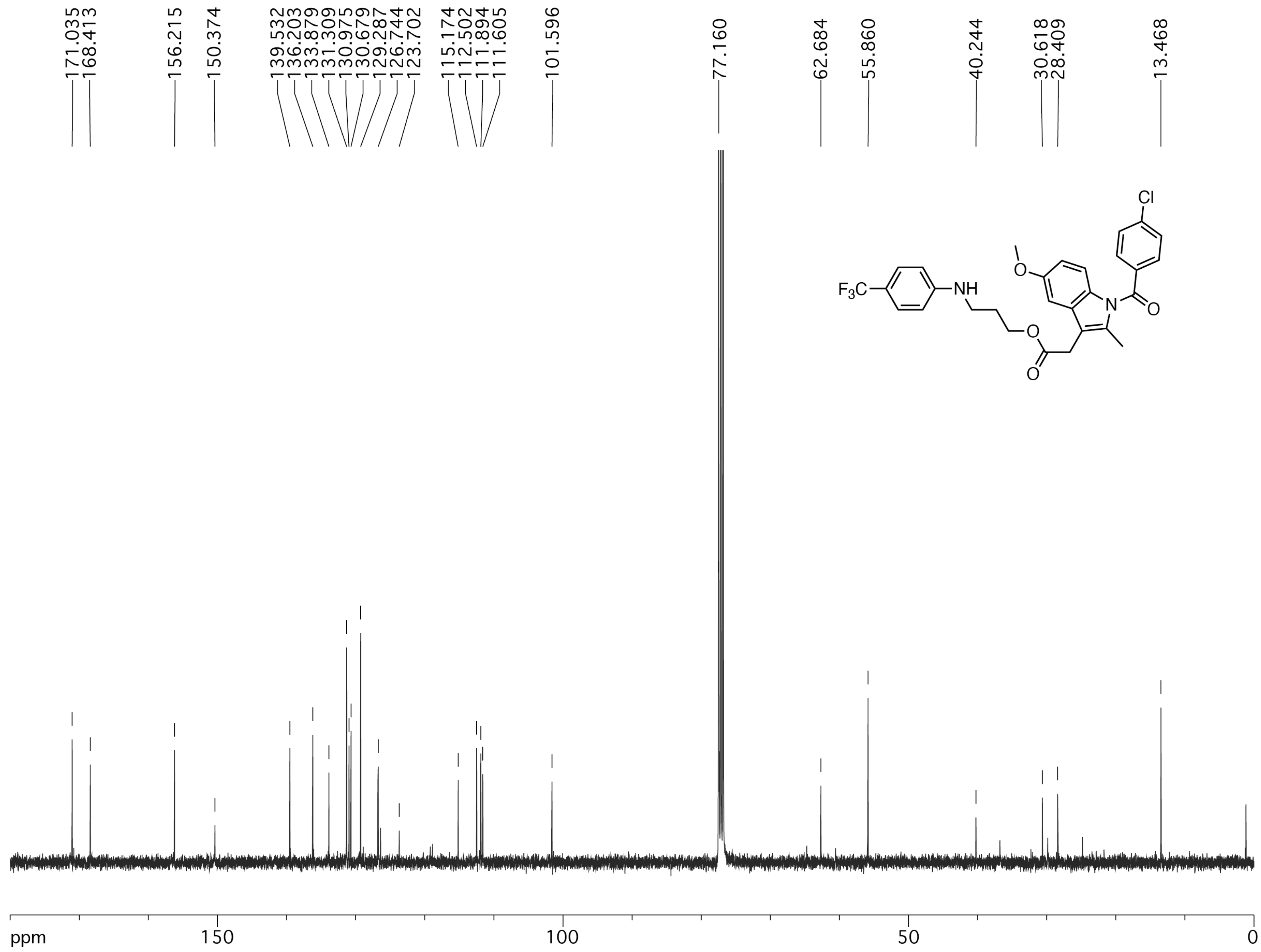

Figure S60. ^{13}C NMR spectrum of **5v** in $CDCl_3$ (100 MHz) at 23 °C.

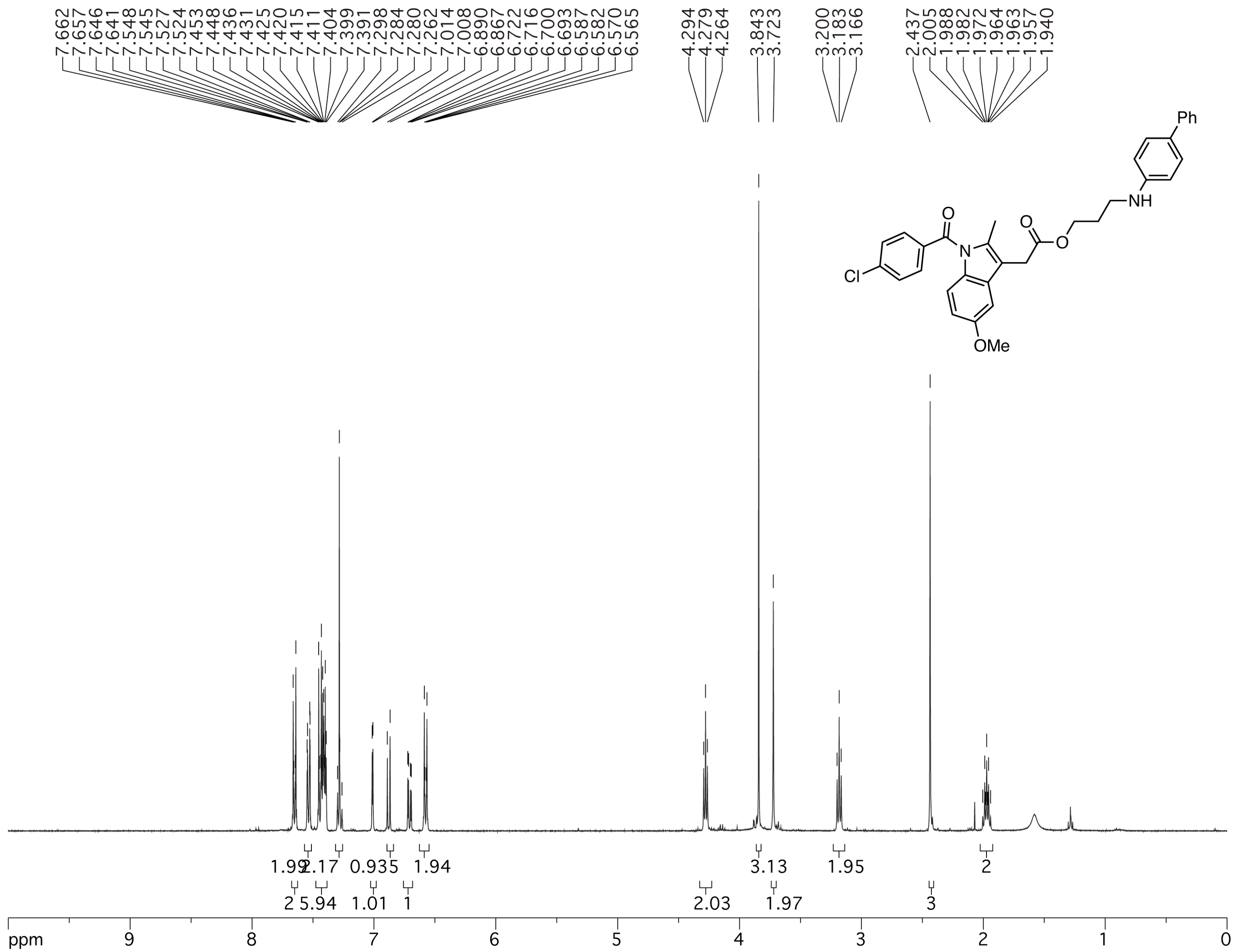

Figure S61. ^{1}H NMR spectrum of **5w** in $CDCl_3$ (400 MHz) at 23 °C.

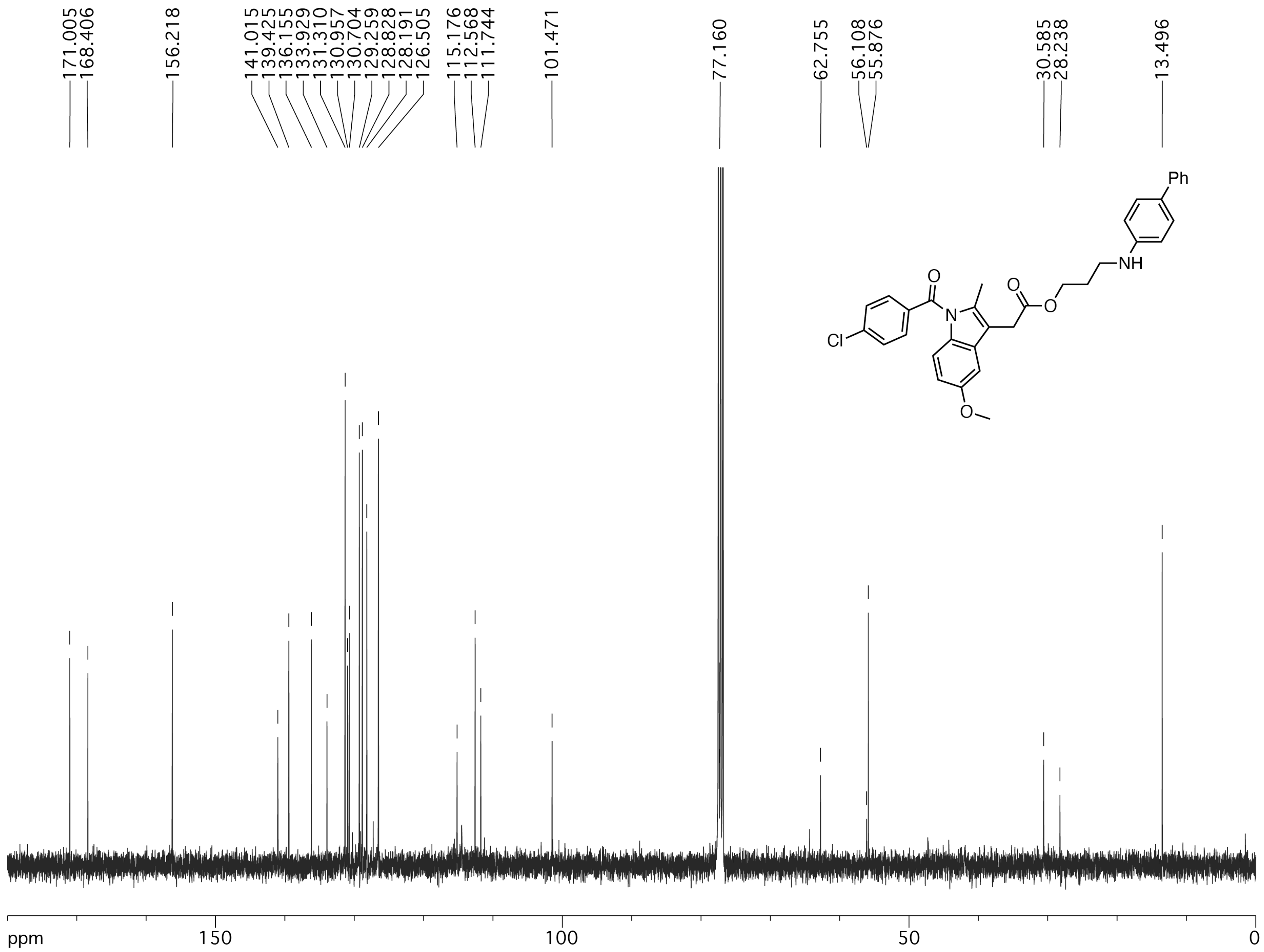

Figure S62. ^{13}C NMR spectrum of **5w** in $CDCl_3$ (100 MHz) at 23 °C.

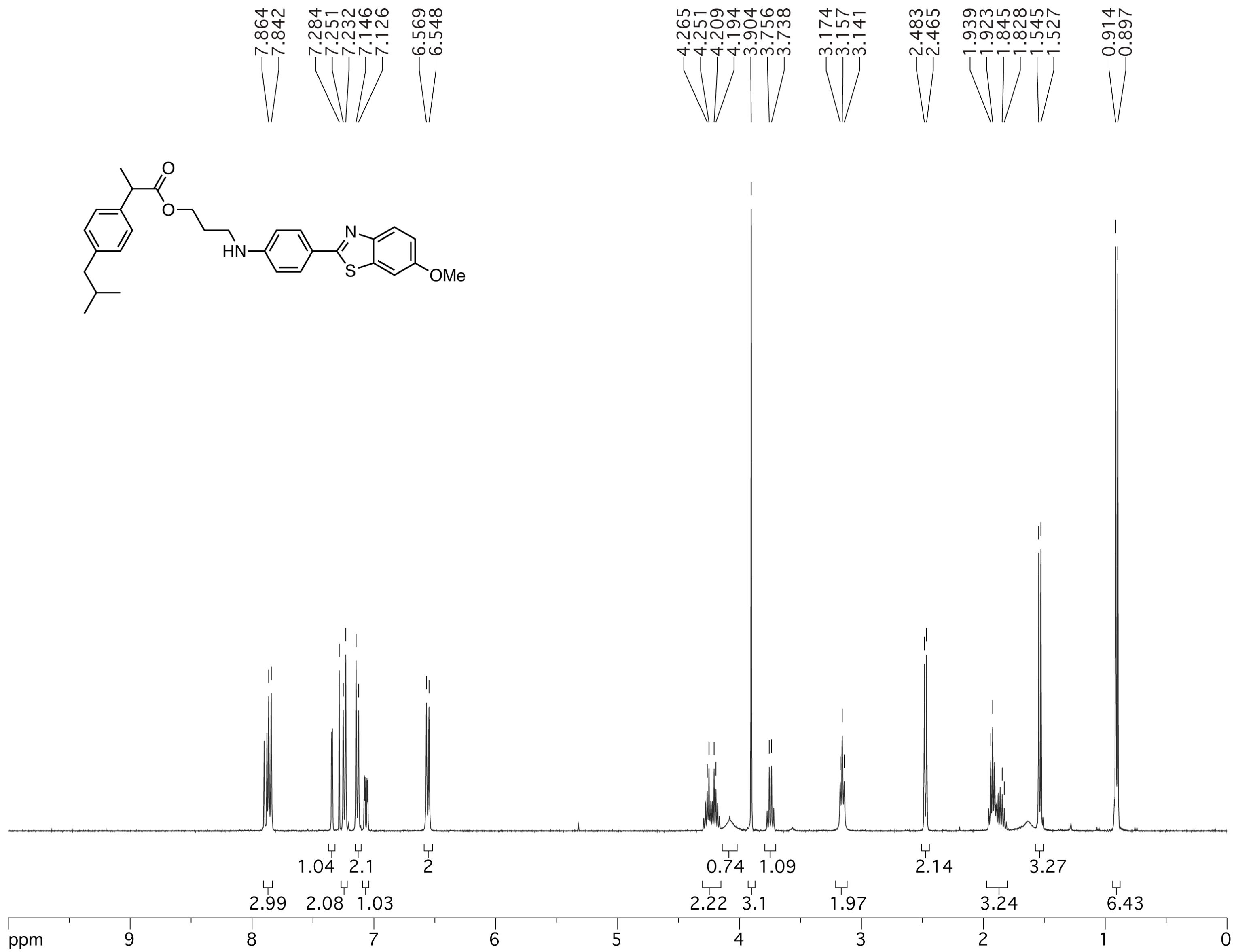

Figure S63. ^{1}H NMR spectrum of **5x** in $CDCl_3$ (400 MHz) at 23 °C.

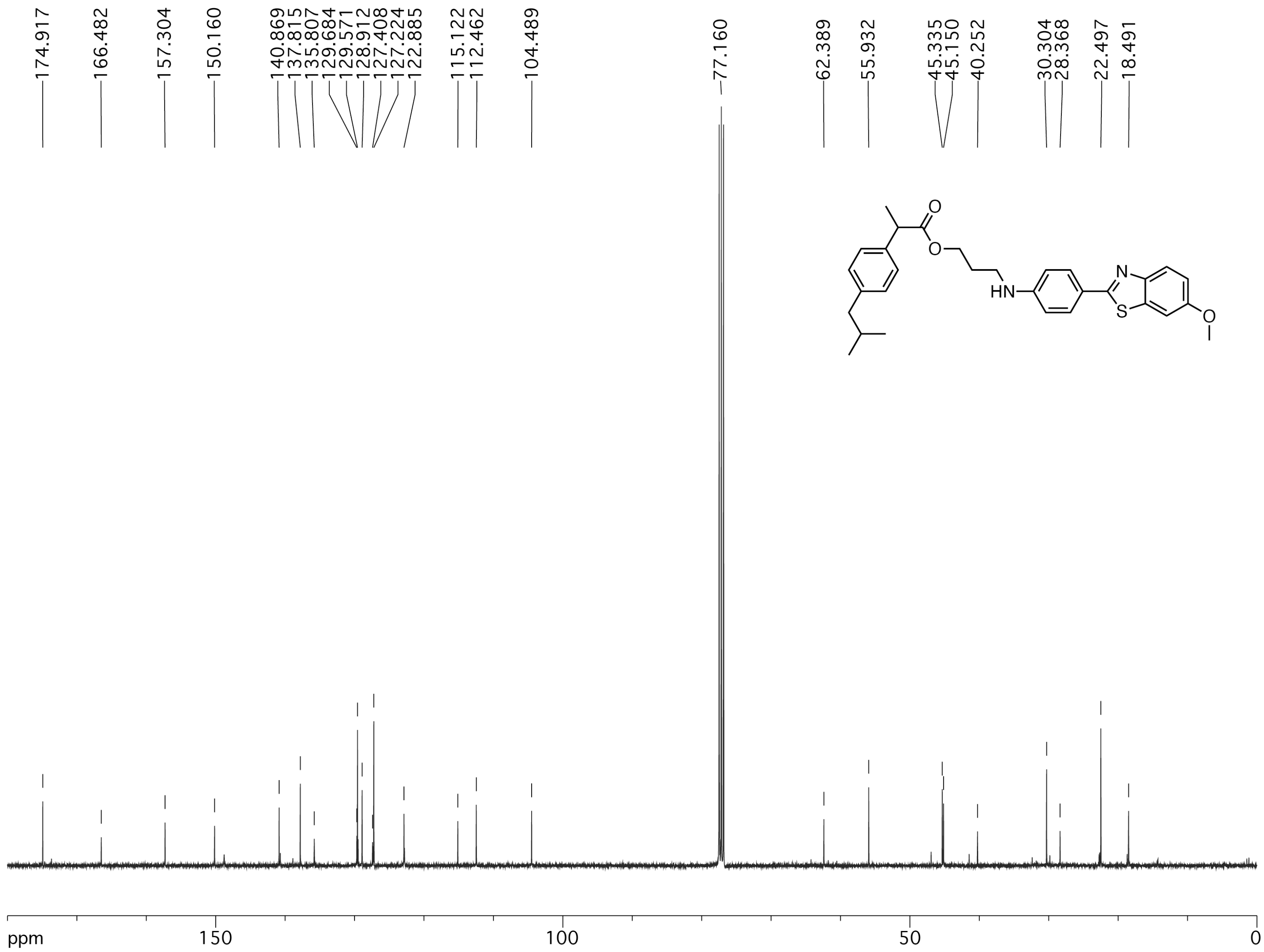

Figure S64. ^{13}C NMR spectrum of **5x** in $CDCl_3$ (100 MHz) at 23 °C.

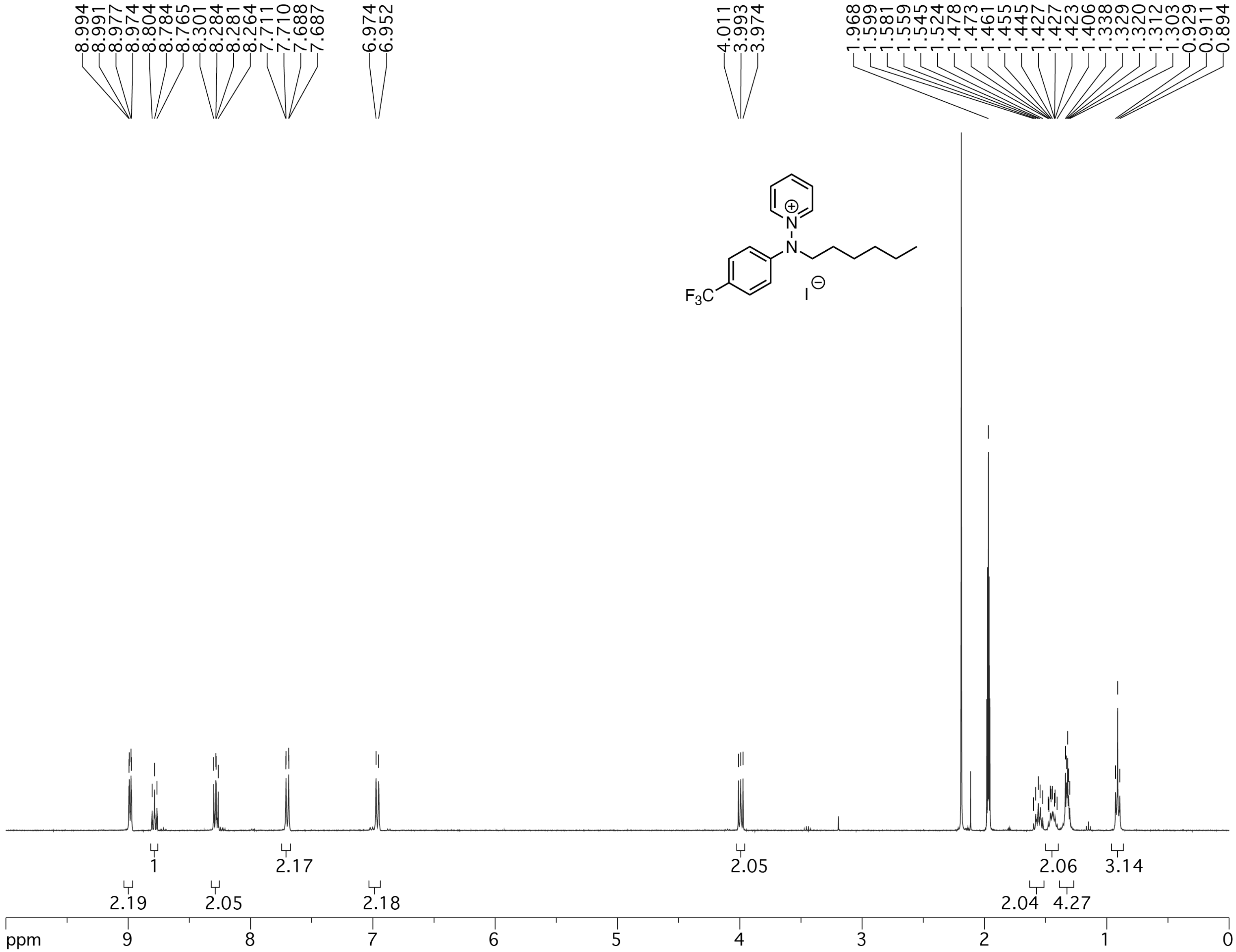

Figure S65. ^{1}H NMR spectrum of **5a'** in CD_3CN (400 MHz) at 23 °C.

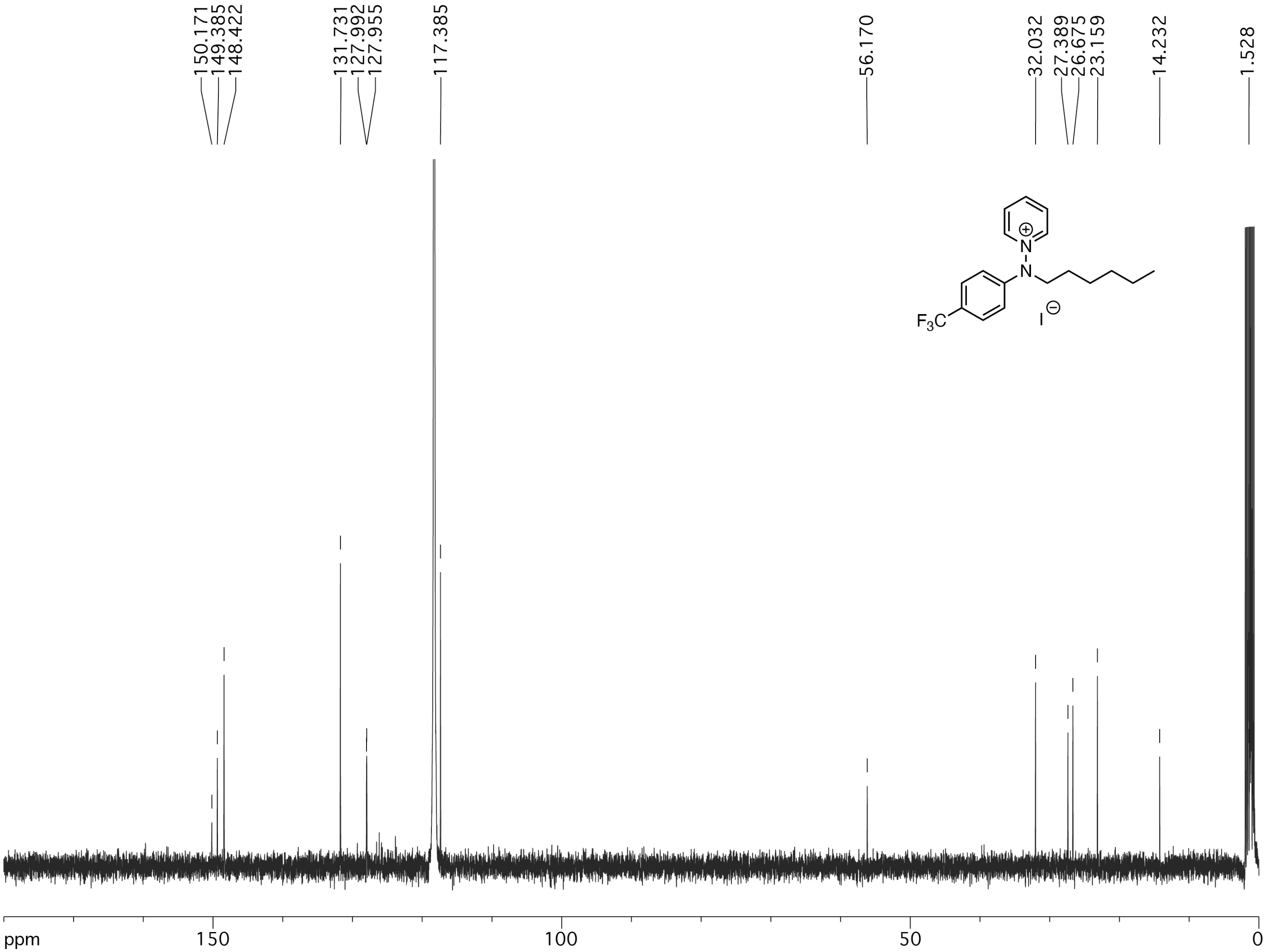

Figure S66. ^{13}C NMR spectrum of **5a’** in CD_3CN (100 MHz) at 23 °C.

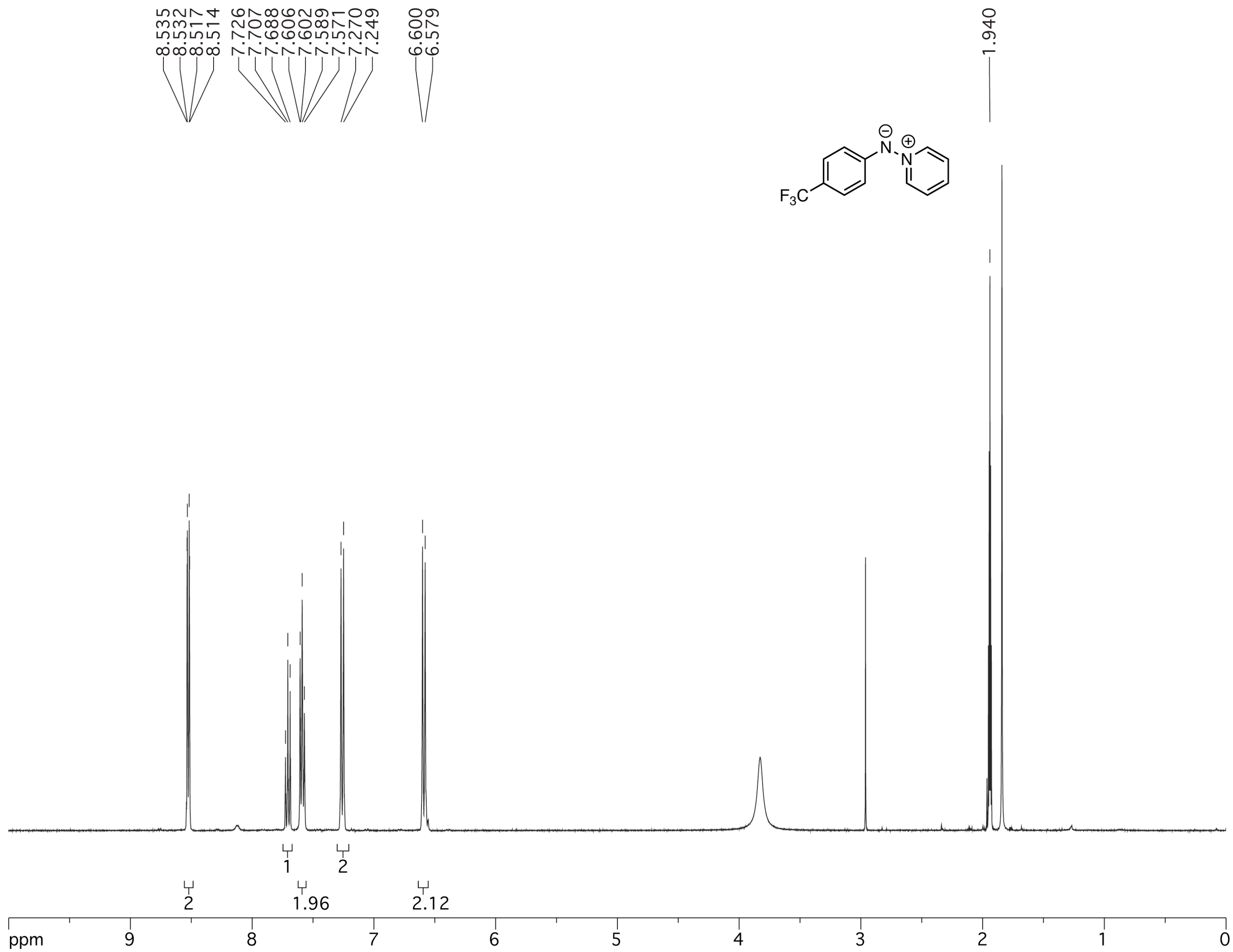

Figure S67. ^{1}H NMR spectrum of **3a'** in CD_3CN (400 MHz) at 23 °C.

G. References

1. A. B. Pangborn, M. A. Giardello, R. H. Grubbs, R. K. Rosen and F. J. Timmers, Safe and convenient procedure for solvent purification, *Organometallics*, 1996, **15**, 1518–1520.
2. P. Roychowdhury, R. G. Herrera, H. Tan and D. C. Powers, Traceless Benzylic C−H Amination via Bifunctional N-Aminopyridinium Intermediates, *Angew. Chem. Int. Ed.,* 2022, **61**, e202200665.
3. S.-T. Kim, M. J. Strauss, A. Cabré and S. L. Buchwald, Room-Temperature Cu-Catalyzed Amination of Aryl Bromides Enabled by DFT-Guided Ligand Design, *J. Am. Chem. Soc.*, 2023, **145**, 6966–6975.
4. T. J. Graham and A. G. Doyle, Nickel-catalyzed cross-coupling of chromene acetals and boronic acids, *Org. Lett.*, 2012, **14**, 1616–1619.
5. G. Li, Z. Qin and A. T. Radosevich, P(III)/P(V)-catalyzed methylamination of arylboronic acids and esters: reductive C–N coupling with nitromethane as a methylamine surrogate, *J. Am. Chem. Soc.*, 2020, **142**, 16205–16210.
6. R. K. Bowman and J. S. Johnson, Nickel-Catalyzed Rearrangement of 1-Acyl-2-vinylcyclopropanes. A Mild Synthesis of Substituted Dihydrofurans, *Org. Lett.*, 2006, **8**, 573–576.
7. B. A. Cotrim, J. Joglar, M. J. L. Rojas, J. M. D. del Olmo, M. Macias-González, M. R. Cuevas, M. Fitó, D. Muñoz-Aguayo, M. I. Covas Planells, M. Farré, F. Rodríguez de Fonseca and R. de la Torre, Unsaturated Fatty Alcohol Derivatives of Olive Oil Phenolic Compounds with Potential Low-Density Lipoprotein (LDL) Antioxidant and Antiobesity Properties, *J. Agric. Food Chem.*, 2012, **60**, 1067–1074.
8. B. P. Bandgar, V. S. Sadavarte and L. S. Uppalla, An expedient and highly selective iodination of alcohols using a $KI/BF_3 \cdot Et_2O$ system, *Tetrahedron Lett.*, 2001, **42**, 951–953.
9. J. J. López and E. G. Pérez, New convergent one pot synthesis of amino benzyl ethers bearing a nitrogen-containing bicycle, *Synth. Commun.*, 2019, **49**, 715–723.
10. S. Bera and X. Hu, Nickel-Catalyzed Regioselective Hydroalkylation and Hydroarylation of Alkenyl Boronic Esters, *Angew. Chem. Int. Ed.*, 2019, **58**, 13854–13859.
11. O. V. Dolomanov, L. J. Bourhis, R. J. Gildea, J. A. Howard and H. Puschmann, OLEX2: a complete structure solution, refinement and analysis program, *J. Appl. Crystallogr.*, 2009, **42**, 339–341.
12. G. M. Sheldrick, A short history of SHELX, *Acta Crystallogr. A*, 2008, **64**, 112–122.
13. G. M. Sheldrick, Crystal structure refinement with SHELXL, *Acta Crystallogr. C*, 2015, **71**, 3–8.
14. S. L. Goldschmid, N. E. Soon Tay, C. L. Joe, B. C. Lainhart, T. C. Sherwood, E. M. Simmons, M. Sezen-Edmonds and T. Rovis, Overcoming Photochemical Limitations in Metallaphotoredox Catalysis: Red-Light-Driven C–N Cross-Coupling, *J. Am. Chem. Soc.*, 2022, **144**, 22409–22415.
15. B. Zhao, M. Wang and Z. Shi, Single-Electron-Transfer-Induced C(sp^3)–N Couplings via C–C Bond Cleavage of Cycloketoxime Esters, *J. Org. Chem.*, 2019, **84**, 10145–10159.
16. O. Löber, M. Kawatsura and J. F. Hartwig, Palladium-Catalyzed Hydroamination of 1,3-Dienes: A Colorimetric Assay and Enantioselective Additions, *J. Am. Chem. Soc.*, 2001, **123**, 4366–4367.

17. Q. Yin, Y. Soltani, R. L. Melen and M. Oestreich, $BArF_3$-Catalyzed Imine Hydroboration with Pinacolborane Not Requiring the Assistance of an Additional Lewis Base, *Organometallics*, 2017, **36**, 2381–2384.
18. V. T. Nguyen, V. D. Nguyen, G. C. Haug, N. T. H. Vuong, H. T. Dang, H. D. Arman and O. V. Larionov, Visible-Light-Enabled Direct Decarboxylative N-Alkylation, *Angew. Chem. Int. Ed.*, 2020, **59**, 7921–7927.
19. S. Li, T. Rajeshkumar, J. Liu, L. Maron and X. Zhou, La-Catalyzed Decarbonylation of Formamides and Its Applications, *Org. Lett.*, 2023, **25**, 163–168.
20. D. Banerjee, R. V. Jagadeesh, K. Junge, H. Junge and M. Beller, An Efficient and Convenient Palladium Catalyst System for the Synthesis of Amines from Allylic Alcohols, *ChemSusChem*, 2012, **5**, 2039–2044.
21. M.-C. Fu, R. Shang, W.-M. Cheng and Y. Fu, Boron-Catalyzed *N*-Alkylation of Amines using Carboxylic Acids, *Angew. Chem. Int. Ed.*, 2015, **54**, 9042–9046.
22. S. Doherty, J. G. Knight, J. P. McGrady, A. M. Ferguson, N. A. B. Ward, R. W. Harrington and W. Clegg, ortho,ortho′-Substituted KITPHOS Monophosphines: Highly Efficient Ligands for Palladium-Catalyzed C−C and C−N Bond Formation, *Adv. Synth. Catal.*, 2010, **352**, 201–211.
23. Y.-B. Huang, C.-T. Yang, J. Yi, X.-J. Deng, Y. Fu and L. Liu, Cu-Catalyzed Carbon-Heteroatom Coupling Reactions under Mild Conditions Promoted by Resin-Bound Organic Ionic Bases, *J. Org. Chem.*, 2011, **76**, 800–810.
24. G. Li, Z. Qin and A. T. Radosevich, P(III)/P(V)-Catalyzed Methylamination of Arylboronic Acids and Esters: Reductive C–N Coupling with Nitromethane as a Methylamine Surrogate, *J. Am. Chem. Soc.*, 2020, **142**, 16205–16210.
